# Mechanistic Studies on Aluminum-Catalyzed Ring-Opening Alternating Copolymerization of Maleic Anhydride with Epoxides: Ligand Effects and Quantitative Structure-Activity Relationship Model

**DOI:** 10.3390/molecules28217279

**Published:** 2023-10-26

**Authors:** Xiaowei Xu, Hao Li, Andleeb Mehmood, Kebin Chi, Dejun Shi, Zhuozheng Wang, Bin Wang, Yuesheng Li, Yi Luo

**Affiliations:** 1 PetroChina Petrochemical Research Institute, Beijing 102206, China; 2 College of Physics and Optoelectronic Engineering, Shenzhen University, Shenzhen 518000, China; 3 Tianjin Key Laboratory of Composite & Functional Materials, School of Materials Science and Engineering, Tianjin University, Tianjin 300350, China

**Keywords:** aluminum complex, density functional theory, ring-opening alternating copolymerization, ligand effect, multivariate linear regression

## Abstract

Previous work has indicated that aluminum (Al) complexes supported by a bipyridine bisphenolate (BpyBph) ligand exhibit higher activity in the ring-opening copolymerization (ROCOP) of maleic anhydride (MAH) and propylene oxide (PO) than their salen counterparts. Such a ligand effect in Al-catalyzed MAH-PO copolymerization reactions has yet to be clarified. Herein, the origin and applicability of the ligand effect have been explored by density functional theory, based on the mechanistic analysis for chain initiation and propagation. We found that the lower LUMO energy of the (BpyBph)AlCl complex accounts for its higher activity than the (salen)AlCl counterpart in MAH/epoxide copolymerizations. Inspired by the ligand effect, a structure-energy model was further established for catalytic activity (TOF value) predictions. It is found that the LUMO energies of aluminum chloride complexes and their average NBO charges of coordinating oxygen atoms correlate with the catalytic activity (TOF value) of Al complexes (R^2^ value of 0.98 and ‘3-fold’ cross-validation Q^2^ value of 0.88). This verified that such a ligand effect is generally applicable in anhydride/epoxide ROCOP catalyzed by aluminum complex and provides hints for future catalyst design.

## 1. Introduction

Aliphatic polyesters have drawn a lot of attention as a potentially viable choice for petroleum-based polymers because of their numerous renewable sources, hydrolytic degradability, and excellent biocompatibility [1,2,3]. In this context, the ring-opening copolymerization (ROCOP) of epoxides with anhydrides is a burgeoning technology to produce aliphatic polyesters [4,5,6]. The ROCOP of epoxides with anhydrides most commonly uses binary catalyst systems comprising metal complexes and nucleophilic cocatalyst components. The earliest epoxide/anhydride copolymerizations were initiated with amines and metal alkoxides, yielding low-molecular-weight polymers with broad dispersity and, in many cases, significant polyether contamination from epoxide homopolymerization [7,8].

The first well-controlled ROCOP of propylene oxide with phthalic anhydride catalyzed by aluminum porphyrin complex and a tetralkyl ammonium halide was reported in 1985 [9]. Subsequently, Coates and co-workers found that a *β*-diiminate zinc acetate [(BDI)ZnOAc] complex is an effective catalyst for various epoxide/cyclic anhydrides copolymerization and terpolymerizations of epoxide/cyclic anhydrides/CO_2_ [10,11]. This was followed in 2011 by another two catalysts for epoxide/anhydride copolymerization, *N*,*N*′-bis(salicylidene)-cyclohexanediamine chromium-(III) chloride [(salcy)CrCl], which have been successfully used in epoxide/CO_2_ copolymerizations [12,13,14]. Since then, (salcy)MX complexes with similar backbones have become some of the most widely used complexes for epoxide/anhydride copolymerization, including Cr [13,14,15,16,17], Co [13,14,15,17,18,19], Al [13,15], Mn [13,20], and Fe [21] complexes. Among reported catalysts, Co and Cr-based complexes with salen-type ligands are still the most effective organometallic complexes for MAH/PO copolymerization. However, toxic Co and Cr elements are difficult to remove completely and their residuals will hinder the further application of aliphatic polyesters. Thus, it is necessary to develop metal-based complex with a low toxicity and high efficiency.

For this purpose, aluminum complexes are mainly explored owing to their low toxicity, easy preparation, and the high degree of control over the molecular weight and microstructure. In 2012, Duchateau’s group applied metal salen (salen = *N*,*N*-bis(3,5-di-tert-butylsalicylidene)diimine) chloride complexes with different diimine linkages to catalyze the MAH/PO copolymerization and found that Al-salophen complexes clearly outperformed the other three Al-complexes [15]. Recently, Wang et al. have synthesized and characterized a series of bipyridine bisphenolate Al complexes, (BpyBph)AlX (X = axial group, such as Cl, OOCCH3, OOCCF3). They assessed the catalytic activity of these complexes for MAH/PO copolymerization and investigated the impact of the ligand’s steric and electronic nature on their catalytic activity in the absence of cocatalyst. There is a negligible effect on activity (TOF = 5.9 h^−1^ vs. 5.6 h^−1^, respectively) when replacing the *para* t-Bu groups with the electron-donating –OCH3 group. However, with the bulky cumyl substituents both on the *para* and *ortho* position of the bisphenolate Al complexes, the corresponding complex was proved to be the least active (TOF = 4.0 h^−1^), possibly because of the steric bulk around the metal center and leading to hindered monomer coordination and insertion. The catalytic activity of bipyridine bisphenolate Al complexes with *para* fluorine atoms was the highest (TOF = 8.3 h^−1^), which is attributed to the increased Lewis acidity of the complex from the electron-withdrawing group facilitating the activation of PO. It is noteworthy that the ligand backbone has a significant effect on the polymerization performance for MAH/PO copolymerization. Additionally, they found the anion group in the axial group affected the catalytic activity. The catalytic activity decreased with the decrease in the leaving ability of the axial group (leaving ability: Cl^−^ > TFA^−^ > OAc^−^). As Table 1 shows, **A1**/PPNCl binary catalytic system demonstrated a remarkably higher catalytic activity (TOF = 36 h^−1^) and could produce higher *M*_n_ polyesters with a narrow and unimodal distribution (PDI = 1.28). By contrast, **B1**/PPNCl binary catalytic system demonstrated a lower activity (TOF = 6.0 h^−1^) and lower *M*_n_ unsaturated polyesters [22]. From this, it is clear that the bipyridine bisphenolate Al-complex (**A1**) provides superior results to the salen Al complex (**B1**). However, the origin of such activity discrepancies induced by the ligand backbone has remained unclear, which is fundamentally crucial for the further development of such catalyst systems.

The regression analysis technique has been successfully applied to construct a quantitative structure-property relationship (QSPR) of polymerization catalysts, which is a meaningful method to analyze the main factor governing catalytic performance on the basis of the molecular descriptor and QSPR model [23,24]. For this purpose, some data sets for QSPR analysis consisted of a known catalyst structure and the catalytic performance measured by experimental and computational approaches [25]. Sun’s group investigated the relationship between the structure of late transition metal complexes and their experimental activity in ethylene oligo/polymerization by molecular modeling and the QSAR method [24]. Our group explored the origin of the stereoselectivity in the yttrium-catalyzed polymerization of 2-vinylpyridine and the poisoning effect of a polar monomer toward Brookhart-type catalysts, by combining DFT calculations and multivariate regression analysis [26]. As for the copolymerization of anhydride and cyclohexene oxide, previous studies have focused mostly on the ROCOP of phthalic anhydride and cyclohexene oxide catalyzed by the Cr-complexes. It was revealed that the maximal rate was achieved with one equivalent of [PPN]Cl as the cocatalyst and the polymerization rate is first-order dependence on [epoxide]. The ring-opening of epoxide was the rate-determining step [27]. Tolman and co-workers reported a mechanistic study on the ROCOP of epoxides and cyclic anhydrides using a (salph)AlCl/PPNCl (PPN = bis(triphenylphosphine)iminium) catalytic pair [28]. However, there is a lack of systematic studies into the structure effect of the ligand on the copolymerization activity.

In the present work, to elucidate the relationship between the ligand backbone of Al complexes and their catalytic activity in MAH/PO copolymerization (Table 1), DFT calculations have been conducted to disclose the polymerization mechanism and the origin of activity difference. Then, some key descriptors were calculated to correlate the catalyst activity (TOF value), and a QSPR model is thus constructed. On the basis of these theoretical studies, it has been found that the higher activity of **A1** with bipyridine bisphenolate than the **B1** with a traditional salen-type ligand can be explained by the lower LUMO energy and more negatively charged coordinating oxygen atoms.

## 2. Results and Discussion

To discriminate the distinct effects of the BpyBph ligand from typical salen analogues, we comparatively explored the possible mechanism of ROCOP of MAH/PO catalyzed by **A1** or **B1** with the aid of DFT calculations in the present paper. The complexes **A1** and **B1** were simplified to **A** and **B**, respectively, in which the t-Bu substituents were simplified by H atoms. To prove the rationality of the simplified models, the key steps involving PO insertion (**INT3** → **TS2** and **INT10** → **TS4**) mediated by the original catalyst (**A1** and **B1**) were calculated for comparison. As shown in Table S1, the energy differences for the abovementioned steps are almost unchanged, in spite of the full or simplified model, suggesting a neglectable effect of the t-Bu substituents on the relative energies.

### 2.1. Chain Initiation Stage

**Figure 1:**
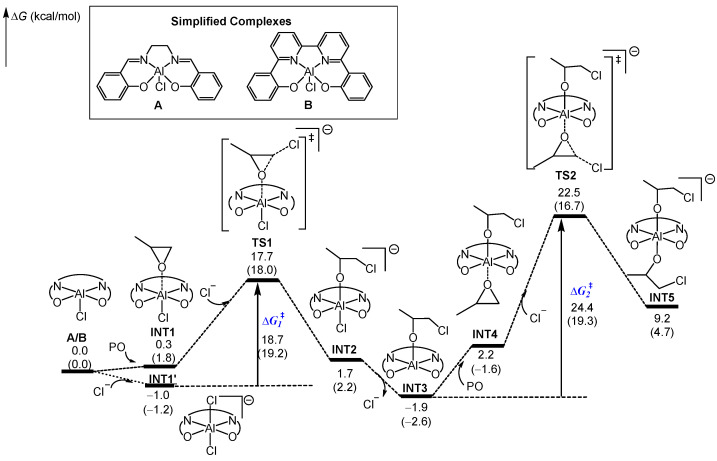
The calculated reaction routes for chain initiation mediated by **A** or **B**. The relative free energies in the **A**-catalyzed system and **B**-catalyzed system (in parentheses) are given in kcal/mol. The symbol ‘‡’ means transition state.

The computed energy profiles for the chain initiation step mediated by complexes **A** and **B** are shown in Figure 1. Upon an attack of PO to **A** (or **B**), the PO could be activated by the initial five-coordination Al complex and undergo ring-opening upon the nucleophilic attack of free Cl^−^ via the concerted **TS1**, leading to the **INT2**. Alternatively, it is possible to convert the initial aluminum complex (**A** or **B**) to 6-coordination bis(chlorinated) aluminate complex **INT1’** by its reaction with exogenous Cl^−^ derived from [PPN]Cl. Owing to the fact that the weakly nucleophilic Cl^−^ is an easier leaving group than alkoxide with a strong nucleophilicity, the resulting six-coordination aluminate species **INT2** is feasible to release the chloride anion, leaving a vacant site available for coordination of the coming PO. A coordination of PO to **INT3** generated the neutrally 6-coordinated **INT4** which could be converted to the bis(alkoxide) Al complex **INT5** via **TS2** by the attack of another nucleophilic anion. It is noteworthy that the ring-opening of PO in the chain initiation stage proceeded at the two sides of the [ONNO-Al] plane, and the bis(alkoxide) Al complex **INT5** was the real active species that could initiate the subsequent chain propagation. As shown in Figure 1, the activation energies (∆*G*_1_^‡^) of the first PO insertion were very similar for complexes **A** and **B**; however, the ∆*G*_2_^‡^ for complex **A** (24.4 kcal/mol) is higher than that for **B** (19.3 kcal/mol), suggesting the slower chain initiation by complex **A**.

To elucidate the origin of the slower chain initiation in **A**-mediated polymerization, the distortion/interaction analysis was comparatively performed for the key TSs, **A_TS2** and **B_TS2**, respectively [29,30,31,32]. Both TSs were divided into three fragments, catalyst alkoxide (Fragment A), monomer moiety (Fragment B), and chloride ion (Fragment C) (Table 2), and these energies were evaluated through single-point calculations. The interaction energy Δ*E*_int_ was estimated by the single-point energies of TS and the three fragments. These single-point energies, together with the energies of the respective fragments in their optimal geometry, allow the estimation of the distortion energies of the three fragments, ∆*E*_dist_(A), ∆*E*_dist_(B), and ∆*E*_dist_(C). The distortion energy of the fragment is defined as the energy difference between its distorted geometry in the transition state and its optimized structure. Therefore, the energy of the transition state, ∆*E*^‡^, is ∆ *E*^‡^ = Δ*E*_int_ + ∆*E*_dist_(A) + ∆*E*_dist_(B) + ∆*E*_dist_(C).

As shown in Table 2, although the interaction energies (∆*E*_int_) of the three fragments are almost equal (−33.9 vs. −34.1 kcal/mol) in the two TSs, the disfavored items of ∆*E*_dist_(A) and ∆*E*_dist_(B) in **A_TS2** (17.9 and 20.0 kcal/mol) are much larger than in **B_TS2** (15.0 and 18.6 kcal/mol), which indicates that the larger distortion could account for the lower stability of **A_TS2**. To gain more insight, the geometries of fragment **A** are carefully compared. The change in the dihedral angle of the [ONNO] equatorial ligand plane in fragment **A** of **A_TS2** (13.3° for the fragment A of **A_TS2** relative to **B_INT3**) is much bigger than in the case of **B_TS2** (6.3° for the fragment A of **B_TS2** relative to **B_INT3**). These geometrical changes mainly account for the larger distortion of **A_TS2** and thus its lower stability.

### 2.2. Chain Propagation Stage

**Figure 2:**
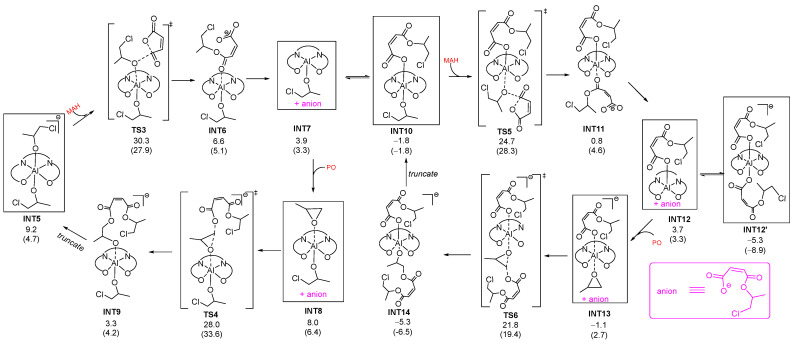
The calculated reaction routes for chain propagation mediated by **A** or **B**. The symbol "‡”denotes transition state. The pink and red ones represent anion and monomers, respectively. The relative free energies in the **A**-catalyzed system and **B**-catalyzed system (in parentheses) are given in kcal/mol. The energy reference point is the same as that in Figure 1.

To further explore the effect of the ligand backbone on the polymerization activity, the chain propagation was also calculated, as shown by the following process (Figure 2). Based on the bis(alkoxide) Al complex **INT5**, the enchainment of MAH could take place through **TS3** and yield anionic six-coordination **INT6** with alkoxide at one side and carboxylate at the other side of the ONNO-Al plane. The release of the carboxylate anion (an easier leaving group than alkoxide) resulted in five-coordination neutral **INT7** with an alkoxide and a vacant coordination site in the axial position, which could coordinate with either PO or RCOO^−^ (dissociated from **INT6**) and thus has two possible reaction pathways. One possible route is the formation of **INT8**, in which PO was activated upon coordination to **INT7**. Then, the bis(alkoxide) Al complex **INT9** was generated by the nucleophilic attack of RCOO^−^ via transition state **TS4**. **INT9** is structurally similar to the bis(alkoxide) aluminate complex **INT5**, and the repeat of the process of **INT5** to **INT9** could grow the polymer chain. The alternative route is an association of RCOO^−^ with **INT7** and the formation of the anionic six-coordination **INT10**. Upon the approach of MAH, the alkoxide in **INT10** could nucleophilically attach the carboxyl carbon of the MAH and generate the bi(carboxylate) Al complex **INT11**, which could convert to **INT12** via the release of a carboxylate anion. The RCOO^−^ anion (dissociated from **INT11**) is easily coordinated with **INT12** in another fashion and forms more stable intermediate (**INT12’**). Similar to **INT7**, PO could coordinate to **INT12** and was ring-opening under the action of a dissociated carboxylate anion from **INT11** to afford **INT14** (analogues to **INT10**). The chain propagation could occur via a similar process from **INT10** to **INT14**.

As shown in Figure 2, the free energy barrier for epoxide opening (**INT12’ → TS4**) is the highest (∆*G*_4_^‡^: 33.3 and 42.5 kcal/mol for the **A**- and **B**-catalyzed systems, respectively) among the reaction steps. Therefore, the ring-opening of PO serves as a rate-determining step of the copolymerization reaction. This is in agreement with the experimental finding that the polymerization rate is first-order in [PO] and zero-order in [MAH] [22]. Meanwhile, it was found that the overall energy barrier of the **B** system is ∆*G*_4_^‡^ = 42.5 kcal/mol, which is 9.2 kcal/mol higher than that in the **A** system. This is in line with the considerable difference in reactivity between complexes **A1** and **B1** [22].

**Figure 3:**
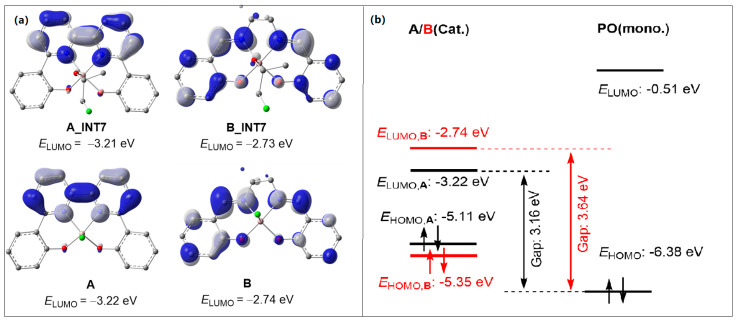
(**a**) LUMO plots (isosurface value = 0.02 a.u.) and corresponding orbital energies. The blue and grey clouds in the figure represent the positive and negative orbits. (**b**) Energy electronic levels diagrams.

Furthermore, distortion/interaction analyses for turn-over limiting TSs (**A_TS4** and **B_TS4**) were conducted as well, in order to elucidate the ligand framework effect on catalytic activity. As shown in Table 3, in the case of **A_TS4**, the total deformation energy ∆*E*_dist_ is 42.4 kcal/mol, which could be balanced out by its ∆*E*_int_ (–46.1 kcal/mol) leading to an ∆*E* of −3.7 kcal/mol. By contrast, the less negative interaction energy (−39.5 kcal/mol) in **B_TS4** could not compensate for its total deformation energy (∆*E*_dist_ = 41.2 kcal/mol), thus producing a higher ∆*E*^‡^ (1.7 kcal/mol). Therefore, the stronger interaction between three fragments could account for the greater stability of **A_TS4**. Meanwhile, it has been found that the interaction (−29.6 kcal/mol) between the fragment A and B in **A_TS4** is stronger than that in **B_TS4** (−24.8 kcal/mol) and mainly contributes to the stability of **A_TS4**. Such a discrepancy probably originates from the higher electrophilic ability of **A_INT7** compared with **B_INT7**. As expected, the calculated LUMO energies for **A_INT7** and **B_INT7** were −3.21 and −2.73 eV (Figure 3a), respectively. With the LUMO population on the ligand backbone, the LUMO energy of **A**_**INT7** was closer to the HOMO energy of PO, which resulted in an increase in the reactivity of the corresponding metal complex toward PO (Figure 3b). Essentially, these differences in LUMO energy were also observed for **A** and **B** (−3.22 vs. −2.74 eV, Figure 3a). Thus, the lower LUMO energy of **A1**, which bears the BpyBph ligand, may account for the higher activity of **A1** than the typical (salen)Al analogues (**B1**).

### 2.3. Multivariate Linear Regression Analysis

**Figure 4:**
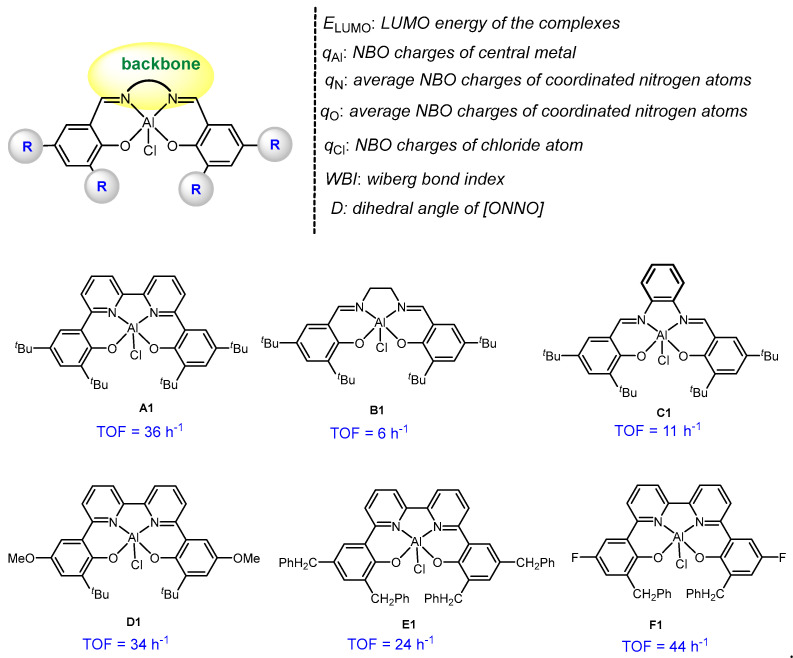
Some descriptors and TOF values (in h^−1^) for the six complexes (**A1**~**F1**).

To further validate the above ligand effect, we further explored the six complexes (**A1**~**F1**, Figure 4) that have been reported in the original paper [22] on the basis of the elucidated reaction mechanisms in the **A** and **B** systems, and constructed their quantitative structure-activity relationship through multivariate linear regression (MLR) analysis. The catalytic activity toward PO and MAH copolymerization was estimated by the turnover frequency (TOF, h^−1^) value. Seven parameters of each aluminum chloride complex were chosen to characterize the copolymerization activity of these complexes (**A1**~**F1**, Figure 4), including the LUMO energy (*E*_LUMO,_ in eV), Wiberg bond index of Al-Cl (WBI), dihedral angle of [ONNO] (*D*, in degree), the NBO charges of central metal (*q*_Al_) and coordinating heteroatoms, viz., chloride atom (*q*_Cl_), nitrogen atoms (*q*_N,_ average charge on the N atoms), and oxygen atoms (*q*_O,_ the average charge on the O atoms).

Taking the catalysts **A1**~**F1** as the training set, we constructed the catalyst activity regression model for CHO and MAH copolymerization, with the help of the stepwiselm function in Matlab to perform multivariate linear regression and remove the insignificant descriptors (*p*-value > 0.05). The TOF and parameters (seven descriptors aforementioned, viz., *E*_LUMO_, WBI, *D*, *q*_Al_, *q*_Cl_, *q*_N_, and *q*_O_) are shown in Table S2. The accuracy of the model was good, with the training determination coefficient (R^2^) of 0.98 and the ‘3-fold’ cross-validation determination coefficient (Q^2^) of 0.88. As expected, the predicted TOF values derived from the MLR model are consistent with the experimental ones.

**Figure 5:**
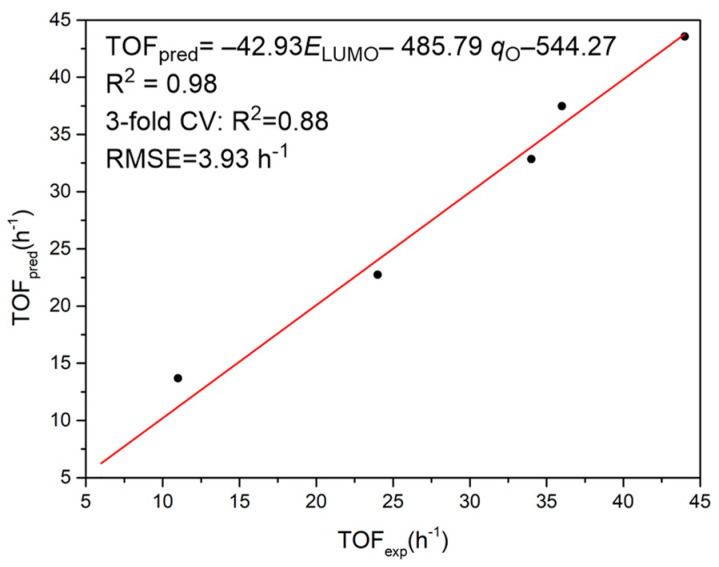
Plot of the experimental activity TOF_exp_ (h^−1^) vs. corresponding prediction values of TOF_pred_ (h^−1^) derived from the multivariate linear regression model. Black circles are six complexes (**A1**~**F1**) and two black circles (**A1** and **D1**) are overlapped in the figure. The red line to the predicted TOF values has the Pearson correlation coefficient R = 0.98.

In the regression model, only the LUMO energy (*E*_LUMO_) and the average NBO charges of oxygen atoms (*q*_O_) were retained (Figure 5). According to the negative values of *E*_LUMO_ and *q*_O_, a more negative *E*_LUMO_ and a more negative *q*_O_ could lead to a higher TOF value. In addition, it has been found that electron-withdrawing groups or conjugated backbone endowed a higher activity (higher TOF value) of the corresponding metal complexes. This demonstrates that the electron-withdrawing group or conjugated backbone is beneficial to decreasing such a LUMO energy or the average NBO charges of oxygen atoms. Such an electronic effect therefore increases the activity of the corresponding metal complex toward PO/MAH copolymerization. This strategy was promising to utilize in the future design of metal-based catalysts and expand their application by advancing catalytic properties.

## 3. Methods

The M06L functional was used for geometrical optimization and subsequent frequency calculations without any symmetry or geometrical constraints [33]. Frequency calculations were performed to ensure that the structures found were stationary points (no imaginary frequencies for minima and one imaginary frequency for transition-state structures). Intrinsic reaction coordinate (IRC) calculations were also carried out to investigate whether each of the transition structures actually connected the reactant and product [34,35]. The optimization of the transition state was carried out using the Berny algorithm [36]. In these calculations, the double-ζ 6-31+G(d,p) basis set was used for all atoms [37,38]. Such basis sets are referred to BSI. To obtain more accurate energies, single-point energy calculations were performed at the level of M06-2X/BSII together with the SMD model for considering the solvation effect of THF [39], which was used for modeling epoxide as the solvent [28,40]. In the BSII, the 6-311+G(d,p) basis set was used for all of the atoms. To justify the use of the above XC-functional and basis sets, the key intermediates and transition states were calculated at the level of SMD(THF)/M06-2X/6-311++G(2d,p)//B3LYP-D3/6-311+G** [41,42,43,44]. The comparative data with different computational methods are collected in Table S3. The three-dimensional images of the optimized structures were prepared using CYLview [45]. All the calculations were performed by the Gaussian 16 program [46]. As indicated by previous calculations for the separated and anion-paired [PPN]^+^ cations, the [PPN]^+^ cation could be viewed as a noncoordinating and separated counterion owing to its large bulk [28].

## 4. Conclusions

The ligand effect in the copolymerization of maleic anhydride (MAH) and epoxides (PO) catalyzed by Al-based complexes has been investigated by DFT calculations. Having achieved an agreement in activity discrepancy between BpyBph and salen-ligated Al complexes, it is found that the BpyBph ligand endows its Al complex with a lower LUMO energy than its salen counterpart, thus leading to the higher catalytic activity. Inspired by this ligand effect, a quantitative structure-activity relationship has been constructed via multivariate linear regression (MLR) analysis. The trained MLR model features an R^2^ value of 0.98 and a ‘3-fold’ cross-validation determination coefficient (Q^2^) of 0.88. Strong correlations were found between the catalyst activity of aluminum chloride complexes and their LUMO energies (*E*_LUMO_), as well as the average NBO charge of coordinating oxygen atoms (*q*_O_). When the E_LUMO_ is lower and the ligation oxygen atoms are more negatively charged (more negative *q*_O_), the Al complexes have a higher catalytic activity and lead to more rapid PO/MAH copolymerization. This is in agreement with the experimental results in which the complexes (**F1**) with an electron-withdrawing group or conjugated backbone demonstrate the highest TOF value, compared with other aluminum analogues (**A1**~**E1**). The mechanistic insight gained in this study could help to better interpret the catalytic activity and contribute to the rational design of novel metal catalysts.

Catalysts are the core competence of determining the industrial production efficiency and advanced product development of polyester. The development of traditional catalysts relies on chance encounters, chemical intuition, and large-scale experimental behavior. Meanwhile, the measurement of catalytic performance is high cost and needs a lot of resources as well. In order to meet the requirements of current social and economic development, it is urgent to change the outmoded development pattern to a predictable and designable model. With the rapid evolution of high-performance computing technology, the era of artificial intelligence is inexorably surging. Relying on the rapid development of different algorithms and computer hardware, it is the right time to harvest the potential of machine learning in the field of catalysis across academy and industry. Despite a substantial number of successful applications of machine learning, this exciting topic is still largely in its nascent stage and it is believed that machine learning will play an increasingly important role in accelerating the development of various kinds of functional materials in the foreseeable future. Herein, we will focus on the development of novel polyester and polyolefin catalysts by using machine-learning methods combined with essential DFT calculations, and provide insight into the underlying mechanism of the relationship between the micro-structure of the catalyst and its polymerization performance by constructing the prediction models of catalytical activity and selectivity at the molecular and electronic level in the near future. This may provide a catalyst design guideline for developing high-performance polymerization catalysts and accelerate new high-performance catalyst development and materials discovery.

## Figures and Tables

**Table 1:** ROAC of MAH/PO by Al complexes bearing different ligand backbones in the presence of PPNCl.

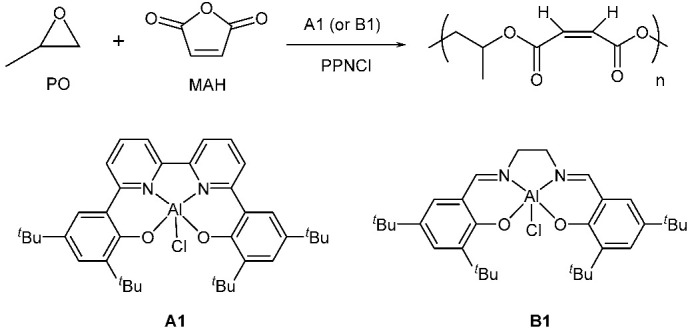							
Catalyst	*t* (h)	Conv. (%)	TOF (h^−1^)	Ester Linkage (%)	*M*_n,thero_ (kDa)	*M*_n,sec_ (kDa)	PDI
**A1**/PPNCl	2	71	36	>99	5.6	4.6	1.28
**B1**/PPNCl	2	12	6.0	>99	0.9	n.d.	n.d.

**Table 2:** Distortion/interaction analysis of (**a**) **A_TS2** and (**b**) **B_TS2**. Energies are given in kcal/mol. Green circle denotes catalyst fragment. Blue circle denotes monomer fragment. Pink circle denotes anion. The symbol ‘‡’ means transition state.

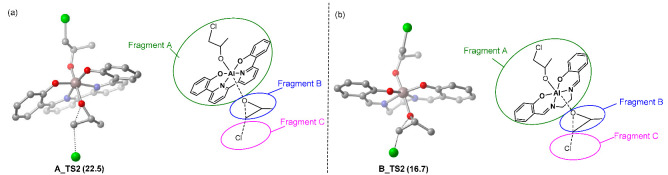							
TS2	Δ*G*^‡^	Δ*E*int(A-B-C)	Δ*E*dist(A)	Δ*E*dist(B)	Δ*E*dist(C)	Δ*E*dist	Δ*E*^‡^
**A_TS2**	24.4	−33.9	17.9	20.0	0.0	37.9	3.9
**B_TS2**	19.3	−34.1	15.0	18.6	0.0	33.6	−0.5

**Table 3:** Distortion/interaction analysis of (**a**) **A_TS4** and (**b**) **B_TS4**. Energies are given in kcal/mol. Green circle denotes catalyst fragment. Blue circle denotes monomer fragment. Pink circle denotes anion. The symbol ‘‡’ means transition state.

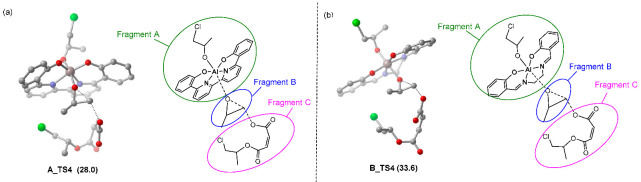							
TS4	Δ*G*^‡^	Δ*E*int(A-B-C)	Δ*E*dist(A)	Δ*E*dist(B)	Δ*E*dist(C)	Δ*E*dist	Δ*E*^‡^
**A_TS** **4**	33.3	−46.1	18.0	23.3	1.0	42.4	−3.7
**B_TS** **4**	42.5	−39.5	14.8	26.1	0.2	41.2	1.7

## Data Availability

The data presented in this study are available on request from the corresponding author.

## Supplementary Materials

The following supporting information can be downloaded at: https://www.mdpi.com/article/10.3390/molecules28217279/s1, Table S1. The computed free energy barrier for the key steps with full and simplified modes (Energy in kcal/mol); Table S2. Computed descriptors and TOF values (in h^−1^) for the six complexes (**A1**~**F1**). Table S3. The comparative data with different computational methods. The Cartesians of all explored structures as well as a list of the energies and imaginary frequencies were present in the Supplementary Materials.

## Sample Availability

Samples of the compounds are not available from the authors.
