# Supplementary material for: Mechanistic Studies on Aluminum-Catalyzed Ring-Opening Alternating Copolymerization of Maleic Anhydride with Epoxides: Ligand Effects and Quantitative Structure-Activity Relationship Model
Source: Molecules. 2023 Oct 26;28(21):7279. doi: 10.3390/molecules28217279 (PMC10649423; doi:10.3390/molecules28217279)
Supplement: Supplementary file 1 [file molecules-28-07279-s001.zip › molecules-2644098-supplementary.pdf]

Supporting information

To prove the rationality of simplified models, the key steps involving PO insertion (**INT3→TS2** and **INT10→TS4**) mediated by original catalyst (**A1** and **B1, Figure 1**) were calculated for comparison. As shown in the Table S1, that energy differences for the abovementioned steps are almost unchanged, in spite of full or simplified model. The *ortho* and *para* ^{t}Bu groups on BpyBph ligand and salen ligand have negligible effect on the reactivity difference. Thus, the simplified models used in this work is reasonable.

Table S1. The computed free energy barrier for the key steps with full and simplified modes (Energy in kcal/mol)

Original catalyst			simplified models		
System	$\Delta G^{\ddagger}_{(INT3\to TS2)}$	$\Delta G^{\ddagger}_{(INT10\to TS4)}$	System	$\Delta G^{\ddagger}_{(INT3\to TS2)}$	$\Delta G^{\ddagger}_{(INT10\to TS4)}$
**A1**	24.1	30.6	**A**	24.4	29.8
**B1**	18.5	33.2	**B**	19.3	35.4
$\Delta\Delta G^{\ddagger}$	5.6	-2.6	$\Delta\Delta G^{\ddagger}$	5.1	-5.6

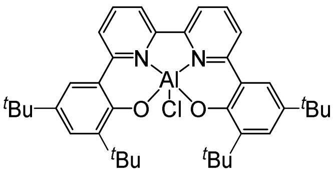

A1

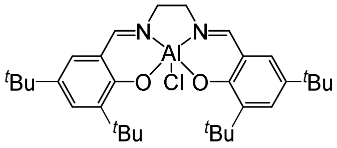

B1

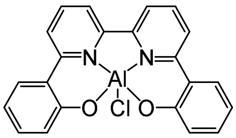

A

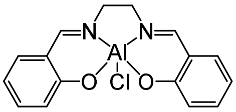

B

Table S2. Computed descriptors and TOF values (in h^{-1}) for the six complexes (**A1–F1**).

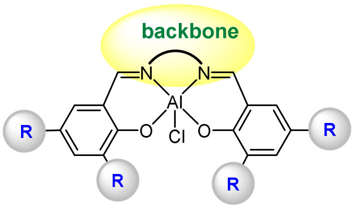

E$_{LUMO}$: *LUMO energy of the complexes*
q$_{Al}$: *NBO charges of central metal*
q$_{N}$: *average NBO charges of coordinated nitrogen atoms*
q$_{O}$: *average NBO charges of coordinated nitrogen atoms*
q$_{Cl}$: *NBO charges of chloride atom*
WBI: wiberg bond index
D: dihedral angle of [ONNO]

Cat.	backbone	R	TOF (h^{-1})	*E*$_{LUMO}$	q_{Al}	q_N	q_O	q_{Cl}	*WBI*	*D*
**A1**	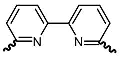	^{t}Bu	36	–3.18	1.791	–0.630	–0.9165	–0.549	0.7053	–8.9915

**B1**	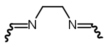	^{t}Bu	6	–2.66	1.787	–0.664	–0.895	–0.561	0.681	15.5077
**C1**	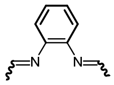	^{t}Bu	11	–2.96	1.779	–0.662	–0.887	–0.553	0.6975	6.90562
**D1**	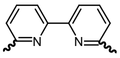	OMe	34	–3.14	1.795	–0.6105	–0.9105	–0.549	0.7052	–9.0099
**E1**	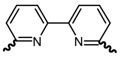	CH_2Ph	24	–3.21	1.799	–0.633	–0.8835	–0.548	0.7034	–8.1029
**F1**	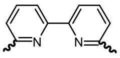	F	44	–3.39	1.796	–0.6125	–0.9105	–0.544	0.7089	–8.9037

Table S3. The comparative data with different computational methods.

free energy barrier ($\Delta G_4^{\ddagger}$, kcal/mol) for epoxide opening	*Method1:* SMD(THF)/M06-2X/6-311+G(d,p)//M06-L/6-31+G**	*Method 2:* SMD(THF)/M06-2X/6-311++G(2d, p)// B3LYP-D3/6-311+G**
***A**-catalyzed system*	33.3	*32.9*
***B**-catalyzed system*	42.5	*42.2*

As shown in the Table S3, the energy differences for the rate-determining steps are almost unchanged (9.2 vs 9.3 kcal/mol)

The Cartesians of all explored structures as well as a list of the energies and imaginary frequencies were present as follows.

10
PO E = -193.0972738

C	0.151012000	0.041337000	0.487973000
C	1.499967000	-0.099666000	-0.146184000
O	-0.823185000	0.781135000	-0.247381000
H	0.147997000	0.271815000	1.556075000
H	2.078826000	-0.891543000	0.338831000
C	-1.035105000	-0.612149000	-0.055956000
H	-1.870059000	-0.871342000	0.594918000

H	-0.939938000	-1.221934000	-0.955651000
H	2.070714000	0.829521000	-0.062795000
H	1.402694000	-0.342726000	-1.207331000

9

MAH E = -379.2701895

C	-0.000364000	-0.154751000	1.128813000
C	0.001718000	1.252486000	0.667978000
C	0.001718000	1.252486000	-0.667978000
C	-0.000364000	-0.154751000	-1.128813000
O	-0.002083000	-0.968887000	0.000000000
O	-0.000364000	-0.599424000	-2.242259000
O	-0.000364000	-0.599424000	2.242259000
H	0.003120000	2.084525000	1.359095000
H	0.003120000	2.084525000	-1.359095000

1

CL- E = -460.2467384

Cl	0.000000000	0.000000000	0.000000000

42

A E = -1809.5210726

Cl	-0.000848000	-0.780235000	2.138577000
Al	-0.000076000	-0.514909000	0.002920000
O	1.266749000	-1.538728000	-0.753231000
O	-1.266564000	-1.537526000	-0.755519000
N	1.299810000	1.055318000	-0.131811000
N	-1.299948000	1.055415000	-0.131272000
C	2.546608000	-1.555431000	-0.471640000
C	-2.546249000	-1.555070000	-0.472828000
C	-3.242498000	-2.777624000	-0.592816000
C	3.243161000	-2.777889000	-0.591121000
C	0.734948000	2.285804000	-0.164645000
C	2.657857000	0.923772000	-0.107991000
C	-0.735137000	2.285846000	-0.164460000
C	4.580426000	-2.875172000	-0.264403000
C	-2.657934000	0.923736000	-0.107326000
C	4.635533000	-0.533844000	0.262817000
C	3.272129000	-0.391827000	-0.081203000
C	5.285535000	-1.748825000	0.186630000
C	1.486991000	3.451228000	-0.182597000
C	-3.271902000	-0.392022000	-0.080993000
C	-1.487267000	3.451236000	-0.182501000
C	3.454660000	2.089130000	-0.151348000
C	2.875659000	3.339219000	-0.180830000
C	-4.579628000	-2.875471000	-0.265597000
C	-2.875924000	3.339121000	-0.180143000

C	-4.635098000	-0.534583000	0.263498000
C	-3.454864000	2.088986000	-0.150322000
C	-5.284852000	-1.749670000	0.186565000
H	2.672510000	-3.640409000	-0.919624000
H	5.083597000	-3.835690000	-0.339939000
H	6.327333000	-1.829484000	0.479253000
H	5.185723000	0.326796000	0.631653000
H	-2.671837000	-3.639776000	-0.922267000
H	-5.082602000	-3.836049000	-0.341712000
H	-6.326544000	-1.830810000	0.479436000
H	-5.185298000	0.325697000	0.633177000
H	-4.532050000	1.992388000	-0.189479000
H	-3.497386000	4.228792000	-0.215290000
H	-1.010012000	4.422910000	-0.205687000
H	1.009638000	4.422866000	-0.205208000
H	3.497056000	4.228932000	-0.216069000
H	4.531835000	1.992650000	-0.190892000

52

A_INT1’ E = -2002.6403133

Cl	0.058078000	-0.614228000	-2.402414000
Al	0.038364000	-0.411085000	-0.215897000
O	-1.288230000	-1.540293000	0.282620000
O	1.362020000	-1.498194000	0.341659000
N	-1.287629000	1.136694000	-0.199908000
N	1.327385000	1.161354000	-0.194549000
C	-2.527413000	-1.525701000	-0.148710000
C	2.617702000	-1.466904000	-0.026981000
C	3.342765000	-2.679681000	-0.021344000
C	-3.214751000	-2.753945000	-0.257243000
C	-0.728165000	2.359423000	-0.060732000
C	-2.637977000	0.993094000	-0.261850000
C	0.744986000	2.376041000	-0.087066000
C	-4.518860000	-2.813218000	-0.708056000
C	2.679012000	1.043403000	-0.275196000
C	-4.558009000	-0.424729000	-0.931145000
C	-3.231200000	-0.323690000	-0.459566000
C	-5.199496000	-1.640880000	-1.065229000
C	-1.494605000	3.511235000	0.066756000
C	3.301887000	-0.268066000	-0.393728000
C	1.483560000	3.550671000	-0.045398000
C	-3.450077000	2.136957000	-0.107988000
C	-2.881283000	3.383776000	0.056259000
C	4.660665000	-2.735061000	-0.428428000
C	2.872333000	3.455076000	-0.098406000

C	4.643562000	-0.368478000	-0.822873000
C	3.465439000	2.214765000	-0.206129000
C	5.319466000	-1.572570000	-0.853728000
H	-2.662474000	-3.651986000	0.001300000
H	-5.011244000	-3.777701000	-0.803017000
H	-6.211817000	-1.684492000	-1.453650000
H	-5.082310000	0.476545000	-1.236242000
H	2.804667000	-3.571792000	0.284503000
H	5.181417000	-3.689322000	-0.437560000
H	6.343178000	-1.615125000	-1.211260000
H	5.156567000	0.521536000	-1.175598000
H	4.544696000	2.132069000	-0.210688000
H	3.484932000	4.350256000	-0.046255000
H	0.996898000	4.515498000	0.027145000
H	-1.028251000	4.482023000	0.181449000
H	-3.511972000	4.258998000	0.181446000
H	-4.526990000	2.026031000	-0.091519000
C	0.068449000	-0.547373000	3.232689000
C	-1.171096000	0.122113000	2.842115000
O	-0.003172000	0.236095000	2.018793000
H	0.680396000	-0.043395000	3.982897000
H	-1.474740000	1.056535000	3.313399000
H	-1.965949000	-0.477486000	2.399394000
C	0.253968000	-2.024465000	3.111902000
H	1.298915000	-2.273511000	2.914231000
H	-0.043310000	-2.501691000	4.051424000
H	-0.347079000	-2.424966000	2.293374000

43

A_INT1 E = -2269.8174508

Cl	0.000466000	-0.854806000	2.001087000
Al	0.000068000	-0.490247000	-0.300885000
O	1.359712000	-1.653367000	-0.624616000
O	-1.359719000	-1.653161000	-0.624276000
N	1.318269000	1.040205000	0.058325000
N	-1.317829000	1.040305000	0.059335000
C	2.599201000	-1.606924000	-0.261889000
C	-2.599519000	-1.606307000	-0.262813000
C	-3.342696000	-2.817631000	-0.297559000
C	3.341545000	-2.818833000	-0.293470000
C	0.738082000	2.256243000	0.106596000
C	2.666844000	0.907955000	0.114103000
C	-0.737458000	2.256263000	0.107946000
C	4.658869000	-2.883155000	0.104621000
C	-2.666362000	0.908148000	0.116875000

C	4.629741000	-0.529768000	0.562413000
C	3.286886000	-0.413689000	0.141627000
C	5.317481000	-1.728852000	0.556862000
C	1.482932000	3.428439000	0.181321000
C	-3.286750000	-0.413378000	0.142463000
C	-1.482024000	3.428451000	0.185611000
C	3.459291000	2.077709000	0.132680000
C	2.870251000	3.324843000	0.169220000
C	-4.660300000	-2.881955000	0.099519000
C	-2.869371000	3.325019000	0.176164000
C	-4.629857000	-0.529636000	0.562480000
C	-3.458577000	2.078025000	0.138721000
C	-5.318347000	-1.728226000	0.554050000
H	2.803912000	-3.701497000	-0.626180000
H	5.182540000	-3.837277000	0.084358000
H	6.343661000	-1.773683000	0.910317000
H	5.140784000	0.350278000	0.943510000
H	-2.805440000	-3.699866000	-0.632024000
H	-5.184649000	-3.835626000	0.076642000
H	-6.344746000	-1.773057000	0.906851000
H	-5.140691000	0.349841000	0.945096000
H	-4.537195000	1.994313000	0.098201000
H	-3.486181000	4.219933000	0.190500000
H	-0.998619000	4.396776000	0.229171000
H	0.999698000	4.396877000	0.224281000
H	3.487096000	4.219767000	0.180808000
H	4.537830000	1.993664000	0.090250000
Cl	-0.000529000	0.311571000	-2.516852000

53

A_TS1 E = -2462.9054499 Imaginary Frequency = 418.1279

Cl	0.425085000	-0.832822000	-2.495585000
Al	0.185288000	0.166688000	-0.449655000
O	1.469201000	1.427487000	-0.706436000
O	-1.261163000	1.188061000	-0.874323000
N	1.559343000	-1.140660000	0.327213000
N	-1.039761000	-1.376335000	0.086373000
C	2.738572000	1.263994000	-0.917460000
C	-2.382053000	0.783565000	-1.400453000
C	-3.105903000	1.688968000	-2.213510000
C	3.441078000	2.302995000	-1.581270000
C	1.010322000	-2.158703000	1.021490000
C	2.901627000	-0.951997000	0.308703000
C	-0.451885000	-2.301106000	0.873772000
C	4.785350000	2.207264000	-1.870167000

C	-2.352264000	-1.470699000	-0.243996000
C	4.859032000	0.053341000	-0.819392000
C	3.488059000	0.119404000	-0.487745000
C	5.509739000	1.062411000	-1.503004000
C	1.778081000	-3.039786000	1.773905000
C	-2.953394000	-0.507167000	-1.158641000
C	-1.170263000	-3.346990000	1.442960000
C	3.714236000	-1.803415000	1.091474000
C	3.155406000	-2.836086000	1.816053000
C	-4.295125000	1.334415000	-2.815397000
C	-2.535538000	-3.425004000	1.178250000
C	-4.164098000	-0.836870000	-1.804893000
C	-3.122348000	-2.502374000	0.335511000
C	-4.830147000	0.051017000	-2.627851000
H	2.857367000	3.170561000	-1.872991000
H	5.280573000	3.017835000	-2.400902000
H	6.560179000	0.961708000	-1.759497000
H	5.420443000	-0.841254000	-0.562420000
H	-2.666461000	2.671052000	-2.356735000
H	-4.811222000	2.051465000	-3.450199000
H	-5.747957000	-0.247214000	-3.125791000
H	-4.571576000	-1.837247000	-1.683822000
H	-4.187370000	-2.544663000	0.143754000
H	-3.136621000	-4.208026000	1.632509000
H	-0.684213000	-4.080336000	2.075273000
H	1.319361000	-3.852802000	2.323926000
H	3.785854000	-3.480676000	2.422576000
H	4.780121000	-1.620117000	1.146430000
C	-0.820930000	1.046048000	2.454789000
C	-2.259262000	0.666005000	2.268873000
O	0.145403000	0.710948000	1.450711000
H	-0.443181000	0.782893000	3.449240000
H	-2.846230000	1.250240000	2.984413000
C	-0.336189000	2.300618000	1.930725000
H	0.560064000	2.754241000	2.322150000
H	-0.820678000	2.728505000	1.063556000
Cl	-1.607399000	3.976874000	3.413560000
H	-2.426076000	-0.401366000	2.452864000
H	-2.608998000	0.917281000	1.264521000

53

A_INT2 E = -2462.93804

Cl	-0.379882000	0.621109000	-2.475856000
Al	-0.163799000	-0.197273000	-0.274890000
O	-1.453801000	-1.471520000	-0.552547000

O	1.302540000	-1.218930000	-0.702655000
N	-1.564140000	1.209275000	0.239904000
N	1.050907000	1.440984000	0.029832000
C	-2.714915000	-1.321465000	-0.791241000
C	2.427619000	-0.842421000	-1.232093000
C	3.171063000	-1.796912000	-1.972219000
C	-3.416423000	-2.421106000	-1.358283000
C	-1.030840000	2.300614000	0.822227000
C	-2.903719000	1.019948000	0.198160000
C	0.437578000	2.428781000	0.710095000
C	-4.754255000	-2.354339000	-1.680206000
C	2.373159000	1.499799000	-0.247958000
C	-4.833796000	-0.103993000	-0.858468000
C	-3.470535000	-0.135596000	-0.492092000
C	-5.479699000	-1.175427000	-1.446284000
C	-1.819392000	3.271895000	1.430626000
C	2.997288000	0.463006000	-1.067419000
C	1.139671000	3.520262000	1.212722000
C	-3.737884000	1.957799000	0.849914000
C	-3.197243000	3.072946000	1.457070000
C	4.375718000	-1.485496000	-2.568296000
C	2.516868000	3.564544000	1.010512000
C	4.222665000	0.745800000	-1.705108000
C	3.132544000	2.565970000	0.281351000
C	4.910721000	-0.194497000	-2.450786000
H	-2.830087000	-3.314067000	-1.553706000
H	-5.242724000	-3.213939000	-2.135713000
H	-6.524435000	-1.096115000	-1.732956000
H	-5.396249000	0.814020000	-0.708830000
H	2.729924000	-2.784631000	-2.067783000
H	4.902372000	-2.243085000	-3.145622000
H	5.840521000	0.071261000	-2.945496000
H	4.627145000	1.753051000	-1.641622000
H	4.206535000	2.580638000	0.139256000
H	3.104819000	4.379466000	1.424990000
H	0.634033000	4.308510000	1.758058000
H	-1.376121000	4.149878000	1.885313000
H	-3.844299000	3.787886000	1.959077000
H	-4.805535000	1.782859000	0.897883000
C	0.693028000	-1.177968000	2.395695000
C	2.140345000	-0.693021000	2.426447000
O	-0.119508000	-0.445616000	1.556816000
H	0.300869000	-1.106537000	3.431650000
H	2.732721000	-1.240190000	3.168442000

C	0.579168000	-2.643497000	1.984370000
H	-0.469301000	-2.939962000	1.937428000
H	1.056265000	-2.824597000	1.018865000
Cl	1.374509000	-3.780439000	3.181869000
H	2.164573000	0.372797000	2.680607000
H	2.616081000	-0.821128000	1.448023000

52

A_INT3 E = -2002.6408085

Al	-0.190047000	-0.113260000	-0.274256000
O	-1.303681000	-0.896138000	-1.448263000
O	1.214159000	-1.004439000	-0.960225000
N	-1.620800000	1.310481000	-0.002389000
N	0.966304000	1.571885000	-0.172099000
C	-2.594743000	-1.085695000	-1.301769000
C	2.436930000	-0.704912000	-1.299774000
C	3.213946000	-1.703165000	-1.931558000
C	-3.168018000	-2.225909000	-1.906647000
C	-1.148572000	2.494176000	0.440766000
C	-2.958127000	1.077588000	-0.073956000
C	0.318169000	2.610461000	0.418188000
C	-4.507430000	-2.523776000	-1.757034000
C	2.323503000	1.618309000	-0.327996000
C	-4.808389000	-0.542289000	-0.436829000
C	-3.447290000	-0.193137000	-0.584261000
C	-5.338850000	-1.685846000	-0.999356000
C	-1.985453000	3.522095000	0.850699000
C	3.032350000	0.565275000	-1.038649000
C	0.984306000	3.705051000	0.946459000
C	-3.844723000	2.107566000	0.312081000
C	-3.361613000	3.311320000	0.780185000
C	4.508928000	-1.456630000	-2.336143000
C	2.374647000	3.733733000	0.863654000
C	4.356658000	0.783692000	-1.486222000
C	3.034584000	2.707555000	0.224289000
C	5.087852000	-0.195290000	-2.127183000
H	-2.501307000	-2.870800000	-2.470174000
H	-4.913344000	-3.421693000	-2.215838000
H	-6.385297000	-1.931861000	-0.851147000
H	-5.459995000	0.088736000	0.160001000
H	2.739721000	-2.665891000	-2.094146000
H	5.077468000	-2.240749000	-2.829333000
H	6.095544000	0.014725000	-2.470397000
H	4.810460000	1.761477000	-1.358332000
H	4.115096000	2.716220000	0.172167000

H	2.934278000	4.554819000	1.301414000
H	0.439118000	4.504396000	1.432836000
H	-1.585979000	4.468706000	1.193385000
H	-4.050428000	4.098046000	1.072927000
H	-4.912350000	1.959502000	0.214263000
C	0.453312000	-1.370629000	2.226630000
C	1.820248000	-0.724859000	2.391207000
O	-0.383467000	-0.633980000	1.382585000
H	-0.026549000	-1.426852000	3.218352000
H	2.432033000	-1.260002000	3.123307000
C	0.536903000	-2.789789000	1.676345000
H	-0.466147000	-3.190838000	1.519162000
H	1.089537000	-2.812956000	0.733011000
Cl	1.377574000	-3.916035000	2.813983000
H	1.705478000	0.311362000	2.727027000
H	2.365136000	-0.720843000	1.439021000

62

A_INT4 E = -2195.7540886

Al	-0.085595000	0.412211000	0.039013000
O	-1.308498000	1.215913000	1.091227000
O	1.324965000	0.947233000	1.029295000
N	-1.554235000	-0.692379000	-0.862442000
N	1.005068000	-1.155479000	-0.681843000
C	-2.605226000	1.130056000	1.179603000
C	2.390789000	0.317080000	1.440757000
C	3.097552000	0.854629000	2.541746000
C	-3.236947000	1.861576000	2.215559000
C	-1.047300000	-1.467291000	-1.848459000
C	-2.883242000	-0.420805000	-0.807173000
C	0.393663000	-1.758288000	-1.726222000
C	-4.600145000	1.810499000	2.416643000
C	2.295014000	-1.443129000	-0.370817000
C	-4.819275000	0.315284000	0.554365000
C	-3.427532000	0.348929000	0.305452000
C	-5.407946000	1.020293000	1.585151000
C	-1.838328000	-1.993843000	-2.858880000
C	2.908265000	-0.860253000	0.815411000
C	1.062523000	-2.638246000	-2.565734000
C	-3.718073000	-0.911651000	-1.837438000
C	-3.197585000	-1.684652000	-2.853610000
C	4.229551000	0.245442000	3.043336000
C	2.409295000	-2.893536000	-2.309904000
C	4.062477000	-1.461244000	1.365909000
C	3.019335000	-2.311766000	-1.217579000

C	4.716433000	-0.935151000	2.462874000
H	-2.591071000	2.459581000	2.850974000
H	-5.046259000	2.378394000	3.229321000
H	-6.478423000	0.954279000	1.750274000
H	-5.453791000	-0.314234000	-0.062136000
H	2.699260000	1.762948000	2.982922000
H	4.739000000	0.679687000	3.899941000
H	5.590590000	-1.433740000	2.868991000
H	4.433801000	-2.387488000	0.935925000
H	4.068707000	-2.494305000	-1.023112000
H	2.975229000	-3.548730000	-2.965560000
H	0.556201000	-3.111493000	-3.398245000
H	-1.411126000	-2.607503000	-3.642576000
H	-3.843427000	-2.045046000	-3.648836000
H	-4.768751000	-0.652536000	-1.845980000
C	0.931989000	2.275512000	-1.917942000
C	2.304144000	1.642088000	-2.100637000
O	-0.022088000	1.373127000	-1.448414000
H	0.595897000	2.653537000	-2.899761000
H	3.012765000	2.344939000	-2.549029000
C	0.958037000	3.461388000	-0.959442000
H	-0.050797000	3.854032000	-0.821644000
H	1.372218000	3.173319000	0.010389000
Cl	1.968006000	4.830237000	-1.582786000
H	2.226683000	0.766081000	-2.753823000
H	2.714411000	1.319058000	-1.136542000
C	-0.368500000	-2.591381000	2.178200000
C	-0.626932000	-3.574923000	1.080551000
O	-0.268215000	-1.208079000	1.813679000
H	0.395004000	-2.875641000	2.905095000
H	-0.962637000	-4.528609000	1.498672000
C	-1.376340000	-1.621541000	2.608222000
H	-1.375862000	-1.226221000	3.622520000
H	-2.339563000	-1.597540000	2.095432000
H	0.280668000	-3.769480000	0.500062000
H	-1.401434000	-3.207036000	0.400962000

63

A_TS2 E = -2656.0225657 Imaginary Frequency= 417.2632

Al	-0.183708000	0.470109000	-0.187270000
O	0.878823000	1.542609000	-1.213731000
O	-1.612920000	0.340683000	-1.337139000
N	1.253242000	0.311184000	1.262022000
N	-1.078668000	-0.877658000	1.082896000
C	2.164393000	1.703586000	-1.185610000

C	-2.383887000	-0.685083000	-1.547566000
C	-2.989039000	-0.825528000	-2.821895000
C	2.786398000	2.269562000	-2.330053000
C	0.771778000	-0.187100000	2.420482000
C	2.480533000	0.881125000	1.201628000
C	-0.541110000	-0.858868000	2.319239000
C	4.151919000	2.436752000	-2.415257000
C	-2.209415000	-1.571186000	0.822678000
C	4.400040000	1.544480000	-0.204906000
C	3.004243000	1.375250000	-0.067369000
C	4.980709000	2.055399000	-1.348983000
C	1.496369000	-0.133113000	3.603895000
C	-2.705689000	-1.661142000	-0.547955000
C	-1.150298000	-1.498950000	3.392394000
C	3.236713000	0.986971000	2.392390000
C	2.746529000	0.483190000	3.578694000
C	-3.816936000	-1.887610000	-3.124758000
C	-2.355836000	-2.160379000	3.165151000
C	-3.545123000	-2.738500000	-0.899926000
C	-2.883566000	-2.203686000	1.889546000
C	-4.093755000	-2.869800000	-2.162645000
H	2.130037000	2.534914000	-3.153504000
H	4.588239000	2.845342000	-3.324428000
H	6.060523000	2.135329000	-1.425886000
H	5.051401000	1.214713000	0.599835000
H	-2.750104000	-0.065359000	-3.559573000
H	-4.242843000	-1.969099000	-4.122642000
H	-4.715401000	-3.726613000	-2.404335000
H	-3.738692000	-3.514919000	-0.163308000
H	-3.828810000	-2.700438000	1.705608000
H	-2.877588000	-2.641816000	3.987884000
H	-0.705088000	-1.482015000	4.380130000
H	1.099895000	-0.547896000	4.522994000
H	3.330822000	0.571331000	4.490563000
H	4.194282000	1.492142000	2.373821000
C	-1.974481000	2.554648000	0.896996000
C	-3.234429000	1.732625000	1.152714000
O	-0.814953000	1.803665000	0.898785000
H	-1.908542000	3.315888000	1.700499000
H	-4.121236000	2.371004000	1.230877000
C	-2.031880000	3.313932000	-0.425433000
H	-1.098725000	3.854488000	-0.588512000
H	-2.215769000	2.638566000	-1.263750000
Cl	-3.361922000	4.567719000	-0.460859000

H	-3.126131000	1.174859000	2.089772000
H	-3.402210000	1.013689000	0.343334000
C	0.984406000	-2.451375000	-1.031843000
C	0.853408000	-3.186490000	0.272558000
O	0.625139000	-1.079762000	-1.120184000
H	0.572851000	-3.023225000	-1.872739000
H	1.455429000	-4.097451000	0.197873000
C	2.227184000	-1.757087000	-1.300414000
H	2.518088000	-1.497578000	-2.306536000
H	2.794239000	-1.363221000	-0.469369000
Cl	3.962172000	-3.594744000	-1.334953000
H	-0.184180000	-3.454340000	0.497067000
H	1.257671000	-2.592260000	1.098857000

63

A_INT5 E = -2656.051293

Al	-0.152800000	0.353364000	-0.270410000
O	0.970590000	1.436595000	-1.269132000
O	-1.660425000	0.382835000	-1.351310000
N	1.248763000	0.239677000	1.260790000
N	-1.153522000	-0.829763000	1.097567000
C	2.255376000	1.528263000	-1.216532000
C	-2.473085000	-0.610835000	-1.535962000
C	-3.122162000	-0.730070000	-2.793072000
C	2.942889000	2.004984000	-2.369152000
C	0.712004000	-0.165039000	2.429960000
C	2.476489000	0.805845000	1.217125000
C	-0.613684000	-0.814103000	2.330597000
C	4.316971000	2.094898000	-2.424296000
C	-2.302242000	-1.494106000	0.843490000
C	4.458622000	1.309637000	-0.164550000
C	3.054321000	1.212292000	-0.061032000
C	5.098203000	1.728999000	-1.316169000
C	1.389116000	-0.031468000	3.637400000
C	-2.813325000	-1.572005000	-0.523617000
C	-1.230270000	-1.439332000	3.409862000
C	3.185259000	0.995672000	2.425612000
C	2.642958000	0.576128000	3.623512000
C	-3.998889000	-1.757966000	-3.073592000
C	-2.454844000	-2.068012000	3.191261000
C	-3.702594000	-2.615109000	-0.854944000
C	-2.991522000	-2.097726000	1.918555000
C	-4.288161000	-2.728740000	-2.103262000
H	2.323914000	2.263992000	-3.223301000
H	4.797531000	2.434841000	-3.339994000

H	6.182813000	1.757839000	-1.362988000
H	5.067356000	0.998750000	0.681689000
H	-2.871918000	0.019086000	-3.538599000
H	-4.454257000	-1.822447000	-4.060188000
H	-4.948848000	-3.561508000	-2.326443000
H	-3.909573000	-3.382231000	-0.111917000
H	-3.955046000	-2.562266000	1.744307000
H	-2.985513000	-2.533170000	4.018057000
H	-0.775633000	-1.438204000	4.393814000
H	0.943478000	-0.363378000	4.567919000
H	3.185001000	0.733084000	4.552614000
H	4.140682000	1.506519000	2.409909000
C	-1.800219000	2.605551000	0.819311000
C	-3.103859000	1.870454000	1.126090000
O	-0.688422000	1.792715000	0.800422000
H	-1.670410000	3.377877000	1.607021000
H	-3.944632000	2.567237000	1.219347000
C	-1.851366000	3.348957000	-0.513138000
H	-0.891483000	3.826905000	-0.712181000
H	-2.108020000	2.677989000	-1.335001000
Cl	-3.099316000	4.694146000	-0.529396000
H	-3.002845000	1.322438000	2.070023000
H	-3.342101000	1.149774000	0.336648000
C	1.128750000	-2.351249000	-1.012016000
C	0.844517000	-3.165851000	0.249348000
O	0.430216000	-1.180287000	-1.105028000
H	0.885926000	-3.001916000	-1.879433000
H	1.388974000	-4.116592000	0.240258000
C	2.615476000	-2.011440000	-1.123266000
H	2.801248000	-1.376643000	-1.991736000
H	2.979620000	-1.507339000	-0.223604000
Cl	3.681464000	-3.486577000	-1.331034000
H	-0.226441000	-3.379244000	0.327941000
H	1.149312000	-2.609828000	1.145223000

72

A_TS3 E = -3035.3134411 Imaginary Frequency= 153.1102

Al	0.890427000	-0.604330000	-0.299735000
O	0.143839000	-1.404009000	-1.731229000
O	1.683075000	0.787463000	-1.111746000
N	-0.140787000	-1.852352000	0.942797000
N	1.234429000	0.277875000	1.508099000
C	-0.956332000	-2.071242000	-1.857846000
C	1.711310000	2.043414000	-0.759554000
C	1.731599000	3.034175000	-1.766958000

C	-1.557472000	-2.143086000	-3.141746000
C	0.327922000	-1.805075000	2.207679000
C	-1.004745000	-2.820418000	0.553219000
C	1.106388000	-0.591946000	2.531044000
C	-2.734206000	-2.830648000	-3.351124000
C	1.778180000	1.504858000	1.697040000
C	-2.792361000	-3.464614000	-1.035869000
C	-1.582664000	-2.782073000	-0.782384000
C	-3.374559000	-3.491876000	-2.289290000
C	-0.003380000	-2.767641000	3.152493000
C	1.790959000	2.461543000	0.604108000
C	1.580796000	-0.307713000	3.804532000
C	-1.348454000	-3.834310000	1.475612000
C	-0.843344000	-3.809048000	2.759484000
C	1.796077000	4.374689000	-1.450752000
C	2.206157000	0.923524000	4.009900000
C	1.858589000	3.842922000	0.889398000
C	2.295881000	1.827775000	2.972034000
C	1.856696000	4.793636000	-0.111696000
H	-1.066702000	-1.588418000	-3.935174000
H	-3.181898000	-2.838863000	-4.342803000
H	-4.320423000	-4.002775000	-2.442456000
H	-3.308267000	-3.950069000	-0.210521000
H	1.635156000	2.689301000	-2.790509000
H	1.769552000	5.114816000	-2.247182000
H	1.868741000	5.850461000	0.137457000
H	1.856843000	4.170249000	1.926820000
H	2.783829000	2.784028000	3.119203000
H	2.614837000	1.171124000	4.985898000
H	1.468161000	-1.017266000	4.615865000
H	0.377183000	-2.710098000	4.165451000
H	-1.105165000	-4.594397000	3.463392000
H	-1.995068000	-4.644430000	1.160838000
C	3.623739000	-1.624050000	-0.342718000
C	4.319588000	-0.479495000	0.386125000
O	2.277972000	-1.736761000	-0.031823000
H	4.133078000	-2.564637000	-0.054490000
H	5.395419000	-0.468987000	0.181133000
C	3.738807000	-1.502392000	-1.859350000
H	3.186266000	-2.308338000	-2.344165000
H	3.365049000	-0.537873000	-2.211151000
Cl	5.462918000	-1.644264000	-2.441230000
H	4.174898000	-0.586615000	1.466985000
H	3.903169000	0.485728000	0.077472000

C	-2.097068000	1.063721000	0.935106000
C	-1.461584000	1.938088000	2.002430000
O	-1.217365000	0.718063000	-0.131348000
H	-2.929244000	1.637160000	0.515350000
H	-2.239946000	2.370788000	2.638999000
C	-2.724654000	-0.205245000	1.497253000
H	-3.074198000	-0.852213000	0.688694000
H	-2.056056000	-0.761735000	2.156692000
Cl	-4.179367000	0.172385000	2.521476000
H	-0.912098000	2.753680000	1.525598000
H	-0.781932000	1.377009000	2.652813000
C	-3.076981000	0.900204000	-1.849212000
C	-1.668375000	1.355412000	-1.602920000
C	-3.939334000	1.915067000	-1.766568000
C	-3.218920000	3.149063000	-1.352756000
O	-3.723080000	4.251773000	-1.188578000
O	-1.929487000	2.873388000	-1.172483000
O	-0.780857000	1.204312000	-2.427537000
H	-3.270392000	-0.129494000	-2.137860000
H	-5.004523000	1.914319000	-1.968587000

72

A_INT6 E = -3035.3550774

Al	1.087555000	-0.766879000	-0.187010000
O	0.533226000	-2.090380000	-1.320722000
O	2.291840000	0.037287000	-1.295196000
N	-0.364348000	-1.282057000	1.175362000
N	1.124081000	0.839205000	1.054428000
C	-0.574648000	-2.765455000	-1.353037000
C	2.387511000	1.318792000	-1.552026000
C	2.860314000	1.716427000	-2.823255000
C	-0.926139000	-3.413784000	-2.566363000
C	-0.186610000	-0.649517000	2.353656000
C	-1.184299000	-2.351342000	1.076903000
C	0.604037000	0.590844000	2.271634000
C	-2.095871000	-4.134110000	-2.702648000
C	1.655852000	2.045167000	0.764830000
C	-2.647543000	-3.664088000	-0.418265000
C	-1.457789000	-2.931430000	-0.235471000
C	-2.982992000	-4.256567000	-1.623516000
C	-0.782296000	-1.101089000	3.526835000
C	2.070249000	2.342831000	-0.605934000
C	0.705706000	1.498585000	3.315163000
C	-1.780375000	-2.867397000	2.246564000
C	-1.569937000	-2.246375000	3.463579000

C	2.944298000	3.049361000	-3.183679000
C	1.326922000	2.718247000	3.062912000
C	2.139467000	3.688956000	-1.015107000
C	1.782638000	3.001130000	1.792159000
C	2.562154000	4.051382000	-2.283413000
H	-0.234525000	-3.296266000	-3.395537000
H	-2.334269000	-4.596293000	-3.658691000
H	-3.920472000	-4.794659000	-1.729174000
H	-3.350619000	-3.734937000	0.409145000
H	3.124146000	0.923910000	-3.517843000
H	3.289170000	3.314925000	-4.181429000
H	2.577823000	5.097618000	-2.574655000
H	1.788025000	4.460323000	-0.334491000
H	2.229128000	3.963120000	1.575346000
H	1.415346000	3.461758000	3.849483000
H	0.270164000	1.285518000	4.284755000
H	-0.624212000	-0.581743000	4.464796000
H	-2.020664000	-2.650558000	4.366377000
H	-2.383101000	-3.766076000	2.189343000
C	3.565634000	-1.798595000	0.958647000
C	4.207899000	-0.462489000	1.315383000
O	2.184972000	-1.743271000	0.865946000
H	3.838871000	-2.524380000	1.750122000
H	5.288884000	-0.564861000	1.459999000
C	4.085905000	-2.361955000	-0.360685000
H	3.563610000	-3.288416000	-0.603979000
H	3.961497000	-1.643074000	-1.173577000
Cl	5.863733000	-2.781636000	-0.297685000
H	3.768861000	-0.075636000	2.241405000
H	4.035371000	0.273718000	0.523048000
C	-3.650417000	0.782924000	-0.060248000
C	-3.834850000	0.392205000	1.384777000
O	-2.401078000	0.199400000	-0.494376000
H	-3.593258000	1.871516000	-0.160178000
H	-4.785416000	0.773149000	1.767061000
C	-4.676441000	0.204110000	-1.018296000
H	-4.452313000	0.491701000	-2.047507000
H	-4.705532000	-0.886451000	-0.949174000
Cl	-6.335236000	0.807992000	-0.659770000
H	-3.017018000	0.821637000	1.967962000
H	-3.825187000	-0.699160000	1.490627000
C	-1.744200000	2.247634000	-1.721360000
C	-1.435680000	0.971564000	-1.017352000
C	-1.602494000	3.439972000	-1.134812000

C	-1.222049000	3.514723000	0.326980000
O	-0.780617000	4.600244000	0.757734000
O	-1.376617000	2.412595000	0.943681000
O	-0.322399000	0.466864000	-1.147900000
H	-1.941893000	2.137846000	-2.787808000
H	-1.701561000	4.361234000	-1.706742000

52

A_INT7 E = -2002.6408086

Al	-0.190088000	-0.113555000	-0.273983000
O	-1.304240000	-0.897821000	-1.446622000
O	1.213838000	-1.004255000	-0.961169000
N	-1.620827000	1.310428000	-0.002597000
N	0.966341000	1.571755000	-0.171986000
C	-2.595395000	-1.086638000	-1.300303000
C	2.436531000	-0.704494000	-1.300933000
C	3.213282000	-1.702234000	-1.933821000
C	-3.169050000	-2.227102000	-1.904379000
C	-1.148521000	2.494255000	0.440213000
C	-2.958218000	1.077931000	-0.074575000
C	0.318250000	2.610148000	0.418576000
C	-4.508618000	-2.524263000	-1.754804000
C	2.323578000	1.617909000	-0.327252000
C	-4.809019000	-0.541526000	-0.436358000
C	-3.447736000	-0.193066000	-0.583830000
C	-5.339846000	-1.685346000	-0.997997000
C	-1.985276000	3.522742000	0.848938000
C	3.032232000	0.565320000	-1.038737000
C	0.984483000	3.704103000	0.948070000
C	-3.844684000	2.108565000	0.310069000
C	-3.361474000	3.312498000	0.777570000
C	4.508257000	-1.455496000	-2.338361000
C	2.374897000	3.732246000	0.866226000
C	4.356507000	0.783995000	-1.486207000
C	3.034829000	2.706342000	0.226384000
C	5.087465000	-0.194493000	-2.128220000
H	-2.502504000	-2.872752000	-2.467233000
H	-4.914830000	-3.422371000	-2.212971000
H	-6.386411000	-1.930830000	-0.849747000
H	-5.460450000	0.090285000	0.159833000
H	2.738925000	-2.664752000	-2.097240000
H	5.076582000	-2.239232000	-2.832409000
H	6.095157000	0.015658000	-2.471354000
H	4.810409000	1.761614000	-1.357326000
H	4.115387000	2.714508000	0.174926000

H	2.934582000	4.552687000	1.305129000
H	0.439311000	4.503256000	1.434790000
H	-1.585598000	4.469428000	1.191190000
H	-4.050192000	4.099736000	1.069167000
H	-4.912291000	1.960789000	0.211592000
C	0.454579000	-1.370325000	2.226729000
C	1.821910000	-0.725091000	2.390074000
O	-0.382446000	-0.633348000	1.383217000
H	-0.024583000	-1.426330000	3.218798000
H	2.434196000	-1.260415000	3.121619000
C	0.537204000	-2.789501000	1.676367000
H	-0.466107000	-3.190222000	1.520019000
H	1.089014000	-2.812828000	0.732546000
Cl	1.378472000	-3.916036000	2.813251000
H	1.707836000	0.311203000	2.725888000
H	2.365877000	-0.721365000	1.437333000

20

anion E = -1032.6666424

C	0.943081000	0.186581000	0.451244000
C	1.238773000	-0.218801000	1.873146000
O	0.206428000	1.423380000	0.493715000
H	0.344329000	-0.585602000	-0.037161000
H	1.832823000	-1.136865000	1.893738000
C	2.164097000	0.521984000	-0.388245000
H	1.867982000	0.829442000	-1.393658000
H	2.753713000	1.317686000	0.074527000
Cl	3.272081000	-0.892159000	-0.591678000
H	0.287034000	-0.398982000	2.376184000
H	1.792194000	0.570139000	2.395119000
C	-1.309956000	0.653865000	-1.310003000
C	-0.980502000	1.561092000	-0.173116000
C	-1.901120000	-0.544440000	-1.228478000
C	-2.230961000	-1.217098000	0.092749000
O	-2.888056000	-2.280601000	0.017932000
O	-1.786971000	-0.608000000	1.109081000
O	-1.620175000	2.571131000	0.038078000
H	-1.147187000	1.128213000	-2.279777000
H	-2.186549000	-1.063716000	-2.144680000

62

A_INT8 E = -2195.7540893

Al	-0.085580000	0.412198000	0.038734000
O	-1.308391000	1.216544000	1.090546000
O	1.324732000	0.947247000	1.029333000
N	-1.554300000	-0.692636000	-0.862124000

N	1.004992000	-1.155899000	-0.681329000
C	-2.605134000	1.131012000	1.178906000
C	2.390769000	0.317411000	1.440708000
C	3.097635000	0.855437000	2.541404000
C	-3.236759000	1.863280000	2.214393000
C	-1.047459000	-1.468206000	-1.847685000
C	-2.883305000	-0.420960000	-0.806963000
C	0.393507000	-1.759176000	-1.725393000
C	-4.599978000	1.812607000	2.415418000
C	2.294973000	-1.443411000	-0.370283000
C	-4.819306000	0.316323000	0.554014000
C	-3.427538000	0.349540000	0.305157000
C	-5.407887000	1.022069000	1.584344000
C	-1.838570000	-1.995400000	-2.857700000
C	2.908334000	-0.860030000	0.815637000
C	1.062299000	-2.639487000	-2.564587000
C	-3.718206000	-0.912466000	-1.836864000
C	-3.197818000	-1.686167000	-2.852547000
C	4.229839000	0.246635000	3.042978000
C	2.409100000	-2.894638000	-2.308775000
C	4.062763000	-1.460626000	1.366133000
C	3.019225000	-2.312392000	-1.216755000
C	4.716828000	-0.934057000	2.462798000
H	-2.590793000	2.461516000	2.849497000
H	-5.046032000	2.381072000	3.227729000
H	-6.478387000	0.956375000	1.749445000
H	-5.453930000	-0.313429000	-0.062132000
H	2.699233000	1.763817000	2.982357000
H	4.739367000	0.681241000	3.899353000
H	5.591154000	-1.432355000	2.868908000
H	4.434184000	-2.386944000	0.936398000
H	4.068621000	-2.494822000	-1.022327000
H	2.974992000	-3.550086000	-2.964213000
H	0.555914000	-3.113083000	-3.396859000
H	-1.411446000	-2.609616000	-3.641002000
H	-3.843726000	-2.047078000	-3.647484000
H	-4.768874000	-0.653321000	-1.845525000
C	0.932465000	2.274669000	-1.918735000
C	2.304606000	1.641084000	-2.100944000
O	-0.021744000	1.372514000	-1.449029000
H	0.596562000	2.652361000	-2.900744000
H	3.013372000	2.343710000	-2.549460000
C	0.958419000	3.460864000	-0.960630000
H	-0.050406000	3.853652000	-0.823180000

H	1.372340000	3.173051000	0.009393000
Cl	1.968673000	4.829403000	-1.584172000
H	2.227202000	0.764832000	-2.753807000
H	2.714664000	1.318389000	-1.136645000
C	-0.369454000	-2.590940000	2.179271000
C	-0.627740000	-3.574764000	1.081837000
O	-0.268530000	-1.207810000	1.814321000
H	0.393647000	-2.875227000	2.906580000
H	-0.963672000	-4.528291000	1.500135000
C	-1.377134000	-1.620615000	2.608549000
H	-1.376932000	-1.224958000	3.622717000
H	-2.340141000	-1.596461000	2.095363000
H	0.279982000	-3.769586000	0.501627000
H	-1.402026000	-3.206974000	0.401947000

82

A_TS4 E = -3228.4439556 Imaginary Frequency= 432.4393

Al	1.802484000	0.162951000	0.171528000
O	2.743946000	1.474745000	1.040949000
O	2.508038000	-1.306296000	1.008287000
N	0.689898000	1.553146000	-0.860194000
N	0.476897000	-1.061279000	-0.871097000
C	2.359613000	2.685506000	1.305983000
C	1.965447000	-2.463878000	1.252995000
C	2.450780000	-3.212547000	2.353945000
C	2.934501000	3.347780000	2.421827000
C	0.076025000	1.035867000	-1.943927000
C	0.821986000	2.889429000	-0.705583000
C	-0.062986000	-0.435582000	-1.940348000
C	2.572730000	4.630399000	2.779698000
C	0.386236000	-2.404268000	-0.737136000
C	1.070112000	4.731215000	0.915547000
C	1.419458000	3.425452000	0.513255000
C	1.618117000	5.336123000	2.031819000
C	-0.407902000	1.840091000	-2.968325000
C	0.930286000	-3.053959000	0.454143000
C	-0.720448000	-1.133785000	-2.946373000
C	0.378241000	3.743204000	-1.739572000
C	-0.224212000	3.217144000	-2.863746000
C	1.918572000	-4.439544000	2.696329000
C	-0.782124000	-2.523035000	-2.853341000
C	0.406083000	-4.305505000	0.842366000
C	-0.232819000	-3.158547000	-1.757822000
C	0.873544000	-4.995626000	1.945222000
H	3.659724000	2.782674000	2.999777000

H	3.021245000	5.089143000	3.658629000
H	1.302206000	6.331862000	2.327934000
H	0.307666000	5.264659000	0.351311000
H	3.248696000	-2.759586000	2.934737000
H	2.303557000	-4.967637000	3.565956000
H	0.423388000	-5.941102000	2.230650000
H	-0.427363000	-4.722529000	0.281700000
H	-0.251897000	-4.239592000	-1.687686000
H	-1.259794000	-3.103098000	-3.638349000
H	-1.161436000	-0.615219000	-3.789930000
H	-0.901039000	1.410834000	-3.832932000
H	-0.561138000	3.874416000	-3.660601000
H	0.542142000	4.811057000	-1.654870000
C	3.898310000	-0.577576000	-1.767000000
C	3.551357000	-2.038065000	-2.038221000
O	2.817174000	0.175884000	-1.348384000
H	4.285943000	-0.148441000	-2.712563000
H	4.405008000	-2.583515000	-2.454630000
C	5.003883000	-0.423567000	-0.726518000
H	5.196679000	0.632818000	-0.536237000
H	4.741075000	-0.921883000	0.209430000
Cl	6.590780000	-1.142329000	-1.274791000
H	2.724027000	-2.092677000	-2.754726000
H	3.241426000	-2.544197000	-1.117308000
C	-0.942331000	0.501057000	1.504669000
C	-1.501164000	1.738723000	2.147844000
O	0.461037000	0.356777000	1.616213000
H	-1.327732000	0.344850000	0.482601000
H	-2.590853000	1.663559000	2.231231000
C	-0.803038000	-0.708158000	2.295964000
H	-0.602653000	-1.655917000	1.821255000
H	-0.626446000	-0.614563000	3.357505000
H	-1.255132000	2.626211000	1.557691000
H	-1.080543000	1.861908000	3.150382000
C	-4.579297000	0.733597000	-0.183843000
C	-5.436120000	1.498172000	0.793373000
O	-5.408118000	-0.291009000	-0.772785000
H	-3.724288000	0.287606000	0.331143000
H	-4.868467000	2.320128000	1.237264000
C	-4.080021000	1.539455000	-1.371779000
H	-3.450497000	0.922316000	-2.020934000
H	-4.915621000	1.939759000	-1.951247000
Cl	-3.073118000	2.937195000	-0.852914000
H	-5.753546000	0.815815000	1.583955000

H	-6.315962000	1.912562000	0.290219000
C	-3.629018000	-2.017615000	-0.586300000
C	-5.027425000	-1.590507000	-0.865219000
C	-3.002270000	-2.086715000	0.595283000
C	-3.587414000	-1.570480000	1.888582000
O	-2.761289000	-1.320108000	2.830277000
O	-4.823326000	-1.409361000	1.916648000
O	-5.820541000	-2.379745000	-1.334711000
H	-3.152271000	-2.445838000	-1.467624000
H	-2.011758000	-2.543157000	0.621754000

82

A_INT9 E = -3228.4823328

Al	1.846881000	-0.104967000	0.217914000
O	3.152197000	0.790996000	1.164141000
O	2.154101000	-1.833814000	0.823894000
N	1.269159000	1.623953000	-0.750705000
N	0.297921000	-0.808409000	-0.983278000
C	3.142307000	2.036290000	1.512022000
C	1.254655000	-2.741521000	1.032081000
C	1.471295000	-3.677084000	2.079644000
C	3.880377000	2.419497000	2.665627000
C	0.548589000	1.398612000	-1.865066000
C	1.768924000	2.847670000	-0.473499000
C	-0.043190000	0.046234000	-1.967760000
C	3.914504000	3.720720000	3.121001000
C	-0.245995000	-2.042745000	-0.906164000
C	2.526606000	4.397468000	1.288691000
C	2.475505000	3.081768000	0.783136000
C	3.218513000	4.730800000	2.439084000
C	0.329677000	2.392335000	-2.812633000
C	0.068683000	-2.904665000	0.235023000
C	-0.946654000	-0.309243000	-2.965999000
C	1.587368000	3.884701000	-1.416383000
C	0.879907000	3.650757000	-2.579402000
C	0.573764000	-4.683955000	2.366741000
C	-1.462414000	-1.603760000	-2.953143000
C	-0.820055000	-3.953406000	0.558956000
C	-1.113912000	-2.471452000	-1.934995000
C	-0.597595000	-4.827674000	1.607223000
H	4.399035000	1.622910000	3.191151000
H	4.472613000	3.959605000	4.024572000
H	3.209588000	5.751590000	2.809895000
H	1.966493000	5.176038000	0.775149000
H	2.378320000	-3.544655000	2.661987000

H	0.771655000	-5.362477000	3.194089000
H	-1.323575000	-5.601034000	1.839063000
H	-1.741877000	-4.056971000	-0.010734000
H	-1.488729000	-3.488843000	-1.935175000
H	-2.136439000	-1.931197000	-3.741313000
H	-1.240624000	0.400594000	-3.731176000
H	-0.250170000	2.196774000	-3.706847000
H	0.755545000	4.444696000	-3.311299000
H	2.039379000	4.853523000	-1.238965000
C	3.688555000	-1.246234000	-1.832588000
C	2.914941000	-2.490894000	-2.262814000
O	2.893023000	-0.249229000	-1.310234000
H	4.207317000	-0.858241000	-2.734029000
H	3.569051000	-3.221456000	-2.751832000
C	4.774824000	-1.560313000	-0.807054000
H	5.275841000	-0.641853000	-0.499257000
H	4.363890000	-2.066586000	0.068695000
Cl	6.083828000	-2.654224000	-1.474991000
H	2.126012000	-2.206780000	-2.968794000
H	2.444397000	-2.976640000	-1.401254000
C	-0.723563000	0.495600000	1.638419000
C	-1.030659000	1.982199000	1.823032000
O	0.609865000	0.227393000	1.562142000
H	-1.291998000	0.161616000	0.729622000
H	-2.100859000	2.166287000	1.985026000
C	-1.254047000	-0.332905000	2.813618000
H	-1.045676000	-1.398526000	2.656220000
H	-0.753303000	-0.021412000	3.733034000
H	-0.715942000	2.544922000	0.938128000
H	-0.472809000	2.372121000	2.682423000
C	-4.577533000	1.184937000	-0.450988000
C	-5.104314000	2.414160000	0.244848000
O	-5.638242000	0.198873000	-0.443766000
H	-3.691010000	0.804315000	0.065941000
H	-4.345734000	3.200620000	0.245926000
C	-4.243164000	1.376051000	-1.920324000
H	-3.893710000	0.437317000	-2.360314000
H	-5.112453000	1.736836000	-2.475816000
Cl	-2.922599000	2.571309000	-2.136380000
H	-5.356517000	2.165056000	1.277039000
H	-5.999143000	2.789466000	-0.261605000
C	-4.051523000	-1.630734000	0.076451000
C	-5.426304000	-1.102185000	-0.146728000
C	-3.238393000	-1.418873000	1.120570000

C	-3.607030000	-0.491897000	2.217634000
O	-2.665741000	-0.118345000	3.091803000
O	-4.749930000	-0.079172000	2.348050000
O	-6.365923000	-1.867574000	-0.175196000
H	-3.752014000	-2.358519000	-0.676534000
H	-2.297169000	-1.958329000	1.169057000

72

A_INT10 E = -3035.3653

Al	1.238651000	-0.171050000	0.053191000
O	1.751486000	1.214756000	1.140993000
O	-0.090498000	-0.807315000	1.155765000
N	2.608047000	0.331321000	-1.379115000
N	0.658891000	-1.466926000	-1.436312000
C	2.819950000	1.943091000	1.109265000
C	-1.224221000	-1.361456000	0.836362000
C	-2.290509000	-1.275230000	1.767476000
C	3.105360000	2.726175000	2.261982000
C	2.539216000	-0.435971000	-2.485876000
C	3.536548000	1.313796000	-1.266053000
C	1.520413000	-1.507567000	-2.468949000
C	4.224050000	3.523051000	2.354860000
C	-0.416602000	-2.286064000	-1.393232000
C	4.870216000	2.848303000	0.146905000
C	3.724176000	2.033003000	-0.005401000
C	5.133177000	3.581240000	1.287514000
C	3.350628000	-0.215360000	-3.592724000
C	-1.450645000	-2.086876000	-0.378041000
C	1.428276000	-2.475834000	-3.464412000
C	4.330396000	1.614624000	-2.398844000
C	4.236737000	0.856972000	-3.546800000
C	-3.531087000	-1.822111000	1.512026000
C	0.404623000	-3.415040000	-3.371426000
C	-2.738390000	-2.617506000	-0.607425000
C	-0.523700000	-3.315147000	-2.353393000
C	-3.770368000	-2.491873000	0.303617000
H	2.393337000	2.650784000	3.078799000
H	4.406898000	4.095303000	3.262120000
H	6.036678000	4.179935000	1.355419000
H	5.605603000	2.884135000	-0.650551000
H	-2.087661000	-0.734469000	2.688078000
H	-4.334123000	-1.709412000	2.236909000
H	-4.758330000	-2.877962000	0.072182000
H	-2.944584000	-3.105342000	-1.556700000
H	-1.324358000	-4.040619000	-2.275325000

H	0.329468000	-4.216590000	-4.101398000
H	2.144352000	-2.513861000	-4.276783000
H	3.265876000	-0.831051000	-4.480041000
H	4.848831000	1.100756000	-4.411097000
H	5.006262000	2.458994000	-2.376501000
C	2.633234000	-2.369660000	1.467685000
C	1.722214000	-3.542561000	1.118950000
O	2.562478000	-1.332639000	0.559490000
H	3.672945000	-2.755028000	1.492997000
H	1.868151000	-4.386151000	1.802575000
C	2.345686000	-1.792568000	2.850965000
H	2.993358000	-0.934792000	3.040966000
H	1.300041000	-1.492444000	2.946738000
Cl	2.674492000	-2.985289000	4.198154000
H	1.937542000	-3.880537000	0.098843000
H	0.668937000	-3.245258000	1.163701000
C	-4.648980000	1.314295000	-0.292563000
C	-4.847317000	0.774853000	-1.686796000
O	-4.451753000	2.744082000	-0.386451000
H	-3.779264000	0.839668000	0.177849000
H	-4.996861000	-0.307841000	-1.652719000
C	-5.871043000	1.228274000	0.614854000
H	-5.613379000	1.502172000	1.640898000
H	-6.643447000	1.907453000	0.247494000
Cl	-6.632099000	-0.404247000	0.691795000
H	-3.953297000	0.993293000	-2.273544000
H	-5.719789000	1.236194000	-2.162026000
C	-2.451501000	2.661326000	1.074342000
C	-3.367286000	3.373256000	0.136414000
C	-1.383990000	1.925919000	0.744372000
C	-1.069056000	1.603020000	-0.685903000
O	0.033683000	0.982556000	-0.920254000
O	-1.880044000	1.959925000	-1.554386000
O	-3.265534000	4.568328000	-0.041613000
H	-2.629827000	2.915269000	2.119376000
H	-0.704859000	1.562634000	1.512247000

81

A_TS5 E = -3414.6176935 Imaginary Frequency = 212.6543

Al	-0.319195000	-0.338570000	-0.530492000
O	-0.842184000	-1.483776000	-1.812014000
O	0.066193000	1.143256000	-1.456814000
N	-0.745494000	-1.775117000	0.869601000
N	0.271144000	0.594477000	1.208804000
C	-1.821287000	-2.335962000	-1.785917000

C	-0.029213000	2.390512000	-1.071207000
C	-0.457249000	3.363750000	-1.999015000
C	-2.510138000	-2.623522000	-2.989761000
C	-0.064854000	-1.613275000	2.025297000
C	-1.464900000	-2.899534000	0.638761000
C	0.563782000	-0.289758000	2.189550000
C	-3.575606000	-3.500120000	-3.016259000
C	0.744328000	1.866724000	1.264324000
C	-3.316649000	-3.914680000	-0.664477000
C	-2.214885000	-3.036186000	-0.602037000
C	-4.003925000	-4.142998000	-1.842889000
C	-0.047814000	-2.588493000	3.013085000
C	0.348303000	2.819061000	0.235951000
C	1.373226000	0.042881000	3.266663000
C	-1.470042000	-3.920058000	1.613248000
C	-0.762280000	-3.763835000	2.788486000
C	-0.516337000	4.700625000	-1.655345000
C	1.909952000	1.327490000	3.314779000
C	0.293900000	4.192108000	0.547358000
C	1.588344000	2.237931000	2.328332000
C	-0.132795000	5.129472000	-0.376024000
H	-2.194371000	-2.077180000	-3.872242000
H	-4.106543000	-3.670021000	-3.950345000
H	-4.866925000	-4.801855000	-1.854739000
H	-3.658438000	-4.399061000	0.247854000
H	-0.781820000	3.005469000	-2.970446000
H	-0.885981000	5.422692000	-2.379523000
H	-0.200613000	6.177474000	-0.099998000
H	0.541309000	4.514617000	1.556525000
H	2.036061000	3.224700000	2.329777000
H	2.606772000	1.601538000	4.100398000
H	1.624801000	-0.693474000	4.020548000
H	0.497711000	-2.434045000	3.936279000
H	-0.760397000	-4.554427000	3.533666000
H	-2.010400000	-4.838364000	1.416191000
C	6.156821000	-0.724091000	-0.424306000
C	6.494420000	-2.113463000	0.053048000
O	5.994774000	0.102106000	0.752551000
H	5.224904000	-0.738174000	-0.995207000
H	6.653738000	-2.779128000	-0.798788000
C	7.252227000	-0.040947000	-1.225199000
H	6.955294000	0.973252000	-1.501883000
H	8.184484000	0.000875000	-0.656307000
Cl	7.603440000	-0.914630000	-2.757009000

H	5.662683000	-2.493502000	0.647910000
H	7.403010000	-2.102993000	0.664025000
C	4.104385000	1.298710000	-0.299652000
C	4.940080000	0.940484000	0.884542000
C	3.034718000	0.638909000	-0.760746000
C	2.601589000	-0.674573000	-0.185518000
O	1.427413000	-1.092436000	-0.511701000
O	3.388150000	-1.281989000	0.557135000
O	4.790109000	1.511967000	1.945073000
H	4.346532000	2.279571000	-0.709543000
H	2.421339000	1.070304000	-1.547768000
C	-3.146991000	0.798703000	0.951817000
C	-2.667296000	2.110772000	1.533550000
O	-2.405666000	0.398899000	-0.223598000
H	-4.181874000	0.933402000	0.632418000
H	-3.407088000	2.483520000	2.248369000
C	-3.168090000	-0.346248000	1.950096000
H	-3.424985000	-1.287234000	1.458306000
H	-2.234444000	-0.453422000	2.504199000
Cl	-4.437014000	-0.067138000	3.212183000
H	-2.563482000	2.845831000	0.733408000
H	-1.715030000	2.006857000	2.062273000
C	-4.526549000	-0.164682000	-1.476084000
C	-3.227564000	0.572163000	-1.578235000
C	-5.600438000	0.621159000	-1.402049000
C	-5.238885000	2.072094000	-1.310895000
O	-6.081964000	2.968635000	-1.259514000
O	-3.942807000	2.221979000	-1.279088000
O	-2.522589000	0.574490000	-2.558654000
H	-4.514549000	-1.250067000	-1.558284000
H	-6.636089000	0.296995000	-1.414114000

81

A_INT11 E = -3414.6588341

Al	0.213294000	0.058128000	1.123632000
O	1.127223000	-0.957044000	2.311106000
O	-0.496383000	1.279585000	2.267812000
N	0.749457000	-1.179100000	-0.433397000
N	-0.427923000	1.156054000	-0.464492000
C	1.972058000	-1.930144000	2.140910000
C	-0.543517000	2.575351000	2.110385000
C	-0.538329000	3.390350000	3.267743000
C	2.791746000	-2.293846000	3.239485000
C	0.069107000	-0.889134000	-1.564067000
C	1.322390000	-2.394734000	-0.269966000

C	-0.474754000	0.475919000	-1.627717000
C	3.734075000	-3.299693000	3.151274000
C	-0.771577000	2.465781000	-0.415768000
C	3.097032000	-3.685170000	0.872664000
C	2.120390000	-2.671070000	0.925121000
C	3.903676000	-4.005763000	1.952516000
C	-0.109417000	-1.815512000	-2.583283000
C	-0.641946000	3.216934000	0.835331000
C	-0.950045000	1.042196000	-2.800508000
C	1.151593000	-3.372619000	-1.269368000
C	0.433389000	-3.084316000	-2.414351000
C	-0.555309000	4.769538000	3.188524000
C	-1.371852000	2.363802000	-2.760674000
C	-0.631789000	4.625658000	0.795160000
C	-1.277353000	3.075292000	-1.579668000
C	-0.583552000	5.403155000	1.939585000
H	2.651719000	-1.728347000	4.156101000
H	4.353174000	-3.534747000	4.014778000
H	4.658553000	-4.781137000	1.861650000
H	3.256023000	-4.212848000	-0.065053000
H	-0.492369000	2.876895000	4.223693000
H	-0.527451000	5.362027000	4.100896000
H	-0.557290000	6.486154000	1.863298000
H	-0.625068000	5.125031000	-0.169776000
H	-1.608553000	4.105753000	-1.543881000
H	-1.759727000	2.840942000	-3.655654000
H	-0.978374000	0.473442000	-3.720728000
H	-0.702255000	-1.567161000	-3.455346000
H	0.276879000	-3.850868000	-3.168119000
H	1.550712000	-4.368000000	-1.115828000
C	-6.006640000	-1.554133000	-0.180169000
C	-6.206512000	-3.034940000	0.018021000
O	-5.413667000	-1.377182000	-1.488121000
H	-5.329357000	-1.162152000	0.583342000
H	-6.681903000	-3.227718000	0.983041000
C	-7.286641000	-0.737312000	-0.221771000
H	-7.069305000	0.316239000	-0.411792000
H	-7.964688000	-1.108997000	-0.994208000
Cl	-8.175772000	-0.807732000	1.341374000
H	-5.232994000	-3.526933000	-0.001232000
H	-6.838913000	-3.450577000	-0.773389000
C	-3.997548000	0.442696000	-0.616611000
C	-4.341198000	-0.566740000	-1.664251000
C	-3.121550000	0.256286000	0.376463000

C	-2.465688000	-1.075121000	0.595016000
O	-1.304666000	-1.069867000	1.147838000
O	-3.074176000	-2.089137000	0.225052000
O	-3.773511000	-0.587477000	-2.735656000
H	-4.417569000	1.432020000	-0.800317000
H	-2.824883000	1.086157000	1.016186000
C	4.567859000	-0.221169000	-0.702111000
C	4.174215000	-1.170678000	-1.807697000
O	3.424298000	-0.010500000	0.157613000
H	4.889141000	0.741172000	-1.116476000
H	5.011713000	-1.311236000	-2.496097000
C	5.614461000	-0.767898000	0.253638000
H	5.827908000	-0.053304000	1.051074000
H	5.277443000	-1.711035000	0.694912000
Cl	7.172750000	-1.093493000	-0.588354000
H	3.327510000	-0.749702000	-2.354022000
H	3.899240000	-2.147273000	-1.394714000
C	3.635050000	2.452332000	0.051287000
C	2.851005000	1.200558000	0.249973000
C	3.557701000	3.122929000	-1.099199000
C	2.741441000	2.510653000	-2.213023000
O	2.585341000	3.145334000	-3.268618000
O	2.295525000	1.350988000	-1.902306000
O	1.789410000	1.259849000	0.890849000
H	4.166832000	2.815510000	0.930315000
H	4.044726000	4.084159000	-1.247879000

61

A_INT12 E = -2381.9396082

Al	-1.681062000	0.574860000	-0.232787000
O	-3.000885000	-0.072541000	-1.241913000
O	-2.277083000	2.263024000	-0.323901000
N	-1.849367000	-1.003265000	1.021666000
N	-0.459747000	1.170490000	1.256683000
C	-3.852461000	-1.053700000	-1.130032000
C	-1.481087000	3.314009000	-0.381996000
C	-1.895921000	4.421232000	-1.150202000
C	-4.910010000	-1.124630000	-2.065519000
C	-0.895062000	-1.030085000	1.993541000
C	-2.659558000	-2.091674000	0.833997000
C	-0.132857000	0.214661000	2.149772000
C	-5.862669000	-2.119010000	-1.997160000
C	0.166506000	2.375589000	1.264789000
C	-4.762576000	-3.051507000	-0.077446000
C	-3.753844000	-2.060060000	-0.124253000

C	-5.800991000	-3.089361000	-0.984946000
C	-0.662631000	-2.144853000	2.780884000
C	-0.229839000	3.390519000	0.300511000
C	0.848024000	0.405690000	3.110275000
C	-2.415387000	-3.256863000	1.593849000
C	-1.426351000	-3.285990000	2.552303000
C	-1.079114000	5.522937000	-1.320495000
C	1.505811000	1.633600000	3.138090000
C	0.590443000	4.517540000	0.080995000
C	1.159550000	2.616478000	2.234501000
C	0.187597000	5.567665000	-0.721409000
H	-4.942920000	-0.353896000	-2.828628000
H	-6.671112000	-2.141094000	-2.723367000
H	-6.564645000	-3.856207000	-0.906481000
H	-4.750899000	-3.791652000	0.716039000
H	-2.866884000	4.353929000	-1.630461000
H	-1.415649000	6.349441000	-1.941254000
H	0.847576000	6.413352000	-0.885466000
H	1.579499000	4.547291000	0.529340000
H	1.657521000	3.576728000	2.264340000
H	2.289897000	1.817246000	3.865778000
H	1.106586000	-0.377433000	3.812469000
H	0.113575000	-2.134635000	3.535792000
H	-1.243481000	-4.192939000	3.120756000
H	-2.997889000	-4.147215000	1.400291000
C	4.018979000	-1.735467000	-0.410910000
C	3.597318000	-2.794958000	0.575219000
O	4.137508000	-0.495471000	0.333580000
H	3.259507000	-1.619804000	-1.189447000
H	3.514922000	-3.763950000	0.077405000
C	5.383118000	-1.961951000	-1.039663000
H	5.662121000	-1.121643000	-1.679578000
H	6.152641000	-2.098219000	-0.275857000
Cl	5.382230000	-3.428140000	-2.072348000
H	2.624111000	-2.529392000	0.992866000
H	4.327173000	-2.880531000	1.386243000
C	3.196960000	0.772168000	-1.572631000
C	3.643135000	0.660151000	-0.148905000
C	1.952564000	0.561286000	-2.017093000
C	0.898439000	0.019383000	-1.123365000
O	-0.306493000	0.419276000	-1.411684000
O	1.197774000	-0.719396000	-0.179360000
O	3.657759000	1.647572000	0.555985000
H	3.933994000	1.234589000	-2.227380000

H	1.666908000	0.854144000	-3.023573000

81

A_INT12' E = -3414.6722844

Al	-0.500104000	0.742330000	1.400661000
O	-0.553047000	2.332119000	2.262070000
O	-0.507929000	-0.352252000	2.870637000
N	-0.634239000	1.653234000	-0.430813000
N	-0.206421000	-0.876016000	0.193198000
C	-0.853571000	3.522380000	1.854950000
C	0.249716000	-1.395241000	3.055997000
C	0.679633000	-1.697800000	4.371614000
C	-1.014604000	4.522340000	2.852453000
C	-0.573381000	0.791183000	-1.470678000
C	-0.830753000	2.982148000	-0.640372000
C	-0.379116000	-0.628278000	-1.120159000
C	-1.352241000	5.820766000	2.545178000
C	0.213231000	-2.094956000	0.615573000
C	-1.381313000	5.246617000	0.216971000
C	-1.014600000	3.905755000	0.480565000
C	-1.551170000	6.193998000	1.207230000
C	-0.642773000	1.212276000	-2.792330000
C	0.639607000	-2.283042000	2.002164000
C	-0.305885000	-1.638243000	-2.073386000
C	-0.855186000	3.451107000	-1.974809000
C	-0.763324000	2.575643000	-3.035633000
C	1.500202000	-2.776146000	4.637830000
C	-0.015354000	-2.928431000	-1.642144000
C	1.494348000	-3.362977000	2.309772000
C	0.262714000	-3.155904000	-0.309345000
C	1.930036000	-3.613754000	3.598343000
H	-0.870711000	4.199226000	3.878965000
H	-1.477329000	6.551024000	3.342141000
H	-1.845179000	7.206686000	0.947723000
H	-1.567700000	5.558681000	-0.805068000
H	0.351906000	-1.024166000	5.157769000
H	1.827610000	-2.963560000	5.658769000
H	2.608380000	-4.439256000	3.793461000
H	1.876916000	-3.977957000	1.500445000
H	0.532497000	-4.148381000	0.028836000
H	0.027645000	-3.746408000	-2.355937000
H	-0.470221000	-1.430046000	-3.122609000
H	-0.588671000	0.502722000	-3.607379000
H	-0.786413000	2.950767000	-4.055225000
H	-0.935051000	4.510628000	-2.174934000

C	5.119092000	-0.023390000	-1.578658000
C	4.574580000	0.624542000	-2.826490000
O	4.613287000	-1.377833000	-1.545558000
H	4.776197000	0.518228000	-0.693249000
H	4.950449000	1.646671000	-2.918708000
C	6.630143000	-0.177668000	-1.547383000
H	6.947940000	-0.695796000	-0.639767000
H	6.987293000	-0.730270000	-2.420078000
Cl	7.464057000	1.416967000	-1.552312000
H	3.485475000	0.653161000	-2.761949000
H	4.871529000	0.060573000	-3.716907000
C	4.265317000	-1.231500000	0.900938000
C	4.054544000	-1.891560000	-0.422892000
C	3.491512000	-0.282304000	1.440550000
C	2.344267000	0.316124000	0.691078000
O	1.437432000	0.827968000	1.439648000
O	2.341352000	0.235893000	-0.550716000
O	3.511075000	-2.974106000	-0.496469000
H	5.024882000	-1.723364000	1.508314000
H	3.622950000	0.006193000	2.481441000
C	-4.162760000	-0.951760000	-1.743526000
C	-4.557307000	0.492793000	-1.929382000
O	-5.229229000	-1.636200000	-1.048879000
H	-3.225486000	-1.027312000	-1.178937000
H	-3.753080000	1.042320000	-2.427483000
C	-4.061600000	-1.760591000	-3.031727000
H	-3.646101000	-2.753117000	-2.840185000
H	-5.053317000	-1.864803000	-3.476482000
Cl	-3.020754000	-0.997220000	-4.291616000
H	-4.740943000	0.942593000	-0.951779000
H	-5.462644000	0.570059000	-2.540549000
C	-3.730617000	-2.561627000	0.715336000
C	-5.053196000	-2.412635000	0.052184000
C	-3.017172000	-1.607174000	1.327631000
C	-3.375069000	-0.152617000	1.270180000
O	-2.390976000	0.679265000	1.220902000
O	-4.568516000	0.158895000	1.211876000
O	-6.004232000	-3.058093000	0.440919000
H	-3.434216000	-3.603507000	0.823086000
H	-2.126204000	-1.883779000	1.886569000

71

A_INT13 E = -2575.0652834

Al	-1.621341000	0.191160000	-0.481455000
O	-2.810642000	-0.798807000	-1.401098000

O	-1.926117000	1.736944000	-1.394262000
N	-1.534252000	-1.211803000	0.975516000
N	-0.640096000	1.235481000	0.944323000
C	-2.839867000	-2.106430000	-1.499178000
C	-0.988248000	2.637150000	-1.603982000
C	-0.939711000	3.288167000	-2.854047000
C	-3.370031000	-2.674218000	-2.678976000
C	-1.080825000	-0.755973000	2.165367000
C	-1.955383000	-2.493941000	0.837471000
C	-0.559328000	0.621415000	2.144522000
C	-3.416033000	-4.041527000	-2.867380000
C	-0.053771000	2.440181000	0.736043000
C	-2.450535000	-4.376354000	-0.697664000
C	-2.402593000	-2.985368000	-0.461889000
C	-2.937755000	-4.908968000	-1.875876000
C	-1.060547000	-1.553168000	3.300830000
C	-0.035986000	3.011384000	-0.608046000
C	0.041418000	1.229464000	3.237670000
C	-1.967846000	-3.330987000	1.973940000
C	-1.525865000	-2.861617000	3.193964000
C	0.035244000	4.226305000	-3.140312000
C	0.587023000	2.500223000	3.066931000
C	0.953840000	3.960463000	-0.937943000
C	0.547312000	3.101269000	1.825335000
C	1.002900000	4.559521000	-2.183333000
H	-3.716327000	-1.984960000	-3.442814000
H	-3.810231000	-4.443103000	-3.797570000
H	-2.939276000	-5.983101000	-2.030450000
H	-2.059602000	-5.054494000	0.055619000
H	-1.683313000	3.000256000	-3.590815000
H	0.058729000	4.692998000	-4.122161000
H	1.791212000	5.268260000	-2.416520000
H	1.733804000	4.178821000	-0.214427000
H	0.990693000	4.077729000	1.676846000
H	1.062641000	3.006845000	3.901159000
H	0.106228000	0.722865000	4.193056000
H	-0.693394000	-1.168799000	4.244685000
H	-1.545309000	-3.508236000	4.066320000
H	-2.354931000	-4.338512000	1.889904000
C	4.435815000	-1.208209000	0.354223000
C	4.186965000	-2.160758000	1.495677000
O	4.264750000	0.131590000	0.882390000
H	3.707521000	-1.378047000	-0.443712000
H	4.329526000	-3.192319000	1.165223000

C	5.846169000	-1.245706000	-0.208086000
H	5.977618000	-0.483757000	-0.979920000
H	6.588663000	-1.088526000	0.578205000
Cl	6.199673000	-2.829650000	-0.973212000
H	3.160348000	-2.041254000	1.846206000
H	4.874380000	-1.961498000	2.323583000
C	3.285694000	0.869504000	-1.260188000
C	3.578643000	1.065484000	0.193489000
C	2.171632000	0.322560000	-1.759751000
C	1.135436000	-0.252587000	-0.858799000
O	-0.061015000	-0.260404000	-1.363908000
O	1.449753000	-0.623171000	0.279209000
O	3.316272000	2.119153000	0.736262000
H	3.993168000	1.366987000	-1.922433000
H	1.956049000	0.374012000	-2.824023000
C	-3.699709000	2.183602000	0.909852000
C	-4.099744000	2.548541000	2.301271000
O	-3.333151000	0.792560000	0.724870000
H	-3.008108000	2.853374000	0.398894000
H	-4.617150000	3.512074000	2.307726000
C	-4.493269000	1.313351000	0.047505000
H	-4.374701000	1.376429000	-1.031115000
H	-5.426873000	0.893604000	0.417925000
H	-3.218859000	2.641251000	2.944536000
H	-4.764154000	1.794763000	2.731162000

91

A_TS6 E = -3607.7488013 Imaginary Frequency= 443.1964

Al	-0.484387000	1.429159000	-0.635334000
O	-0.844222000	2.913694000	-1.613229000
O	-0.463947000	0.194016000	-1.972268000
N	-0.283639000	2.551082000	1.061301000
N	-0.186390000	-0.064920000	0.775353000
C	-0.389650000	4.118230000	-1.431950000
C	-0.066763000	-1.049900000	-2.003838000
C	0.162656000	-1.635431000	-3.272786000
C	-0.321925000	4.977028000	-2.558332000
C	-0.346028000	1.836943000	2.203798000
C	-0.116947000	3.896727000	1.084658000
C	-0.441494000	0.377579000	2.027445000
C	0.175684000	6.260468000	-2.471481000
C	-0.195448000	-1.394606000	0.507763000
C	0.536276000	5.957893000	-0.120651000
C	0.014844000	4.646665000	-0.161516000
C	0.629121000	6.760529000	-1.242157000

C	-0.262776000	2.435127000	3.455526000
C	0.104064000	-1.869831000	-0.844106000
C	-0.767012000	-0.482392000	3.067028000
C	-0.087262000	4.549146000	2.337237000
C	-0.151895000	3.821941000	3.509235000
C	0.565580000	-2.950548000	-3.408982000
C	-0.826032000	-1.845034000	2.800083000
C	0.519365000	-3.203886000	-1.024862000
C	-0.526516000	-2.302548000	1.532920000
C	0.754801000	-3.749774000	-2.274903000
H	-0.658783000	4.562126000	-3.503570000
H	0.232596000	6.877872000	-3.365469000
H	1.057360000	7.755619000	-1.169061000
H	0.914523000	6.346341000	0.820885000
H	0.016175000	-0.995502000	-4.138568000
H	0.750380000	-3.357622000	-4.400298000
H	1.098972000	-4.775360000	-2.368063000
H	0.692071000	-3.822295000	-0.147266000
H	-0.627236000	-3.354425000	1.302086000
H	-1.131771000	-2.545611000	3.571239000
H	-1.012970000	-0.094213000	4.048427000
H	-0.276571000	1.836881000	4.359027000
H	-0.115459000	4.331321000	4.468342000
H	-0.030283000	5.629523000	2.377663000
C	-6.163259000	-1.538655000	0.391944000
C	-6.781843000	-0.919260000	1.618945000
O	-5.248232000	-2.561219000	0.850708000
H	-5.615402000	-0.781907000	-0.174738000
H	-7.508068000	-0.154379000	1.333070000
C	-7.138786000	-2.269039000	-0.515161000
H	-6.615119000	-2.730758000	-1.355455000
H	-7.684780000	-3.039700000	0.034691000
Cl	-8.359915000	-1.150871000	-1.217408000
H	-5.994736000	-0.454120000	2.214181000
H	-7.291290000	-1.678137000	2.221705000
C	-3.665154000	-2.006154000	-0.964066000
C	-4.012555000	-2.694689000	0.313657000
C	-3.201543000	-0.758854000	-1.110143000
C	-3.056204000	0.188291000	0.041463000
O	-2.309944000	1.220211000	-0.161180000
O	-3.650957000	-0.065944000	1.098889000
O	-3.252928000	-3.498122000	0.813809000
H	-3.671310000	-2.677700000	-1.823152000
H	-2.854632000	-0.421454000	-2.083292000

C	2.545908000	1.021771000	-0.483580000
C	3.564968000	1.724944000	0.367935000
O	1.404003000	1.803252000	-0.797920000
H	2.300689000	0.011964000	-0.117049000
H	4.526717000	1.201984000	0.321661000
C	2.607424000	1.155931000	-1.928477000
H	2.044577000	0.481354000	-2.559759000
H	3.009017000	2.065265000	-2.348537000
H	3.234332000	1.766397000	1.411468000
H	3.713352000	2.745340000	0.002013000
C	4.852637000	-1.667255000	1.412931000
C	5.855019000	-0.868524000	2.207535000
O	5.547506000	-2.825798000	0.910162000
H	4.483231000	-1.067725000	0.578464000
H	5.382193000	0.016802000	2.641043000
C	3.677595000	-2.202210000	2.211595000
H	2.998273000	-2.769338000	1.567409000
H	4.009355000	-2.832279000	3.040743000
Cl	2.700424000	-0.862715000	2.921090000
H	6.658097000	-0.549252000	1.540335000
H	6.281430000	-1.471109000	3.016224000
C	4.046662000	-2.906847000	-1.049321000
C	5.292745000	-3.309962000	-0.339784000
C	3.830003000	-1.786831000	-1.749302000
C	4.847925000	-0.678588000	-1.850552000
O	4.498617000	0.363150000	-2.502576000
O	5.942371000	-0.868163000	-1.278280000
O	5.992727000	-4.207662000	-0.754153000
H	3.293587000	-3.695125000	-1.056771000
H	2.885006000	-1.681685000	-2.285668000

91

A_INT14 E = -3607.7904605

Al	-0.295504000	1.486498000	-0.836136000
O	-0.704156000	3.111638000	-1.559733000
O	-0.759954000	0.401878000	-2.244146000
N	0.200911000	2.363500000	0.945296000
N	-0.059854000	-0.209729000	0.380859000
C	-0.078613000	4.224170000	-1.330605000
C	-0.514866000	-0.851710000	-2.464922000
C	-0.513406000	-1.297285000	-3.814071000
C	-0.060783000	5.206223000	-2.355811000
C	0.219330000	1.529989000	2.003487000
C	0.500528000	3.678012000	1.083736000
C	-0.023391000	0.107345000	1.694693000

C	0.605292000	6.405409000	-2.211897000
C	-0.278530000	-1.492427000	-0.010438000
C	1.247725000	5.777788000	0.010869000
C	0.560987000	4.549319000	-0.087440000
C	1.288115000	6.697118000	-1.021727000
C	0.503744000	1.978117000	3.289742000
C	-0.304926000	-1.830840000	-1.436467000
C	-0.192936000	-0.846807000	2.691411000
C	0.743284000	4.182800000	2.379442000
C	0.747723000	3.335811000	3.470893000
C	-0.334592000	-2.623632000	-4.151256000
C	-0.436749000	-2.160963000	2.311812000
C	-0.158349000	-3.179342000	-1.826104000
C	-0.479902000	-2.485407000	0.970379000
C	-0.160023000	-3.587116000	-3.147836000
H	-0.576697000	4.951369000	-3.276882000
H	0.614842000	7.118548000	-3.034088000
H	1.848016000	7.621195000	-0.911553000
H	1.798830000	6.000770000	0.921024000
H	-0.664347000	-0.534219000	-4.572441000
H	-0.331201000	-2.919026000	-5.198519000
H	-0.020914000	-4.634795000	-3.395818000
H	-0.020939000	-3.938298000	-1.061298000
H	-0.746207000	-3.490319000	0.673017000
H	-0.626731000	-2.924336000	3.061170000
H	-0.173822000	-0.564556000	3.736995000
H	0.546353000	1.288305000	4.123894000
H	0.942076000	3.728507000	4.465415000
H	0.905526000	5.244515000	2.518979000
C	-6.028449000	-1.103929000	0.923971000
C	-6.257321000	-0.679846000	2.352343000
O	-5.159664000	-2.258961000	0.958645000
H	-5.547127000	-0.297472000	0.365443000
H	-6.934072000	0.177411000	2.390913000
C	-7.271658000	-1.580527000	0.192406000
H	-7.023605000	-1.908787000	-0.819548000
H	-7.752268000	-2.402323000	0.729261000
Cl	-8.494522000	-0.270212000	0.025827000
H	-5.299960000	-0.394450000	2.790822000
H	-6.694508000	-1.497772000	2.934605000
C	-3.973287000	-1.474149000	-1.062519000
C	-4.092688000	-2.365652000	0.128099000
C	-3.418758000	-0.256678000	-1.104672000
C	-2.898067000	0.432959000	0.121707000

O	-2.087128000	1.407316000	-0.076586000
O	-3.276163000	0.018371000	1.231435000
O	-3.334737000	-3.299158000	0.292305000
H	-4.252836000	-1.970608000	-1.992459000
H	-3.263588000	0.236442000	-2.060711000
C	2.574982000	0.868715000	-1.032118000
C	3.583284000	1.623322000	-0.167827000
O	1.466907000	1.622647000	-1.301632000
H	2.327775000	-0.081288000	-0.496696000
H	4.516142000	1.062314000	-0.026437000
C	3.174524000	0.475603000	-2.385465000
H	2.442932000	-0.087903000	-2.977132000
H	3.438566000	1.377834000	-2.941969000
H	3.151893000	1.826956000	0.818281000
H	3.822324000	2.584477000	-0.637432000
C	4.446703000	-1.775682000	1.543590000
C	5.777642000	-1.124010000	1.824191000
O	4.716462000	-3.165608000	1.236509000
H	3.947628000	-1.280497000	0.704365000
H	5.639167000	-0.068620000	2.069693000
C	3.489989000	-1.823386000	2.724148000
H	2.522270000	-2.240797000	2.427366000
H	3.911578000	-2.419841000	3.536798000
Cl	3.163445000	-0.182188000	3.376754000
H	6.409574000	-1.201506000	0.937583000
H	6.277365000	-1.617009000	2.664035000
C	3.095818000	-3.212447000	-0.639160000
C	4.160599000	-3.828237000	0.198044000
C	3.201023000	-2.208024000	-1.519038000
C	4.469539000	-1.465696000	-1.705826000
O	4.427064000	-0.253853000	-2.279360000
O	5.544948000	-1.930834000	-1.362310000
O	4.465256000	-4.987967000	0.020872000
H	2.149567000	-3.747842000	-0.581077000
H	2.326951000	-1.943651000	-2.109358000

81

A_INT3 (Full mode) E = -2631.6181171

Al	-0.037545000	0.830086000	0.076646000
O	-1.187361000	-0.227144000	-0.843979000
O	1.285253000	-0.370963000	-0.161106000
N	-1.241244000	2.395015000	-0.403390000
N	1.346594000	2.323494000	-0.177364000
C	-2.506712000	-0.220365000	-0.764450000
C	2.597150000	-0.348695000	-0.236976000

C	3.311741000	-1.576492000	-0.395548000
C	-3.241572000	-1.428273000	-0.955645000
C	-0.644434000	3.604765000	-0.422592000
C	-2.582987000	2.284671000	-0.578642000
C	0.801624000	3.566160000	-0.167491000
C	-4.617516000	-1.401528000	-0.745117000
C	2.690209000	2.169421000	0.008200000
C	-4.621615000	0.930218000	-0.309552000
C	-3.231459000	0.984265000	-0.524349000
C	-5.336846000	-0.251102000	-0.384283000
C	-1.343541000	4.778896000	-0.662315000
C	3.333331000	0.871967000	-0.155180000
C	1.537668000	4.711209000	0.090107000
C	-3.328999000	3.452440000	-0.856278000
C	-2.714425000	4.686492000	-0.895476000
C	4.698231000	-1.529782000	-0.481154000
C	2.889788000	4.563725000	0.389340000
C	4.738891000	0.832529000	-0.276711000
C	3.456927000	3.309713000	0.348621000
C	5.446041000	-0.343056000	-0.436557000
H	-5.164417000	-2.330443000	-0.852821000
H	-5.143604000	1.844257000	-0.036814000
H	5.231637000	-2.464440000	-0.604278000
H	5.296107000	1.763782000	-0.288236000
H	4.500976000	3.193750000	0.604012000
H	3.491224000	5.426314000	0.660094000
H	1.062660000	5.684325000	0.109194000
H	-0.835922000	5.735363000	-0.692584000
H	-3.292909000	5.577551000	-1.120396000
H	-4.386151000	3.367003000	-1.073828000
C	-0.196874000	0.216919000	2.895636000
C	1.282837000	0.005132000	3.170379000
O	-0.427611000	1.016974000	1.772060000
H	-0.646230000	0.716884000	3.770410000
H	1.443571000	-0.537919000	4.106177000
C	-0.957278000	-1.078895000	2.650466000
H	-2.019579000	-0.875989000	2.493458000
H	-0.561817000	-1.581278000	1.762875000
Cl	-0.840905000	-2.263970000	4.007569000
H	1.784958000	0.975003000	3.241974000
H	1.754136000	-0.565133000	2.362643000
C	6.968914000	-0.308393000	-0.575468000
C	7.567474000	-1.707328000	-0.722106000
H	8.656478000	-1.636686000	-0.808946000

H	7.346273000	-2.339731000	0.144228000
H	7.198701000	-2.218651000	-1.617610000
C	7.582866000	0.349446000	0.668333000
H	7.224314000	1.375670000	0.801533000
H	7.331982000	-0.209668000	1.575586000
H	8.675014000	0.389311000	0.584586000
C	7.351749000	0.512587000	-1.814847000
H	6.934154000	0.070636000	-2.725403000
H	6.984033000	1.541843000	-1.747096000
H	8.441221000	0.556563000	-1.927299000
C	2.572915000	-2.914875000	-0.488274000
C	1.805417000	-3.182035000	0.814505000
H	2.495954000	-3.273430000	1.660765000
H	1.103091000	-2.380948000	1.041057000
H	1.237839000	-4.117135000	0.741071000
C	3.531036000	-4.092947000	-0.689325000
H	4.107498000	-4.007377000	-1.617242000
H	4.234116000	-4.205976000	0.143283000
H	2.948179000	-5.017491000	-0.750780000
C	1.623099000	-2.899882000	-1.695506000
H	0.882975000	-2.102562000	-1.627506000
H	2.189129000	-2.767570000	-2.624901000
H	1.088203000	-3.854687000	-1.763998000
C	-2.540947000	-2.722387000	-1.380195000
C	-6.836888000	-0.261761000	-0.087696000
C	-1.537821000	-3.168803000	-0.309569000
H	-1.070544000	-4.116265000	-0.604320000
H	-0.744733000	-2.432860000	-0.177443000
H	-2.033838000	-3.328151000	0.655340000
C	-3.530007000	-3.873483000	-1.584321000
H	-4.050501000	-4.144797000	-0.659284000
H	-4.282275000	-3.644923000	-2.347456000
H	-2.981060000	-4.759016000	-1.920233000
C	-1.821946000	-2.499769000	-2.720301000
H	-1.287474000	-3.410298000	-3.015948000
H	-2.544875000	-2.264389000	-3.509550000
H	-1.098011000	-1.686028000	-2.662074000
C	-7.450832000	-1.650915000	-0.260768000
H	-7.339064000	-2.023369000	-1.284751000
H	-7.001326000	-2.383909000	0.417305000
H	-8.522372000	-1.612318000	-0.039803000
C	-7.071646000	0.193679000	1.359402000
H	-6.575307000	-0.475662000	2.069441000
H	-6.685993000	1.203828000	1.531836000

H	-8.142759000	0.202060000	1.592303000
C	-7.558227000	0.704081000	-1.038304000
H	-8.635151000	0.713032000	-0.834578000
H	-7.193761000	1.730731000	-0.925967000
H	-7.413008000	0.411765000	-2.083555000

111

A_TS2 (Full mode) E = -3285.001811 Imaginary Frequency= 416.4784

Al	-0.030580000	-0.335583000	0.820547000
O	1.330938000	0.897512000	0.800864000
O	-1.443623000	0.745806000	0.352069000
N	1.291054000	-1.851825000	1.130527000
N	-1.315242000	-1.961384000	0.867127000
C	2.582273000	0.748738000	0.461527000
C	-2.600534000	0.485154000	-0.192039000
C	-3.290150000	1.524849000	-0.904628000
C	3.342287000	1.880093000	0.019369000
C	0.717517000	-2.984980000	1.584522000
C	2.636846000	-1.709752000	1.108659000
C	-0.754306000	-3.025289000	1.480740000
C	4.635658000	1.677690000	-0.449508000
C	-2.651191000	-1.905590000	0.662059000
C	4.556518000	-0.643535000	0.026466000
C	3.253230000	-0.515024000	0.541204000
C	5.262196000	0.422164000	-0.501630000
C	1.469104000	-4.051432000	2.061062000
C	-3.243184000	-0.786832000	-0.068574000
C	-1.508780000	-4.093258000	1.949375000
C	3.436975000	-2.751364000	1.631895000
C	2.855219000	-3.911483000	2.098829000
C	-4.553079000	1.260881000	-1.409250000
C	-2.892654000	-4.031381000	1.803687000
C	-4.514664000	-0.987126000	-0.650522000
C	-3.462338000	-2.947589000	1.168108000
C	-5.197491000	0.013780000	-1.310667000
H	5.180835000	2.535968000	-0.825746000
H	4.995821000	-1.637519000	-0.023833000
H	-5.072230000	2.052329000	-1.942781000
H	-4.950100000	-1.979509000	-0.602906000
H	-4.539699000	-2.875198000	1.086597000
H	-3.521305000	-4.824819000	2.198767000
H	-1.038746000	-4.936834000	2.440501000
H	0.994036000	-4.964225000	2.400035000
H	3.474842000	-4.710591000	2.496364000
H	4.511029000	-2.620423000	1.682840000

C	-0.983762000	0.368225000	3.535718000
C	-2.474819000	0.070364000	3.415907000
O	-0.199598000	-0.339611000	2.645648000
H	-0.676951000	0.107807000	4.568751000
H	-3.047592000	0.566260000	4.206769000
C	-0.662907000	1.844346000	3.332361000
H	0.408923000	2.013376000	3.444510000
H	-0.986463000	2.178077000	2.343052000
Cl	-1.482031000	2.942820000	4.538256000
H	-2.640384000	-1.009968000	3.494958000
H	-2.866889000	0.407745000	2.450149000
C	0.105145000	-1.402351000	-2.246104000
C	-0.387769000	-2.804249000	-2.028107000
O	0.262469000	-0.536614000	-1.129712000
H	-0.416959000	-0.896254000	-3.068139000
H	-0.179856000	-3.381170000	-2.934301000
C	1.530452000	-1.153156000	-2.157108000
H	1.977691000	-0.269651000	-2.588942000
H	2.126921000	-1.786923000	-1.519742000
Cl	2.613299000	-2.507980000	-3.994764000
H	-1.461604000	-2.831699000	-1.816834000
H	0.150059000	-3.280771000	-1.201643000
C	6.624255000	0.189229000	-1.154518000
C	6.444515000	-0.730714000	-2.370804000
H	6.020289000	-1.699323000	-2.089798000
H	5.760161000	-0.294110000	-3.104585000
H	7.408012000	-0.913302000	-2.863337000
C	7.577967000	-0.479916000	-0.154874000
H	8.553920000	-0.668412000	-0.619277000
H	7.735471000	0.152308000	0.726260000
H	7.187752000	-1.442687000	0.191526000
C	7.268992000	1.493454000	-1.624445000
H	7.425534000	2.194908000	-0.796730000
H	8.246466000	1.282933000	-2.072478000
H	6.660718000	1.996374000	-2.383627000
C	2.706666000	3.272895000	-0.015479000
C	1.543820000	3.269225000	-1.013718000
H	1.894280000	3.028827000	-2.024981000
H	0.793746000	2.530754000	-0.732871000
H	1.061678000	4.255238000	-1.046234000
C	2.189304000	3.674731000	1.374111000
H	1.435034000	2.975652000	1.735109000
H	3.008333000	3.708007000	2.102134000
H	1.736812000	4.673406000	1.330076000

C	3.697969000	4.352580000	-0.458399000
H	4.569627000	4.411193000	0.204126000
H	4.057067000	4.194061000	-1.481462000
H	3.198118000	5.327221000	-0.435285000
C	-2.586771000	2.848865000	-1.217682000
C	-3.539056000	3.866150000	-1.856560000
H	-2.990877000	4.793221000	-2.054579000
H	-3.942235000	3.520032000	-2.814615000
H	-4.381216000	4.111822000	-1.198488000
C	-1.483860000	2.535294000	-2.242355000
H	-0.892080000	3.431866000	-2.463286000
H	-0.806301000	1.759572000	-1.875196000
H	-1.929188000	2.181869000	-3.180089000
C	-1.987574000	3.524020000	0.028509000
H	-2.722188000	3.584984000	0.841013000
H	-1.114041000	2.992421000	0.403031000
H	-1.678635000	4.546769000	-0.220872000
C	-6.569554000	-0.177465000	-1.952336000
C	-7.110544000	-1.592044000	-1.747838000
H	-6.458365000	-2.346479000	-2.201022000
H	-7.221013000	-1.834258000	-0.684843000
H	-8.097834000	-1.685484000	-2.213573000
C	-7.566565000	0.812753000	-1.333467000
H	-7.245008000	1.849523000	-1.475486000
H	-8.556574000	0.704674000	-1.793784000
H	-7.670242000	0.643929000	-0.256358000
C	-6.477123000	0.088244000	-3.461929000
H	-6.127771000	1.104609000	-3.670706000
H	-5.778174000	-0.605576000	-3.940499000
H	-7.458264000	-0.031688000	-3.938045000

120

A_INT10 (Full mode) E = -3664.3507356

Al	-0.739230000	0.478345000	0.612841000
O	-1.996067000	-0.089654000	-0.610877000
O	0.603002000	-0.612733000	0.014503000
N	-1.978103000	1.945024000	1.259816000
N	0.572312000	1.525325000	1.777446000
C	-3.286056000	0.018503000	-0.589995000
C	1.812031000	-0.836410000	0.442977000
C	2.568062000	-1.898310000	-0.151895000
C	-4.060590000	-0.746046000	-1.525910000
C	-1.361690000	2.918873000	1.957085000
C	-3.313690000	1.971699000	1.038085000
C	0.081580000	2.710358000	2.195149000

C	-5.438861000	-0.793776000	-1.367927000
C	1.873788000	1.207590000	1.964301000
C	-5.381049000	0.776237000	0.416375000
C	-3.982438000	0.898419000	0.303800000
C	-6.129393000	-0.085938000	-0.367004000
C	-2.055399000	4.016321000	2.456569000
C	2.425225000	-0.061885000	1.482010000
C	0.875614000	3.660372000	2.824415000
C	-4.046883000	3.092425000	1.491228000
C	-3.420437000	4.100572000	2.196366000
C	3.803309000	-2.227200000	0.391522000
C	2.223722000	3.369580000	3.006719000
C	3.655903000	-0.494531000	2.008100000
C	2.719907000	2.154588000	2.583786000
C	4.363715000	-1.570040000	1.497196000
H	-6.011477000	-1.416668000	-2.046453000
H	-5.890989000	1.359836000	1.180213000
H	4.360579000	-3.039403000	-0.060773000
H	4.073776000	0.041572000	2.857009000
H	3.779564000	1.949376000	2.674654000
H	2.887314000	4.102539000	3.457300000
H	0.466405000	4.614157000	3.135496000
H	-1.550118000	4.784747000	3.029191000
H	-3.990711000	4.959607000	2.540176000
H	-5.100402000	3.174749000	1.253589000
C	-1.438598000	-1.751610000	2.497215000
C	-0.094379000	-2.416968000	2.773447000
O	-1.329879000	-0.445656000	2.066704000
H	-2.019797000	-1.773130000	3.441884000
H	-0.219120000	-3.396263000	3.248008000
C	-2.257416000	-2.495536000	1.449168000
H	-3.233191000	-2.023223000	1.309790000
H	-1.731141000	-2.510365000	0.492444000
Cl	-2.594849000	-4.239389000	1.871456000
H	0.498155000	-1.777546000	3.435518000
H	0.474931000	-2.558316000	1.848990000
C	4.310341000	1.589679000	-1.845691000
C	4.870030000	1.682412000	-0.450466000
O	4.226303000	2.939755000	-2.362863000
H	3.316853000	1.130480000	-1.819816000
H	4.939937000	0.686734000	-0.004923000
C	5.203649000	0.872585000	-2.845421000
H	4.715995000	0.800507000	-3.821081000
H	6.152838000	1.402820000	-2.956184000

Cl	5.598237000	-0.808231000	-2.337896000
H	4.192503000	2.289440000	0.154715000
H	5.865260000	2.141705000	-0.456980000
C	2.056114000	2.472714000	-3.452692000
C	3.113792000	3.409814000	-2.975348000
C	1.024046000	1.992795000	-2.750350000
C	0.868826000	2.200953000	-1.273919000
O	-0.244461000	1.777226000	-0.798090000
O	1.799413000	2.747387000	-0.655230000
O	3.075676000	4.584745000	-3.274511000
H	2.099377000	2.303692000	-4.528624000
H	0.250590000	1.415286000	-3.253290000
C	-7.635367000	-0.218049000	-0.136321000
C	-7.892054000	-0.723443000	1.290518000
H	-7.477915000	-0.038568000	2.037657000
H	-8.968399000	-0.819557000	1.482886000
H	-7.427323000	-1.702057000	1.449234000
C	-8.313156000	1.149158000	-0.307580000
H	-9.394224000	1.073339000	-0.134099000
H	-7.914983000	1.886194000	0.397946000
H	-8.156208000	1.540854000	-1.318360000
C	-8.287241000	-1.196757000	-1.113445000
H	-8.169410000	-0.873850000	-2.153684000
H	-7.863604000	-2.202644000	-1.023402000
H	-9.361309000	-1.268077000	-0.907898000
C	-3.360329000	-1.461919000	-2.687109000
C	-2.397975000	-2.551420000	-2.195084000
H	-1.565868000	-2.115065000	-1.642696000
H	-2.905277000	-3.276719000	-1.547503000
H	-1.984414000	-3.097504000	-3.053199000
C	-2.564029000	-0.433595000	-3.509877000
H	-1.823570000	0.077586000	-2.890581000
H	-2.038455000	-0.938176000	-4.331007000
H	-3.231418000	0.318372000	-3.946905000
C	-4.366024000	-2.128203000	-3.630128000
H	-4.929253000	-2.928401000	-3.136043000
H	-5.084170000	-1.410247000	-4.043241000
H	-3.826752000	-2.578522000	-4.470707000
C	5.717865000	-1.950870000	2.096736000
C	5.560167000	-2.249483000	3.593664000
H	6.526609000	-2.514350000	4.041081000
H	5.167776000	-1.384559000	4.138332000
H	4.867962000	-3.082309000	3.756525000
C	6.701083000	-0.783640000	1.918672000

H	7.675729000	-1.021733000	2.363920000
H	6.855642000	-0.562529000	0.856430000
H	6.329340000	0.131273000	2.394639000
C	6.321278000	-3.183555000	1.422733000
H	7.279874000	-3.432490000	1.891941000
H	5.667076000	-4.057439000	1.514436000
H	6.509242000	-3.012484000	0.356977000
C	1.992038000	-2.689889000	-1.332666000
C	1.561257000	-1.751814000	-2.474517000
H	0.768805000	-1.066642000	-2.167638000
H	2.415746000	-1.159369000	-2.828383000
H	1.192651000	-2.345348000	-3.321295000
C	3.015746000	-3.665656000	-1.922680000
H	3.315927000	-4.438227000	-1.205654000
H	2.566649000	-4.177798000	-2.780895000
H	3.916994000	-3.151083000	-2.274958000
C	0.794627000	-3.521833000	-0.851436000
H	1.104779000	-4.245910000	-0.088714000
H	0.023569000	-2.884794000	-0.417673000
H	0.348093000	-4.077079000	-1.685952000

130

A_TS4 (Full mode) E = -3857.42422333 Imaginary Frequency= 406.4425

Al	-0.290450000	-0.993481000	0.948314000
O	-1.790800000	-1.763973000	0.201120000
O	1.020937000	-2.193296000	0.455948000
N	-1.414547000	0.596026000	1.651938000
N	1.170688000	0.267354000	1.695310000
C	-2.911343000	-1.209259000	-0.173580000
C	2.248687000	-1.964209000	0.062471000
C	2.908962000	-2.884888000	-0.811861000
C	-3.682538000	-1.800474000	-1.232263000
C	-0.770983000	1.294987000	2.608733000
C	-2.760504000	0.644625000	1.553481000
C	0.697865000	1.169866000	2.580855000
C	-4.882280000	-1.207960000	-1.595102000
C	2.499523000	0.085344000	1.539154000
C	-4.659012000	0.516804000	0.017458000
C	-3.438981000	-0.038832000	0.456971000
C	-5.400794000	-0.040482000	-1.004857000
C	-1.444559000	2.069681000	3.544337000
C	2.998177000	-0.834306000	0.520478000
C	1.542337000	1.934449000	3.376813000
C	-3.492784000	1.378319000	2.513544000
C	-2.836374000	2.082076000	3.501914000

C	4.145598000	-2.529893000	-1.344135000
C	2.915966000	1.722332000	3.272972000
C	4.252476000	-0.548633000	-0.047740000
C	3.396023000	0.802701000	2.361840000
C	4.825919000	-1.344309000	-1.024028000
H	-5.450814000	-1.652294000	-2.407715000
H	-4.987315000	1.446491000	0.473446000
H	4.602952000	-3.202499000	-2.061039000
H	4.751661000	0.375057000	0.240872000
H	4.459569000	0.608690000	2.284855000
H	3.605086000	2.279128000	3.902320000
H	1.143995000	2.672656000	4.063653000
H	-0.904904000	2.617791000	4.308175000
H	-3.401901000	2.638831000	4.244054000
H	-4.575784000	1.358308000	2.483110000
C	-0.228825000	-2.779830000	3.281205000
C	1.267862000	-2.916967000	3.543995000
O	-0.568746000	-1.595066000	2.652002000
H	-0.738621000	-2.817872000	4.264654000
H	1.494412000	-3.812637000	4.132317000
C	-0.808219000	-3.915438000	2.442724000
H	-1.882838000	-3.776918000	2.312614000
H	-0.333069000	-3.962805000	1.458789000
Cl	-0.588779000	-5.558737000	3.202333000
H	1.614873000	-2.038966000	4.100621000
H	1.831017000	-2.967509000	2.607184000
C	-0.064145000	1.279668000	-1.109476000
C	-1.199007000	1.799218000	-1.946641000
O	-0.137427000	-0.100630000	-0.807974000
H	0.143632000	1.912188000	-0.229475000
H	-0.966593000	2.801850000	-2.322140000
C	1.109390000	0.731375000	-1.769123000
H	2.014588000	0.534665000	-1.219005000
H	0.984873000	0.272608000	-2.740706000
H	-2.121514000	1.846625000	-1.360125000
H	-1.365439000	1.135678000	-2.801324000
C	0.202794000	5.145904000	-0.377641000
C	-0.486794000	5.817765000	-1.537965000
O	1.332750000	5.967913000	-0.016319000
H	0.535034000	4.145821000	-0.665975000
H	-1.384234000	5.262019000	-1.822353000
C	-0.622716000	5.047636000	0.894435000
H	-0.054140000	4.549986000	1.686542000
H	-0.934905000	6.036608000	1.239193000

Cl	-2.115396000	4.073894000	0.642124000
H	0.201721000	5.841589000	-2.384707000
H	-0.777316000	6.839775000	-1.273527000
C	2.810019000	3.995887000	0.308866000
C	2.576411000	5.461646000	0.183350000
C	2.794922000	3.069443000	-0.657927000
C	2.365212000	3.346519000	-2.080465000
O	2.059387000	2.331800000	-2.793802000
O	2.324864000	4.544096000	-2.425422000
O	3.474572000	6.241495000	0.421250000
H	3.165936000	3.723904000	1.302668000
H	3.110150000	2.056733000	-0.400181000
C	6.104124000	-0.887635000	-1.724686000
C	7.214083000	-0.642415000	-0.693546000
H	8.129451000	-0.295011000	-1.187689000
H	6.926317000	0.121328000	0.036305000
H	7.450997000	-1.558920000	-0.141952000
C	5.813568000	0.423272000	-2.471774000
H	5.011150000	0.293205000	-3.205168000
H	5.494094000	1.216960000	-1.787987000
H	6.707594000	0.777440000	-2.999382000
C	6.608412000	-1.919543000	-2.733355000
H	7.525828000	-1.556118000	-3.208974000
H	6.838626000	-2.878920000	-2.256078000
H	5.876416000	-2.103629000	-3.526852000
C	2.265358000	-4.233442000	-1.148097000
C	2.102832000	-5.045218000	0.146449000
H	3.080497000	-5.290351000	0.577416000
H	1.533049000	-4.497009000	0.897589000
H	1.575400000	-5.985954000	-0.053341000
C	3.130588000	-5.064223000	-2.100221000
H	3.259544000	-4.577866000	-3.074050000
H	4.124301000	-5.270560000	-1.686578000
H	2.641473000	-6.028245000	-2.276741000
C	0.901517000	-4.043618000	-1.821652000
H	0.181491000	-3.568366000	-1.156143000
H	0.992687000	-3.416548000	-2.716516000
H	0.494882000	-5.015115000	-2.131530000
C	-3.131391000	-2.992055000	-2.019214000
C	-2.774345000	-4.173145000	-1.102754000
H	-1.997431000	-3.916813000	-0.384057000
H	-3.656497000	-4.510016000	-0.545141000
H	-2.419839000	-5.016179000	-1.708571000
C	-4.137454000	-3.522983000	-3.045960000

H	-5.067955000	-3.862357000	-2.575278000
H	-4.391015000	-2.777273000	-3.807756000
H	-3.697419000	-4.380661000	-3.566115000
C	-1.894953000	-2.511434000	-2.795622000
H	-1.151083000	-2.060818000	-2.135358000
H	-1.428058000	-3.349215000	-3.328194000
H	-2.186908000	-1.758547000	-3.537926000
C	-6.696350000	0.564678000	-1.538862000
C	-7.101829000	1.824023000	-0.774083000
H	-7.261646000	1.618112000	0.290297000
H	-6.344549000	2.611202000	-0.855865000
H	-8.038440000	2.222452000	-1.179528000
C	-7.833687000	-0.459520000	-1.418356000
H	-8.769294000	-0.048517000	-1.817549000
H	-7.610528000	-1.376920000	-1.972647000
H	-8.002088000	-0.737917000	-0.372496000
C	-6.515759000	0.936964000	-3.017841000
H	-5.710323000	1.668691000	-3.141526000
H	-6.261594000	0.061355000	-3.624134000
H	-7.437700000	1.369966000	-3.425614000

36

B E = -1580.863315

C	-0.541818000	2.753583000	-0.917980000
C	0.713068000	2.883872000	-0.066276000
N	1.333748000	1.572143000	-0.068463000
C	2.615453000	1.445119000	-0.219961000
C	3.297349000	0.194673000	-0.255228000
C	2.577773000	-1.038997000	-0.190294000
C	3.320015000	-2.236380000	-0.278501000
C	4.696263000	-2.211893000	-0.406276000
C	5.405316000	-0.999106000	-0.464949000
C	4.702013000	0.184988000	-0.394644000
N	-1.174216000	1.484999000	-0.567688000
C	-2.466327000	1.374162000	-0.704296000
C	-3.220652000	0.195835000	-0.462655000
C	-2.578656000	-1.037388000	-0.119886000
C	-3.394754000	-2.181740000	0.031944000
C	-4.764633000	-2.100386000	-0.121071000
C	-5.398220000	-0.885837000	-0.447429000
C	-4.625152000	0.240735000	-0.620258000
O	-1.290197000	-1.140363000	0.024534000
Al	-0.001910000	0.109916000	0.313688000
O	1.277113000	-1.101875000	-0.077740000
H	-3.030043000	2.253343000	-1.045152000

H	3.226898000	2.350578000	-0.339012000
H	-5.085180000	1.192122000	-0.882935000
H	-6.475846000	-0.842120000	-0.564061000
H	-5.366416000	-2.995674000	0.014415000
H	5.239520000	-3.151884000	-0.462275000
H	6.485466000	-0.997001000	-0.565575000
H	-2.906378000	-3.117097000	0.284511000
H	2.771477000	-3.171519000	-0.233779000
H	5.222404000	1.140294000	-0.442908000
Cl	-0.201567000	0.578987000	2.417130000
H	-1.225275000	3.598431000	-0.771260000
H	-0.261872000	2.728333000	-1.980212000
H	1.386516000	3.663233000	-0.443515000
H	0.435923000	3.127743000	0.967871000

46

B_INT1' E = -1773.9807903

C	-0.703556000	2.912746000	0.640913000
C	0.559411000	2.518686000	1.399543000
N	1.194335000	1.447914000	0.650420000
C	2.486287000	1.366947000	0.586167000
C	3.199762000	0.318698000	-0.070360000
C	2.528481000	-0.828226000	-0.606673000
C	3.328194000	-1.853728000	-1.157340000
C	4.705481000	-1.740882000	-1.202858000
C	5.363701000	-0.611301000	-0.687209000
C	4.608300000	0.395434000	-0.122850000
N	-1.347218000	1.677414000	0.231035000
C	-2.633087000	1.546605000	0.297043000
C	-3.352848000	0.381911000	-0.107993000
C	-2.692247000	-0.759970000	-0.666348000
C	-3.497618000	-1.862789000	-1.033761000
C	-4.869302000	-1.837125000	-0.865439000
C	-5.517595000	-0.714778000	-0.321676000
C	-4.755665000	0.374691000	0.046819000
O	-1.403292000	-0.833542000	-0.841863000
Al	-0.049690000	0.319530000	-0.465695000
O	1.231044000	-0.981878000	-0.561213000
H	-3.229853000	2.382759000	0.688407000
H	3.091931000	2.140900000	1.079331000
H	-5.229960000	1.259941000	0.468385000
H	-6.595400000	-0.705410000	-0.198272000
H	-5.456326000	-2.702482000	-1.163671000
H	5.288452000	-2.542961000	-1.648945000
H	6.445126000	-0.535760000	-0.729701000

H	-2.996410000	-2.725906000	-1.461034000
H	2.819060000	-2.723413000	-1.560541000
H	5.091218000	1.277735000	0.294725000
H	-1.367017000	3.540741000	1.248595000
H	-0.425824000	3.465965000	-0.266003000
H	1.233132000	3.372349000	1.546650000
H	0.280759000	2.130465000	2.390475000
Cl	0.319140000	1.415967000	-2.339887000
C	0.111099000	-1.258572000	2.792935000
C	1.601804000	-1.167562000	2.851527000
O	-0.563069000	-0.587115000	1.709217000
H	-0.411597000	-1.104254000	3.739171000
H	1.990845000	-1.903230000	3.561791000
C	-0.592862000	-2.020170000	1.765113000
H	-1.580220000	-2.435590000	1.960723000
H	-0.016926000	-2.496190000	0.972251000
H	1.927474000	-0.178757000	3.190475000
H	2.048412000	-1.362940000	1.874667000

37

B_INT1 E = -2041.1619996

C	-0.639510000	2.828821000	-0.415397000
C	0.639679000	2.828966000	0.415277000
N	1.294036000	1.553031000	0.207658000
C	2.579011000	1.461561000	0.189920000
C	3.308208000	0.232869000	0.086883000
C	2.655316000	-1.048774000	0.087701000
C	3.496339000	-2.193211000	0.036996000
C	4.871097000	-2.084136000	-0.024235000
C	5.504082000	-0.828381000	-0.029944000
C	4.714044000	0.303680000	0.030844000
N	-1.293970000	1.553018000	-0.207267000
C	-2.578957000	1.461512000	-0.191058000
C	-3.308247000	0.232869000	-0.088016000
C	-2.655271000	-1.048742000	-0.086549000
C	-3.496292000	-2.193166000	-0.035126000
C	-4.871177000	-2.084116000	0.023302000
C	-5.504272000	-0.828401000	0.025744000
C	-4.714188000	0.303643000	-0.034792000
O	-1.376332000	-1.213311000	-0.146671000
Al	0.000037000	0.001189000	0.001372000
O	1.376408000	-1.213354000	0.148746000
H	-3.181085000	2.380285000	-0.275995000
H	3.181186000	2.380431000	0.273370000
H	-5.173782000	1.292613000	-0.043266000

H	-6.586675000	-0.751138000	0.069820000
H	-5.474324000	-2.989513000	0.067725000
H	5.474229000	-2.989552000	-0.068485000
H	6.586372000	-0.751085000	-0.076761000
H	-3.005766000	-3.161890000	-0.037222000
H	3.005909000	-3.161964000	0.041419000
H	5.173565000	1.292695000	0.037030000
Cl	-0.312190000	0.203910000	2.327262000
H	-1.294233000	3.672834000	-0.154548000
H	-0.378751000	2.893813000	-1.480781000
H	1.294462000	3.672817000	0.154049000
H	0.378937000	2.894436000	1.480649000
Cl	0.312145000	0.202606000	-2.325701000

47

B_TS1 E = -2234.2480531 Imaginary Frequency= 418.5894

C	0.720411000	-2.375840000	2.166877000
C	-0.552771000	-1.636025000	2.572339000
N	-1.198556000	-1.191297000	1.355188000
C	-2.479202000	-1.270858000	1.208290000
C	-3.192079000	-0.820567000	0.052605000
C	-2.544996000	-0.115294000	-1.018930000
C	-3.366910000	0.346405000	-2.076095000
C	-4.727136000	0.108522000	-2.089817000
C	-5.356752000	-0.592472000	-1.046169000
C	-4.584966000	-1.036644000	0.008468000
N	1.376743000	-1.580561000	1.149064000
C	2.663953000	-1.505749000	1.090292000
C	3.395699000	-0.801817000	0.082378000
C	2.749869000	-0.164076000	-1.032869000
C	3.589598000	0.485718000	-1.975079000
C	4.961988000	0.507923000	-1.828805000
C	5.590705000	-0.121371000	-0.739270000
C	4.801335000	-0.765328000	0.192378000
O	1.470744000	-0.160472000	-1.226340000
Al	0.092347000	-0.665668000	-0.120051000
O	-1.274595000	0.154327000	-1.039548000
H	3.262833000	-2.020500000	1.857988000
H	-3.084665000	-1.703050000	2.020374000
H	5.258214000	-1.264071000	1.047454000
H	6.671011000	-0.099553000	-0.633687000
H	5.566257000	1.024424000	-2.571913000
H	-5.321065000	0.474699000	-2.924692000
H	-6.427366000	-0.771668000	-1.065854000
H	3.102702000	0.972007000	-2.814818000

H	-2.879993000	0.892221000	-2.878037000
H	-5.045365000	-1.570779000	0.839639000
H	1.375319000	-2.563375000	3.029526000
H	0.445081000	-3.337707000	1.712886000
H	-1.213636000	-2.267248000	3.183313000
H	-0.282604000	-0.747882000	3.162900000
Cl	-0.262401000	-2.732142000	-1.091065000
C	-0.134692000	2.047771000	1.553911000
C	-1.625097000	2.032701000	1.716855000
O	0.532870000	0.922068000	0.970035000
H	0.385079000	2.368498000	2.464011000
H	-1.943211000	3.060350000	1.917907000
C	0.449343000	2.519480000	0.321290000
H	1.471870000	2.861567000	0.287531000
H	-0.117150000	2.430862000	-0.595900000
Cl	-0.184240000	5.017220000	0.388670000
H	-1.939410000	1.388696000	2.545596000
H	-2.120788000	1.700638000	0.802323000

47

B_INT2 E = -2234.2810668

C	-0.578223000	-2.075879000	2.337499000
C	0.688472000	-2.737967000	1.797089000
N	1.356302000	-1.773909000	0.949110000
C	2.640763000	-1.708729000	0.890277000
C	3.378350000	-0.841535000	0.018236000
C	2.741379000	-0.013778000	-0.973878000
C	3.599537000	0.771620000	-1.792724000
C	4.972031000	0.749233000	-1.646305000
C	5.587967000	-0.063417000	-0.677843000
C	4.782914000	-0.844338000	0.129676000
N	-1.234345000	-1.425072000	1.224797000
C	-2.513049000	-1.456500000	1.083380000
C	-3.225738000	-0.797632000	0.025931000
C	-2.574563000	0.072637000	-0.915462000
C	-3.401311000	0.704468000	-1.880027000
C	-4.764457000	0.484604000	-1.927386000
C	-5.395615000	-0.371093000	-1.008962000
C	-4.618807000	-0.989523000	-0.047255000
O	-1.306467000	0.340112000	-0.900188000
Al	0.091280000	-0.557587000	-0.067123000
O	1.467195000	0.043059000	-1.167142000
H	-3.124387000	-2.013721000	1.812206000
H	3.240564000	-2.368297000	1.538856000
H	-5.079739000	-1.652395000	0.685730000

H	-5.357247000	0.985097000	-2.691028000
H	5.586005000	1.372207000	-2.294640000
H	5.230305000	-1.489902000	0.886509000
H	-1.234756000	-2.803323000	2.837986000
H	-0.294545000	-1.296797000	3.058690000
H	1.339674000	-3.093752000	2.609117000
H	0.403482000	-3.591582000	1.166628000
C	0.021488000	1.938604000	1.533634000
C	-1.440632000	1.970716000	1.972624000
O	0.484914000	0.670038000	1.255399000
H	0.623356000	2.372727000	2.359177000
H	-1.753447000	2.979801000	2.263817000
C	0.277367000	2.804593000	0.302767000
H	1.323016000	2.731597000	-0.000022000
H	-0.367724000	2.513814000	-0.529686000
Cl	-0.035725000	4.584435000	0.608305000
H	-1.579683000	1.303322000	2.830364000
H	-2.098129000	1.626041000	1.167568000
H	-6.467805000	-0.538647000	-1.051034000
H	6.668484000	-0.078841000	-0.569676000
H	3.122357000	1.396432000	-2.542547000
Cl	-0.290205000	-2.471461000	-1.439206000
H	-2.911698000	1.364985000	-2.589605000

46

B_INT3 E = -1773.9832678

C	0.448372000	-2.516069000	-2.039565000
C	-0.899565000	-2.990288000	-1.513659000
N	-1.467566000	-1.900438000	-0.728435000
C	-2.762487000	-1.824814000	-0.604095000
C	-3.455447000	-0.873280000	0.191502000
C	-2.748420000	0.041950000	1.037118000
C	-3.515687000	0.907149000	1.851747000
C	-4.895755000	0.882109000	1.812147000
C	-5.590914000	-0.012304000	0.975843000
C	-4.869197000	-0.879472000	0.185411000
N	1.082318000	-1.798756000	-0.948545000
C	2.339347000	-1.975029000	-0.683449000
C	3.042398000	-1.294050000	0.353122000
C	2.377258000	-0.355886000	1.203793000
C	3.144386000	0.280456000	2.203932000
C	4.491283000	0.005040000	2.350631000
C	5.144798000	-0.919280000	1.516989000
C	4.417559000	-1.556517000	0.533528000
O	1.107174000	-0.070011000	1.094290000

Al	-0.195004000	-0.478698000	-0.106750000
O	-1.450005000	0.082699000	1.097777000
H	2.915715000	-2.686871000	-1.291695000
H	-3.381910000	-2.558904000	-1.137999000
H	4.896841000	-2.275222000	-0.129491000
H	6.202481000	-1.123895000	1.644558000
H	5.054760000	0.516338000	3.127040000
H	-5.456365000	1.568365000	2.442127000
H	-6.675584000	-0.019444000	0.959541000
H	2.639062000	0.993921000	2.847054000
H	-2.979493000	1.594813000	2.498025000
H	-5.379388000	-1.589218000	-0.464108000
H	1.062938000	-3.348919000	-2.403850000
H	0.292469000	-1.806870000	-2.863180000
H	-1.569672000	-3.296149000	-2.326278000
H	-0.750298000	-3.859552000	-0.858117000
C	0.363106000	1.793553000	-1.754816000
C	1.840532000	1.533464000	-2.003473000
O	-0.338690000	0.620825000	-1.462753000
H	-0.072294000	2.240825000	-2.664254000
H	2.366843000	2.446347000	-2.296952000
C	0.127337000	2.764332000	-0.602382000
H	-0.942776000	2.888506000	-0.425326000
H	0.603395000	2.402928000	0.315475000
Cl	0.798784000	4.409392000	-0.933407000
H	1.961155000	0.792614000	-2.800076000
H	2.323351000	1.143669000	-1.098406000

56

B_INT4 E = -1967.1044395

C	-0.509538000	-0.992711000	3.073020000
C	0.702280000	-1.857547000	2.732892000
N	1.351227000	-1.264330000	1.576329000
C	2.637512000	-1.323043000	1.437608000
C	3.357063000	-0.829059000	0.304984000
C	2.689931000	-0.322693000	-0.859651000
C	3.497285000	0.091299000	-1.943670000
C	4.876706000	0.025444000	-1.878482000
C	5.530204000	-0.468996000	-0.736783000
C	4.767098000	-0.893378000	0.331351000
N	-1.163674000	-0.671949000	1.818622000
C	-2.446729000	-0.750245000	1.690011000
C	-3.158050000	-0.427385000	0.490001000
C	-2.497983000	0.105782000	-0.665136000
C	-3.300685000	0.426926000	-1.784232000

C	-4.667142000	0.214382000	-1.774276000
C	-5.311663000	-0.321813000	-0.647201000
C	-4.554489000	-0.625846000	0.466665000
O	-1.211400000	0.313515000	-0.730657000
Al	0.160802000	-0.146152000	0.397727000
O	1.390073000	-0.264902000	-0.970736000
H	-3.051550000	-1.076905000	2.548690000
H	3.237221000	-1.793207000	2.230785000
H	-5.029999000	-1.029510000	1.359576000
H	-6.384552000	-0.482697000	-0.648774000
H	-5.250653000	0.470182000	-2.655347000
H	5.464658000	0.364403000	-2.727975000
H	6.613350000	-0.516575000	-0.697954000
H	-2.800451000	0.844323000	-2.653257000
H	2.991763000	0.476119000	-2.824280000
H	5.246410000	-1.285892000	1.227184000
H	-1.185172000	-1.496591000	3.776334000
H	-0.167306000	-0.051200000	3.521948000
H	1.387303000	-1.953649000	3.585061000
H	0.358579000	-2.865706000	2.459029000
C	0.274891000	2.661102000	1.084115000
C	-1.173403000	2.858109000	1.509122000
O	0.697118000	1.339281000	1.209909000
H	0.911246000	3.294257000	1.727826000
H	-1.464103000	3.912275000	1.471312000
C	0.524273000	3.085362000	-0.359774000
H	1.562617000	2.889999000	-0.634875000
H	-0.139495000	2.549467000	-1.044533000
Cl	0.240973000	4.854947000	-0.626580000
H	-1.312250000	2.498627000	2.533854000
H	-1.849764000	2.296096000	0.854851000
C	-0.622306000	-2.462428000	-1.733999000
C	-1.885652000	-3.154643000	-2.130178000
O	-0.447535000	-2.268211000	-0.309396000
H	-0.345649000	-1.567680000	-2.292630000
H	-1.828837000	-3.484220000	-3.171665000
C	0.454283000	-3.135058000	-1.013531000
H	1.472482000	-2.758402000	-1.091908000
H	0.345446000	-4.186196000	-0.746864000
H	-2.741504000	-2.478032000	-2.040753000
H	-2.068984000	-4.028280000	-1.498971000

57

B_TS2 E = -2427.3719402 Imaginary Frequency= 403.3567

C	-0.711197000	0.010961000	3.282220000

C	0.688865000	-0.600206000	3.198942000
N	1.294060000	-0.139643000	1.967756000
C	2.547362000	0.162197000	1.906347000
C	3.231996000	0.568686000	0.715546000
C	2.604315000	0.559444000	-0.578354000
C	3.406905000	0.935503000	-1.684906000
C	4.728454000	1.304782000	-1.529978000
C	5.338676000	1.315205000	-0.263647000
C	4.587250000	0.941281000	0.832228000
N	-1.326951000	-0.151365000	1.983250000
C	-2.546948000	-0.542585000	1.854405000
C	-3.215702000	-0.707656000	0.594816000
C	-2.615810000	-0.327918000	-0.655376000
C	-3.401145000	-0.499140000	-1.823799000
C	-4.673539000	-1.035032000	-1.772272000
C	-5.251130000	-1.419218000	-0.550599000
C	-4.520044000	-1.241001000	0.608270000
O	-1.425769000	0.180234000	-0.766443000
Al	-0.015394000	0.161391000	0.450586000
O	1.373426000	0.200712000	-0.781473000
H	-3.140936000	-0.765882000	2.755534000
H	3.157879000	0.100318000	2.821295000
H	-4.946091000	-1.519543000	1.572237000
H	-6.250792000	-1.841248000	-0.517651000
H	-5.234416000	-1.162094000	-2.695932000
H	5.307099000	1.586420000	-2.407287000
H	6.380397000	1.599766000	-0.152478000
H	-2.950552000	-0.205692000	-2.767570000
H	2.937104000	0.916730000	-2.663746000
H	5.036193000	0.926106000	1.825412000
H	-1.307231000	-0.446367000	4.085462000
H	-0.618640000	1.088214000	3.476417000
H	1.295790000	-0.346412000	4.080021000
H	0.597928000	-1.694680000	3.146376000
C	-0.603503000	3.046477000	0.431301000
C	-2.090309000	3.026461000	0.773482000
O	0.077515000	1.929878000	0.878929000
H	-0.165464000	3.948036000	0.904858000
H	-2.583797000	3.959507000	0.480777000
C	-0.352785000	3.170141000	-1.069036000
H	0.718599000	3.150483000	-1.275334000
H	-0.848090000	2.367797000	-1.621274000
Cl	-0.973908000	4.742408000	-1.760999000
H	-2.216837000	2.891709000	1.853158000

H	-2.596495000	2.197847000	0.267005000
C	0.018421000	-2.566401000	-0.846869000
C	-1.030969000	-3.617961000	-1.046169000
O	-0.098887000	-1.815318000	0.370411000
H	0.181502000	-1.926164000	-1.719773000
H	-0.704695000	-4.305036000	-1.832158000
C	1.207676000	-2.868660000	-0.081400000
H	2.085657000	-2.246983000	-0.179173000
H	1.173899000	-3.638441000	0.674170000
Cl	2.432205000	-4.682788000	-1.389299000
H	-1.990973000	-3.171095000	-1.326424000
H	-1.169036000	-4.194044000	-0.125833000

57

B_INT5 E = -2427.4000556

C	-0.743719000	0.387440000	3.303467000
C	0.551000000	-0.430153000	3.311655000
N	1.244851000	-0.167787000	2.071777000
C	2.524339000	-0.040844000	2.027774000
C	3.286160000	0.168281000	0.828228000
C	2.695254000	0.149194000	-0.486188000
C	3.580545000	0.330925000	-1.583798000
C	4.936512000	0.524533000	-1.407104000
C	5.506859000	0.542508000	-0.122631000
C	4.675483000	0.358694000	0.966177000
N	-1.364238000	0.188250000	2.015371000
C	-2.621619000	-0.044987000	1.888145000
C	-3.283962000	-0.248888000	0.628262000
C	-2.627941000	-0.056945000	-0.639926000
C	-3.415219000	-0.267704000	-1.803214000
C	-4.738942000	-0.656287000	-1.730778000
C	-5.373168000	-0.844685000	-0.491413000
C	-4.639880000	-0.628821000	0.660728000
O	-1.397812000	0.327265000	-0.773351000
Al	-0.007860000	0.095549000	0.476756000
O	1.440055000	-0.053735000	-0.718073000
H	-3.259690000	-0.095811000	2.787014000
H	3.104826000	-0.102471000	2.964077000
H	-5.107105000	-0.759203000	1.637512000
H	-6.414578000	-1.148227000	-0.439691000
H	-5.297045000	-0.818434000	-2.651219000
H	5.572091000	0.662613000	-2.280064000
H	6.574268000	0.691836000	0.010413000
H	-2.922127000	-0.123693000	-2.760563000
H	3.138872000	0.311847000	-2.576250000

H	5.089298000	0.359782000	1.975331000
H	-1.406011000	0.104374000	4.136225000
H	-0.491035000	1.452858000	3.394371000
H	1.170308000	-0.202040000	4.192788000
H	0.291731000	-1.498107000	3.329780000
C	-0.163011000	3.057266000	0.240477000
C	-1.638381000	3.288160000	0.560959000
O	0.343677000	1.890541000	0.771377000
H	0.401285000	3.919963000	0.653245000
H	-1.984645000	4.259409000	0.189862000
C	0.109494000	3.035893000	-1.261470000
H	1.166818000	2.843440000	-1.450121000
H	-0.499513000	2.282078000	-1.765283000
Cl	-0.262574000	4.635663000	-2.076556000
H	-1.785916000	3.259945000	1.646305000
H	-2.260937000	2.505930000	0.114976000
C	-0.269699000	-2.598535000	-0.517171000
C	-1.432494000	-3.581465000	-0.488684000
O	-0.333580000	-1.735385000	0.561847000
H	-0.286319000	-2.063417000	-1.488714000
H	-1.394077000	-4.293990000	-1.320019000
C	1.081892000	-3.293991000	-0.395604000
H	1.889870000	-2.562457000	-0.433932000
H	1.134341000	-3.860892000	0.538642000
Cl	1.432943000	-4.498125000	-1.734633000
H	-2.379039000	-3.033378000	-0.538592000
H	-1.419807000	-4.142508000	0.454508000

66

B_TS3 E= -2806.6611585 Imaginary Frequency= 177.6980

C	-0.303581000	-1.131283000	3.083311000
C	-0.258923000	0.389111000	3.255412000
N	-0.716662000	1.010187000	2.018672000
C	-1.200796000	2.209298000	2.062538000
C	-1.734086000	2.934036000	0.952461000
C	-1.817331000	2.372532000	-0.367004000
C	-2.388609000	3.186930000	-1.380668000
C	-2.839757000	4.463881000	-1.112939000
C	-2.756382000	5.011312000	0.180380000
C	-2.210029000	4.241818000	1.187359000
N	0.014775000	-1.450178000	1.705484000
C	0.741726000	-2.481178000	1.408994000
C	1.002934000	-2.911199000	0.073148000
C	0.292250000	-2.360446000	-1.046426000
C	0.599088000	-2.878430000	-2.327580000

C	1.553092000	-3.860848000	-2.491573000
C	2.247091000	-4.403217000	-1.392284000
C	1.960473000	-3.930181000	-0.129756000
O	-0.637921000	-1.461837000	-0.924008000
Al	-0.867879000	-0.213302000	0.390040000
O	-1.384978000	1.195426000	-0.678558000
H	1.191697000	-3.069419000	2.221346000
H	-1.228017000	2.734173000	3.030289000
H	2.485722000	-4.319944000	0.741887000
H	2.998938000	-5.172619000	-1.539162000
H	1.782055000	-4.214337000	-3.494699000
H	-3.267132000	5.055874000	-1.919680000
H	-3.113871000	6.016501000	0.380685000
H	0.087249000	-2.433603000	-3.173942000
H	-2.448866000	2.761174000	-2.378274000
H	-2.134077000	4.637445000	2.200237000
H	0.378435000	-1.630614000	3.785787000
H	-1.327144000	-1.480134000	3.272590000
H	-0.875111000	0.701800000	4.108571000
H	0.771511000	0.711869000	3.464369000
C	-3.442276000	-1.577461000	0.624802000
C	-2.944188000	-3.004764000	0.826234000
O	-2.524288000	-0.615016000	1.015017000
H	-4.364822000	-1.454768000	1.226041000
H	-3.714643000	-3.739563000	0.569287000
C	-3.816018000	-1.290609000	-0.826753000
H	-4.121172000	-0.248525000	-0.937176000
H	-2.982124000	-1.501489000	-1.500830000
Cl	-5.225095000	-2.297925000	-1.400727000
H	-2.663111000	-3.154614000	1.874525000
H	-2.062450000	-3.197347000	0.206612000
C	2.185030000	0.592405000	0.917846000
C	2.592488000	1.998559000	1.322076000
O	1.225209000	0.495452000	-0.134479000
H	1.647706000	0.170173000	1.766215000
H	3.085218000	1.952245000	2.301012000
C	3.377082000	-0.343482000	0.696009000
H	3.132181000	-1.132287000	-0.016919000
H	4.291074000	0.159729000	0.388669000
Cl	3.787673000	-1.187997000	2.276431000
H	1.711308000	2.642085000	1.408542000
H	3.293629000	2.462571000	0.627303000
C	1.299635000	2.439924000	-1.742041000
C	1.526945000	0.965703000	-1.615037000

C	2.417673000	3.150842000	-1.885702000
C	3.626788000	2.295345000	-1.746188000
O	4.782940000	2.715530000	-1.786790000
O	3.280588000	1.045837000	-1.536020000
O	1.138521000	0.170030000	-2.444609000
H	0.280372000	2.805736000	-1.811113000
H	2.494780000	4.216460000	-2.072858000

66

B_INT6 E =-2806.7000981

C	-0.776988000	-0.715487000	2.955591000
C	0.621691000	-0.239923000	2.566613000
N	0.488952000	0.609095000	1.402791000
C	1.265673000	1.627474000	1.235794000
C	1.268080000	2.476960000	0.085170000
C	0.421734000	2.248736000	-1.056812000
C	0.562216000	3.150882000	-2.145080000
C	1.467991000	4.191904000	-2.116425000
C	2.299468000	4.405204000	-1.002179000
C	2.187642000	3.546546000	0.073585000
N	-1.451582000	-1.114819000	1.739197000
C	-2.201105000	-2.160657000	1.696528000
C	-2.954958000	-2.569818000	0.545857000
C	-3.052075000	-1.756303000	-0.637197000
C	-3.883618000	-2.243945000	-1.679999000
C	-4.551576000	-3.448636000	-1.576870000
C	-4.447235000	-4.242814000	-0.422039000
C	-3.660965000	-3.786767000	0.619069000
O	-2.470873000	-0.608513000	-0.778330000
Al	-1.054485000	0.152323000	0.175010000
O	-0.436387000	1.287129000	-1.148167000
H	-2.303172000	-2.790009000	2.596577000
H	2.006189000	1.865885000	2.015078000
H	-3.571659000	-4.372335000	1.534672000
H	-4.978843000	-5.186624000	-0.346558000
H	-5.171674000	-3.784614000	-2.405927000
H	1.541730000	4.855592000	-2.976157000
H	3.015989000	5.221070000	-0.991271000
H	-3.966853000	-1.627830000	-2.570560000
H	-0.073254000	2.980190000	-3.009346000
H	2.826204000	3.675683000	0.948434000
H	-0.742490000	-1.527171000	3.697746000
H	-1.333947000	0.131228000	3.379168000
H	1.128151000	0.269993000	3.398548000
H	1.232425000	-1.114943000	2.292686000

C	-3.265367000	1.937621000	0.997450000
C	-4.307715000	0.937181000	1.490967000
O	-1.971536000	1.463694000	1.074852000
H	-3.356145000	2.846709000	1.626938000
H	-5.313182000	1.372302000	1.501427000
C	-3.515057000	2.374358000	-0.443692000
H	-2.735662000	3.064421000	-0.770555000
H	-3.551184000	1.515867000	-1.118744000
Cl	-5.100273000	3.266167000	-0.647956000
H	-4.057063000	0.620145000	2.509332000
H	-4.325954000	0.046367000	0.854445000
C	4.817966000	-1.195258000	0.369316000
C	3.956999000	-2.139164000	1.176905000
O	4.915015000	-1.407170000	-1.056193000
H	5.860841000	-1.319282000	0.690539000
H	4.172038000	-1.967297000	2.235694000
C	4.442427000	0.270797000	0.539288000
H	5.068362000	0.903172000	-0.090354000
H	3.390291000	0.443910000	0.304769000
Cl	4.728957000	0.794612000	2.249987000
H	4.178668000	-3.183889000	0.946535000
H	2.893127000	-1.963659000	1.013202000
C	3.011207000	-2.975223000	-1.547996000
C	4.016901000	-1.940193000	-1.929999000
C	1.738072000	-2.764826000	-1.201381000
C	1.230060000	-1.392986000	-0.895175000
O	0.017566000	-1.357052000	-0.477284000
O	2.024625000	-0.443820000	-1.019831000
O	4.217596000	-1.738656000	-3.108432000
H	3.359333000	-3.986790000	-1.755417000
H	1.049954000	-3.600344000	-1.091145000

46

B_INT7 E = -1773.9832677

C	-0.434274000	-2.510045000	2.044477000
C	0.918617000	-2.976779000	1.524709000
N	1.482007000	-1.886493000	0.736501000
C	2.776865000	-1.805782000	0.613155000
C	3.466685000	-0.855006000	-0.185745000
C	2.756379000	0.054161000	-1.035380000
C	3.521079000	0.917493000	-1.854558000
C	4.901201000	0.897851000	-1.814077000
C	5.599283000	0.010344000	-0.972718000
C	4.880500000	-0.855883000	-0.178707000
N	-1.068965000	-1.799739000	0.949354000

C	-2.324031000	-1.984845000	0.680809000
C	-3.028552000	-1.309934000	-0.358733000
C	-2.367144000	-0.368850000	-1.208993000
C	-3.136250000	0.263316000	-2.210298000
C	-4.481300000	-0.019688000	-2.359093000
C	-5.131050000	-0.947717000	-1.526516000
C	-4.401796000	-1.580384000	-0.541323000
O	-1.098617000	-0.076766000	-1.098253000
Al	0.203041000	-0.474164000	0.107642000
O	1.457911000	0.091221000	-1.095616000
H	-2.897593000	-2.699108000	1.288987000
H	3.398627000	-2.535237000	1.150617000
H	-4.878317000	-2.301382000	0.121125000
H	-6.187278000	-1.158646000	-1.656121000
H	-5.046502000	0.488451000	-3.136487000
H	5.459598000	1.582817000	-2.447262000
H	6.683983000	0.007789000	-0.955261000
H	-2.633829000	0.979414000	-2.852682000
H	2.982800000	1.600004000	-2.504547000
H	5.392818000	-1.560798000	0.474374000
H	-1.044403000	-3.346020000	2.408745000
H	-0.286011000	-1.797635000	2.866586000
H	1.588318000	-3.274463000	2.340798000
H	0.777642000	-3.849952000	0.872502000
C	-0.384315000	1.792895000	1.753216000
C	-1.857052000	1.510169000	2.005638000
O	0.334769000	0.630998000	1.460154000
H	0.046359000	2.247324000	2.661397000
H	-2.396484000	2.414847000	2.300026000
C	-0.166699000	2.767128000	0.600021000
H	0.900789000	2.907578000	0.419830000
H	-0.640141000	2.398821000	-0.316426000
Cl	-0.861900000	4.401706000	0.933691000
H	-1.964273000	0.768044000	2.803000000
H	-2.335993000	1.112628000	1.102151000

56

B_INT8 E = -1967.1008537

C	-0.553043000	-1.306932000	2.912401000
C	0.780857000	-1.955155000	2.552164000
N	1.368699000	-1.167274000	1.483425000
C	2.651791000	-0.999244000	1.416792000
C	3.324971000	-0.300856000	0.368388000
C	2.630204000	0.167255000	-0.795734000
C	3.399992000	0.780252000	-1.810438000

C	4.766516000	0.941846000	-1.675797000
C	5.446125000	0.487895000	-0.532343000
C	4.722992000	-0.131218000	0.466075000
N	-1.205250000	-0.969474000	1.660906000
C	-2.468079000	-1.186771000	1.488162000
C	-3.188241000	-0.861182000	0.296776000
C	-2.560845000	-0.206521000	-0.811908000
C	-3.365497000	0.086743000	-1.936159000
C	-4.707000000	-0.247768000	-1.966033000
C	-5.322161000	-0.890174000	-0.878649000
C	-4.560262000	-1.184049000	0.234081000
O	-1.300922000	0.135142000	-0.829418000
Al	0.069507000	-0.136898000	0.348817000
O	1.345397000	0.012365000	-0.970416000
H	-3.046236000	-1.645173000	2.303692000
H	3.286221000	-1.433748000	2.203259000
H	-5.011422000	-1.675722000	1.094885000
H	-6.376395000	-1.144065000	-0.911561000
H	-5.294818000	-0.003941000	-2.847613000
H	5.323706000	1.430487000	-2.471379000
H	6.518992000	0.619482000	-0.439642000
H	-2.890516000	0.590129000	-2.772989000
H	2.874299000	1.132729000	-2.692786000
H	5.224275000	-0.501289000	1.359399000
H	-1.171225000	-1.963579000	3.538006000
H	-0.365618000	-0.372654000	3.457860000
H	1.447100000	-2.032991000	3.420924000
H	0.596528000	-2.971674000	2.174012000
C	-0.282651000	2.596634000	1.204870000
C	-1.755726000	2.531311000	1.581295000
O	0.342307000	1.352397000	1.269626000
H	0.223853000	3.279105000	1.910105000
H	-2.212467000	3.525436000	1.596145000
C	-0.061449000	3.146770000	-0.200651000
H	1.002763000	3.138886000	-0.444356000
H	-0.607232000	2.555127000	-0.941826000
Cl	-0.621396000	4.860112000	-0.375180000
H	-1.865742000	2.085514000	2.575152000
H	-2.312472000	1.913906000	0.866460000
C	0.474614000	-3.416965000	-1.247947000
C	1.962208000	-3.365903000	-1.110550000
O	-0.293126000	-2.387635000	-0.594785000
H	0.018060000	-4.400179000	-1.114229000
H	2.427860000	-4.035373000	-1.839984000

C	-0.278325000	-2.438863000	-2.027484000
H	-1.236042000	-2.705311000	-2.472500000
H	0.252731000	-1.605267000	-2.485793000
H	2.281020000	-3.690544000	-0.114774000
H	2.337580000	-2.355064000	-1.281288000

76

B_TS4 E = -2999.7771512 Imaginary Frequency=436.3206

C	-2.329087000	-0.459242000	3.299330000
C	-1.168565000	0.531533000	3.373589000
N	-1.126456000	1.255051000	2.119397000
C	-0.733997000	2.482929000	2.072471000
C	-0.572634000	3.244963000	0.869906000
C	-0.731765000	2.666486000	-0.436511000
C	-0.478760000	3.510296000	-1.549329000
C	-0.104362000	4.830073000	-1.389014000
C	0.047762000	5.392987000	-0.110096000
C	-0.181376000	4.593742000	0.992723000
N	-2.253950000	-1.112483000	2.009768000
C	-2.478705000	-2.373829000	1.874866000
C	-2.468154000	-3.069087000	0.619697000
C	-2.326376000	-2.388183000	-0.638814000
C	-2.349833000	-3.187961000	-1.809064000
C	-2.487434000	-4.561314000	-1.747423000
C	-2.627013000	-5.223582000	-0.516364000
C	-2.622104000	-4.470320000	0.641286000
O	-2.196357000	-1.101297000	-0.758411000
Al	-1.758067000	0.176835000	0.517713000
O	-1.060786000	1.431871000	-0.647638000
H	-2.705658000	-2.979610000	2.767086000
H	-0.481469000	3.000578000	3.011583000
H	-2.730908000	-4.955702000	1.611210000
H	-2.733124000	-6.303011000	-0.476376000
H	-2.483599000	-5.136966000	-2.670320000
H	0.080296000	5.440740000	-2.270481000
H	0.350125000	6.428931000	0.007533000
H	-2.238095000	-2.674852000	-2.759839000
H	-0.591500000	3.070545000	-2.536509000
H	-0.054541000	4.997598000	1.997302000
H	-2.304863000	-1.178394000	4.130833000
H	-3.276502000	0.095413000	3.335795000
H	-1.264047000	1.205388000	4.236898000
H	-0.228051000	-0.028150000	3.473515000
C	-4.579093000	0.906720000	0.089219000
C	-5.265515000	-0.434721000	0.328461000

O	-3.360870000	1.020137000	0.732793000
H	-5.258235000	1.699240000	0.462786000
H	-6.262009000	-0.465436000	-0.125147000
C	-4.348430000	1.186070000	-1.393575000
H	-3.827053000	2.136082000	-1.522338000
H	-3.771278000	0.385369000	-1.862972000
Cl	-5.906639000	1.329147000	-2.334363000
H	-5.370082000	-0.606851000	1.405298000
H	-4.673835000	-1.255885000	-0.090004000
C	1.224654000	-0.726805000	-0.001718000
C	1.210524000	-0.300692000	-1.446244000
O	0.031761000	-0.669324000	0.755096000
H	2.068283000	-0.282413000	0.550074000
H	2.071822000	-0.753263000	-1.952729000
C	0.986624000	-2.134788000	0.280174000
H	1.218162000	-2.542923000	1.253635000
H	0.317261000	-2.684093000	-0.369029000
H	1.264387000	0.786217000	-1.541909000
H	0.294532000	-0.643405000	-1.936859000
C	4.698054000	0.686042000	-0.783089000
C	4.598468000	1.199618000	-2.196544000
O	6.013037000	0.103695000	-0.639244000
H	3.938018000	-0.079955000	-0.616482000
H	3.630748000	1.682943000	-2.355781000
C	4.599632000	1.756904000	0.290546000
H	4.739118000	1.316538000	1.281505000
H	5.348193000	2.538471000	0.135125000
Cl	2.986915000	2.553336000	0.300257000
H	4.691661000	0.361605000	-2.890090000
H	5.392702000	1.924131000	-2.404134000
C	5.169234000	-1.426247000	1.114186000
C	6.217635000	-0.985168000	0.147168000
C	4.079441000	-2.158874000	0.855228000
C	3.675669000	-2.574061000	-0.537785000
O	2.547925000	-3.168514000	-0.653916000
O	4.470664000	-2.293599000	-1.456243000
O	7.327498000	-1.472225000	0.176433000
H	5.435443000	-1.198675000	2.146551000
H	3.457146000	-2.486140000	1.689005000

76

B_INT9 E = -2999.8151967

C	-2.192477000	-0.596256000	3.302493000
C	-1.043462000	0.412613000	3.369998000
N	-1.049652000	1.169179000	2.138536000

C	-0.804119000	2.433088000	2.128361000
C	-0.745532000	3.248785000	0.949440000
C	-0.865331000	2.705500000	-0.378772000
C	-0.729074000	3.621539000	-1.457577000
C	-0.500686000	4.967591000	-1.248955000
C	-0.387919000	5.492599000	0.049893000
C	-0.507327000	4.626752000	1.120575000
N	-2.126150000	-1.228731000	2.006254000
C	-2.262769000	-2.497656000	1.856632000
C	-2.209779000	-3.170338000	0.586478000
C	-2.178789000	-2.462939000	-0.667902000
C	-2.152343000	-3.255848000	-1.845963000
C	-2.136608000	-4.637107000	-1.798911000
C	-2.165513000	-5.321319000	-0.572618000
C	-2.213345000	-4.578727000	0.593091000
O	-2.204839000	-1.171650000	-0.776823000
Al	-1.618933000	0.081132000	0.503461000
O	-1.055975000	1.454811000	-0.639148000
H	-2.431106000	-3.136105000	2.740631000
H	-0.609752000	2.950485000	3.082937000
H	-2.246279000	-5.082057000	1.559984000
H	-2.155669000	-6.406589000	-0.541017000
H	-2.101447000	-5.200640000	-2.729263000
H	-0.404334000	5.629964000	-2.107361000
H	-0.205110000	6.551241000	0.207726000
H	-2.133050000	-2.725527000	-2.794199000
H	-0.813621000	3.212834000	-2.461110000
H	-0.412498000	5.002549000	2.139950000
H	-2.142521000	-1.324285000	4.126749000
H	-3.145328000	-0.053043000	3.361505000
H	-1.123155000	1.059764000	4.256654000
H	-0.093801000	-0.138544000	3.422693000
C	-4.491818000	0.788032000	0.190889000
C	-5.164734000	-0.562632000	0.426762000
O	-3.254395000	0.901696000	0.788950000
H	-5.166898000	1.567352000	0.602441000
H	-6.177518000	-0.587083000	0.009150000
C	-4.318550000	1.099994000	-1.293482000
H	-3.815000000	2.059480000	-1.421285000
H	-3.752121000	0.315529000	-1.800531000
Cl	-5.913745000	1.246520000	-2.181543000
H	-5.229909000	-0.755824000	1.503305000
H	-4.584166000	-1.371498000	-0.028206000
C	1.094530000	-1.034041000	-0.196376000

C	0.926622000	-0.662473000	-1.667742000
O	0.045506000	-0.755669000	0.628119000
H	2.015665000	-0.501031000	0.159388000
H	1.804076000	-0.973686000	-2.249886000
C	1.408402000	-2.530006000	-0.052070000
H	1.457172000	-2.807212000	1.008747000
H	0.633635000	-3.139662000	-0.527526000
H	0.789220000	0.415744000	-1.773463000
H	0.039201000	-1.151914000	-2.084391000
C	4.614245000	0.820473000	-0.909981000
C	4.681371000	1.202911000	-2.365611000
O	5.929739000	0.318982000	-0.553871000
H	3.860045000	0.042354000	-0.769102000
H	3.720754000	1.613495000	-2.685956000
C	4.313446000	1.974180000	0.032109000
H	4.383330000	1.645622000	1.072879000
H	5.008618000	2.803095000	-0.127723000
Cl	2.650850000	2.590433000	-0.208617000
H	4.904709000	0.321181000	-2.969589000
H	5.460351000	1.952934000	-2.534485000
C	4.959875000	-0.980718000	1.308673000
C	6.109256000	-0.559602000	0.456647000
C	3.933618000	-1.777063000	0.977828000
C	3.800513000	-2.353141000	-0.383618000
O	2.631315000	-2.905287000	-0.738512000
O	4.740968000	-2.365326000	-1.162306000
O	7.234568000	-0.911362000	0.739825000
H	5.052909000	-0.643292000	2.338950000
H	3.194254000	-2.026408000	1.733983000

66

B_INT10 E = -2806.709387

C	-1.241278000	-0.819211000	3.019861000
C	0.078259000	-0.075694000	2.817072000
N	-0.087496000	0.795503000	1.669358000
C	0.441684000	1.970953000	1.645178000
C	0.394548000	2.858260000	0.517048000
C	-0.162033000	2.471814000	-0.749995000
C	-0.078651000	3.419571000	-1.803053000
C	0.515405000	4.655674000	-1.625711000
C	1.069604000	5.023070000	-0.388665000
C	1.004008000	4.119244000	0.656445000
N	-1.671927000	-1.299693000	1.723491000
C	-2.169129000	-2.477024000	1.575108000
C	-2.640932000	-3.007342000	0.326649000

C	-2.683289000	-2.223284000	-0.877318000
C	-3.216585000	-2.851484000	-2.032381000
C	-3.660850000	-4.159766000	-2.010761000
C	-3.610418000	-4.924779000	-0.833805000
C	-3.108736000	-4.335620000	0.311737000
O	-2.306884000	-0.984338000	-0.951516000
Al	-1.293456000	0.033030000	0.223527000
O	-0.677425000	1.306031000	-0.992977000
H	-2.258835000	-3.139750000	2.451162000
H	0.997884000	2.327753000	2.522176000
H	-3.064813000	-4.900200000	1.243384000
H	-3.961924000	-5.952105000	-0.822930000
H	-4.057573000	-4.601387000	-2.923076000
H	0.556207000	5.351134000	-2.462290000
H	1.544659000	5.990796000	-0.257840000
H	-3.257606000	-2.257584000	-2.940934000
H	-0.501145000	3.129419000	-2.761429000
H	1.447115000	4.363042000	1.621766000
H	-1.138755000	-1.635986000	3.749012000
H	-2.001859000	-0.113301000	3.381602000
H	0.377384000	0.481467000	3.715623000
H	0.870494000	-0.800990000	2.578634000
C	-3.930882000	1.303481000	0.619598000
C	-4.782669000	0.092049000	0.988587000
O	-2.590097000	1.137457000	0.900483000
H	-4.317214000	2.168591000	1.196682000
H	-5.850010000	0.286041000	0.835117000
C	-4.051157000	1.670527000	-0.857271000
H	-3.407405000	2.520577000	-1.089035000
H	-3.789268000	0.825197000	-1.498358000
Cl	-5.745811000	2.177279000	-1.328410000
H	-4.623293000	-0.159512000	2.042785000
H	-4.503015000	-0.779827000	0.387712000
C	5.123884000	-1.092407000	-0.007339000
C	5.450724000	-2.381877000	0.701546000
O	4.825863000	-0.112791000	1.014036000
H	4.248056000	-1.229514000	-0.646797000
H	5.716187000	-3.156756000	-0.022103000
C	6.271006000	-0.489261000	-0.799248000
H	5.973141000	0.460190000	-1.249946000
H	7.145582000	-0.327559000	-0.163953000
Cl	6.791098000	-1.560734000	-2.150094000
H	4.571377000	-2.706723000	1.259831000
H	6.289519000	-2.244988000	1.392201000

C	3.079119000	0.866561000	-0.424375000
C	3.752034000	0.710205000	0.898992000
C	2.036984000	0.145230000	-0.852337000
C	1.469536000	-0.978274000	-0.035873000
O	0.211517000	-1.189152000	-0.156189000
O	2.246870000	-1.604659000	0.708226000
O	3.456774000	1.398151000	1.854041000
H	3.405259000	1.745157000	-0.983070000
H	1.516808000	0.426216000	-1.764107000

75

B_TS5 E = -3185.9550574 Imaginary Frequency= 137.0481

C	-0.337405000	-1.749971000	2.466857000
C	0.815461000	-0.749057000	2.403844000
N	0.549770000	0.177040000	1.317103000
C	0.835360000	1.436925000	1.436486000
C	0.683520000	2.390648000	0.387371000
C	0.356248000	1.983556000	-0.945849000
C	0.229131000	2.990823000	-1.928951000
C	0.418640000	4.320663000	-1.608515000
C	0.763801000	4.720223000	-0.303848000
C	0.893538000	3.755886000	0.674361000
N	-0.857995000	-1.963844000	1.119639000
C	-1.788027000	-2.853264000	0.948727000
C	-2.468485000	-3.108644000	-0.277080000
C	-2.071272000	-2.467334000	-1.497158000
C	-2.825681000	-2.754918000	-2.663212000
C	-3.902202000	-3.617020000	-2.617698000
C	-4.287872000	-4.251668000	-1.419811000
C	-3.567211000	-3.995400000	-0.271768000
O	-1.056706000	-1.674473000	-1.570309000
Al	-0.179506000	-0.723025000	-0.309368000
O	0.226710000	0.735995000	-1.288158000
H	-2.119885000	-3.443490000	1.816819000
H	1.217435000	1.805266000	2.396624000
H	-3.845690000	-4.464439000	0.671998000
H	-5.139556000	-4.924511000	-1.401338000
H	-4.471425000	-3.802649000	-3.526267000
H	0.286271000	5.075584000	-2.380455000
H	0.910938000	5.770171000	-0.070142000
H	-2.538609000	-2.241775000	-3.575123000
H	-0.068331000	2.675500000	-2.923613000
H	1.157407000	4.031395000	1.694973000
H	-0.009251000	-2.694671000	2.917156000
H	-1.145934000	-1.350559000	3.099340000

H	0.945592000	-0.226477000	3.360648000
H	1.754366000	-1.275382000	2.184342000
C	6.229493000	-0.687060000	0.246062000
C	6.794810000	-1.956724000	0.829530000
O	5.587855000	0.023683000	1.332848000
H	5.485287000	-0.924526000	-0.519160000
H	7.318761000	-2.527691000	0.059102000
C	7.265719000	0.278824000	-0.301520000
H	6.791420000	1.194222000	-0.662723000
H	8.005926000	0.534629000	0.460829000
Cl	8.159739000	-0.424467000	-1.694861000
H	5.976550000	-2.562587000	1.221476000
H	7.497617000	-1.733562000	1.638862000
C	3.852535000	0.821298000	-0.220008000
C	4.375148000	0.602090000	1.161794000
C	3.023606000	-0.011655000	-0.858487000
C	2.620293000	-1.315747000	-0.243592000
O	1.408892000	-1.708788000	-0.463372000
O	3.445942000	-1.923796000	0.451044000
O	3.797491000	1.029112000	2.139930000
H	4.076861000	1.803469000	-0.636893000
H	2.562938000	0.282105000	-1.797125000
C	-3.127329000	0.744383000	1.275674000
C	-2.299043000	1.454308000	2.327461000
O	-2.386035000	0.425750000	0.101819000
H	-3.937854000	1.426259000	1.001367000
H	-2.945357000	1.796603000	3.141711000
C	-3.793797000	-0.522519000	1.797188000
H	-4.284032000	-1.064401000	0.985094000
H	-3.093704000	-1.186842000	2.308063000
Cl	-5.080640000	-0.150227000	3.021806000
H	-1.808927000	2.324261000	1.882757000
H	-1.532494000	0.803013000	2.759147000
C	-4.525924000	0.398478000	-1.264857000
C	-3.110112000	0.900870000	-1.287936000
C	-5.403848000	1.392993000	-1.125299000
C	-4.679900000	2.680308000	-0.934172000
O	-5.206388000	3.775723000	-0.787142000
O	-3.369940000	2.462829000	-0.938313000
O	-2.385782000	0.716281000	-2.253664000
H	-4.724804000	-0.654513000	-1.450753000
H	-6.486521000	1.346059000	-1.161215000

75

B_INT11 E = -3185.997154

C	0.370668000	-1.063080000	-2.129010000
C	0.068253000	0.409014000	-1.856852000
N	-0.253170000	0.556162000	-0.435680000
C	-0.602441000	1.734156000	-0.021996000
C	-0.894867000	2.054831000	1.345826000
C	-0.879095000	1.077064000	2.390384000
C	-1.248800000	1.504830000	3.687481000
C	-1.595432000	2.820901000	3.941188000
C	-1.596093000	3.781135000	2.917601000
C	-1.249475000	3.385180000	1.638045000
N	1.011447000	-1.627600000	-0.955716000
C	2.030798000	-2.408009000	-1.042898000
C	2.640061000	-3.057711000	0.082313000
C	2.031819000	-3.036224000	1.383496000
C	2.674558000	-3.780078000	2.404971000
C	3.847005000	-4.472680000	2.168946000
C	4.443287000	-4.484225000	0.896664000
C	3.826054000	-3.788865000	-0.125852000
O	0.929122000	-2.406804000	1.655817000
Al	0.150274000	-1.017077000	0.749603000
O	-0.586269000	-0.179906000	2.199083000
H	2.470054000	-2.610352000	-2.033075000
H	-0.685863000	2.547957000	-0.744531000
H	4.253729000	-3.794800000	-1.128905000
H	5.362769000	-5.033324000	0.719151000
H	4.314280000	-5.019560000	2.985510000
H	-1.869075000	3.112264000	4.953664000
H	-1.864327000	4.812554000	3.125303000
H	2.211509000	-3.772177000	3.387129000
H	-1.244874000	0.759145000	4.477616000
H	-1.244675000	4.100653000	0.817238000
H	-0.572104000	-1.610025000	-2.269171000
H	0.990119000	-1.188152000	-3.027737000
H	0.932780000	1.048288000	-2.076576000
H	-0.770911000	0.749629000	-2.475393000
C	-5.864214000	-0.592455000	-1.379237000
C	-6.096435000	-1.405463000	-2.627107000
O	-5.091715000	0.565892000	-1.771765000
H	-5.297129000	-1.178185000	-0.651034000
H	-6.705434000	-2.284018000	-2.399940000
C	-7.123663000	-0.031860000	-0.741508000
H	-6.877541000	0.588247000	0.123575000
H	-7.697295000	0.561833000	-1.457682000
Cl	-8.207768000	-1.339564000	-0.146664000

H	-5.133515000	-1.735505000	-3.019811000
H	-6.610096000	-0.810143000	-3.388941000
C	-3.923910000	0.522436000	0.398916000
C	-4.067591000	1.013689000	-1.003775000
C	-3.195236000	-0.532560000	0.779285000
C	-2.489648000	-1.402333000	-0.212616000
O	-1.387030000	-1.942405000	0.195321000
O	-2.976209000	-1.545638000	-1.341970000
O	-3.361621000	1.896561000	-1.440321000
H	-4.338992000	1.196966000	1.148890000
H	-3.019863000	-0.722819000	1.834640000
C	4.206677000	1.036759000	-1.250130000
C	3.864099000	1.267529000	-2.698044000
O	3.002528000	0.529501000	-0.603092000
H	4.538697000	1.965292000	-0.777183000
H	4.750547000	1.605789000	-3.238804000
C	5.216202000	-0.075258000	-1.005576000
H	5.361426000	-0.247184000	0.063748000
H	4.887502000	-1.008661000	-1.471060000
Cl	6.823645000	0.329503000	-1.697013000
H	3.096996000	2.042079000	-2.775422000
H	3.503218000	0.337952000	-3.155772000
C	3.056453000	2.090796000	1.294899000
C	2.639789000	0.854457000	0.642448000
C	3.005567000	3.310571000	0.717591000
C	2.739826000	3.612221000	-0.737278000
O	3.702900000	4.199027000	-1.287501000
O	1.648049000	3.224972000	-1.220245000
O	1.889250000	0.064214000	1.229526000
H	3.176177000	1.991669000	2.370791000
H	3.193153000	4.175812000	1.353367000

75

B_INT12’ E = -3186.0137184

C	-0.918102000	1.746169000	2.853667000
C	-1.028187000	0.222553000	2.814110000
N	-0.252234000	-0.270065000	1.680075000
C	-0.090269000	-1.543473000	1.537786000
C	0.579872000	-2.163626000	0.430131000
C	1.259010000	-1.414999000	-0.589849000
C	1.909472000	-2.156948000	-1.606464000
C	1.869581000	-3.538582000	-1.634665000
C	1.186864000	-4.266905000	-0.647109000
C	0.561931000	-3.571236000	0.371294000
N	-0.853149000	2.243390000	1.495123000

C	-1.532558000	3.267350000	1.110977000
C	-1.418166000	3.869290000	-0.186704000
C	-0.394401000	3.481730000	-1.118857000
C	-0.312548000	4.223527000	-2.324982000
C	-1.192901000	5.250204000	-2.608093000
C	-2.200790000	5.616237000	-1.700254000
C	-2.289770000	4.929014000	-0.504355000
O	0.462394000	2.532712000	-0.897424000
Al	0.283831000	1.118996000	0.290249000
O	1.328945000	-0.117654000	-0.609756000
H	-2.233636000	3.741329000	1.815117000
H	-0.506978000	-2.225742000	2.289889000
H	-3.049980000	5.199938000	0.228294000
H	-2.888095000	6.424781000	-1.929850000
H	-1.101075000	5.783360000	-3.552528000
H	2.386553000	-4.065902000	-2.433846000
H	1.158348000	-5.351900000	-0.678022000
H	0.465990000	3.939518000	-3.027011000
H	2.446413000	-1.596167000	-2.366720000
H	0.020466000	-4.103000000	1.153228000
H	-1.755169000	2.190311000	3.410134000
H	0.022241000	2.033630000	3.340585000
H	-0.673235000	-0.215540000	3.755361000
H	-2.074070000	-0.069897000	2.664291000
C	-5.722748000	-1.218455000	0.227266000
C	-6.359847000	-0.085854000	0.991187000
O	-4.963975000	-1.998000000	1.180784000
H	-5.048784000	-0.822724000	-0.537220000
H	-6.972504000	0.525487000	0.323934000
C	-6.701677000	-2.202317000	-0.389680000
H	-6.170130000	-3.017847000	-0.885363000
H	-7.370917000	-2.618019000	0.367840000
Cl	-7.734538000	-1.422722000	-1.639662000
H	-5.572821000	0.537798000	1.417862000
H	-6.994446000	-0.468773000	1.797075000
C	-3.214137000	-2.396022000	-0.516810000
C	-3.703060000	-2.405444000	0.894818000
C	-2.550437000	-1.387493000	-1.093255000
C	-2.305054000	-0.105368000	-0.360265000
O	-1.256685000	0.524772000	-0.741391000
O	-3.083286000	0.206516000	0.559598000
O	-3.044378000	-2.905112000	1.783098000
H	-3.284897000	-3.364226000	-1.012068000
H	-2.075790000	-1.521677000	-2.063115000

C	5.245498000	-1.111578000	-0.038989000
C	4.723681000	-2.082981000	0.989619000
O	6.107314000	-0.154350000	0.619823000
H	4.410587000	-0.598101000	-0.530376000
H	4.073107000	-2.822659000	0.512929000
C	6.175664000	-1.717868000	-1.080091000
H	6.518235000	-0.953428000	-1.780963000
H	7.044070000	-2.167522000	-0.592650000
Cl	5.399181000	-3.010051000	-2.061754000
H	4.149108000	-1.534954000	1.739413000
H	5.550360000	-2.604642000	1.483719000
C	4.846220000	1.715531000	-0.379389000
C	5.911629000	1.187155000	0.520755000
C	3.550983000	1.854013000	-0.078029000
C	2.991319000	1.394029000	1.233380000
O	1.723929000	1.592172000	1.410020000
O	3.754803000	0.894535000	2.066812000
O	6.711035000	1.924388000	1.055969000
H	5.227570000	2.097742000	-1.325986000
H	2.865231000	2.319994000	-0.78267000065
B_INT13	E = -2346.402316		
C	1.137939000	0.796058000	2.716435000
C	0.403951000	-0.528169000	2.523618000
N	0.860800000	-1.127582000	1.268979000
C	0.410943000	-2.302785000	0.945924000
C	0.688987000	-2.969172000	-0.289763000
C	1.602546000	-2.441954000	-1.259479000
C	1.830469000	-3.206349000	-2.424934000
C	1.169078000	-4.402894000	-2.636761000
C	0.256514000	-4.909087000	-1.696157000
C	0.032560000	-4.194422000	-0.536902000
N	1.391796000	1.394382000	1.418396000
C	1.344085000	2.677057000	1.254450000
C	1.740342000	3.350865000	0.056869000
C	2.415346000	2.664778000	-1.006701000
C	2.878634000	3.443580000	-2.090866000
C	2.657775000	4.807391000	-2.141919000
C	1.982933000	5.476945000	-1.107324000
C	1.545455000	4.746207000	-0.021777000
O	2.648675000	1.378886000	-0.996515000
Al	1.713070000	0.116151000	-0.070339000
O	2.248021000	-1.316064000	-1.090791000
H	1.008492000	3.307360000	2.089861000
H	-0.250675000	-2.829876000	1.642620000

H	1.035297000	5.240034000	0.804003000
H	1.817149000	6.547937000	-1.158361000
H	3.014764000	5.370300000	-3.001092000
H	1.357536000	-4.957138000	-3.553251000
H	-0.262518000	-5.844182000	-1.879206000
H	3.398317000	2.926284000	-2.891329000
H	2.527493000	-2.809578000	-3.156682000
H	-0.676458000	-4.551367000	0.208040000
H	0.573886000	1.471560000	3.371413000
H	2.115814000	0.614674000	3.186042000
H	0.586949000	-1.200850000	3.370984000
H	-0.674403000	-0.353301000	2.450173000
C	-4.610850000	0.547238000	0.570167000
C	-4.673182000	1.613668000	1.633195000
O	-4.117222000	-0.655802000	1.215462000
H	-3.913463000	0.840893000	-0.219860000
H	-5.066379000	2.542376000	1.213295000
C	-5.955360000	0.172082000	-0.028680000
H	-5.851435000	-0.657911000	-0.731514000
H	-6.669190000	-0.107790000	0.749991000
Cl	-6.664016000	1.542846000	-0.942723000
H	-3.669753000	1.799712000	2.019392000
H	-5.320932000	1.302113000	2.458224000
C	-2.982834000	-1.299741000	-0.874829000
C	-3.220164000	-1.446201000	0.594900000
C	-1.991415000	-0.579993000	-1.411329000
C	-1.087635000	0.224844000	-0.541111000
O	0.115285000	0.348017000	-1.000820000
O	-1.509321000	0.657445000	0.540061000
O	-2.696532000	-2.345953000	1.221094000
H	-3.588412000	-1.956908000	-1.497541000
H	-1.763328000	-0.646572000	-2.472066000
C	4.102140000	-1.527928000	1.218519000
C	4.589132000	-1.800174000	2.603429000
O	3.521667000	-0.212787000	1.028530000
H	3.507982000	-2.306609000	0.740806000
H	5.246924000	-2.673643000	2.612096000
C	4.731850000	-0.560423000	0.325111000
H	4.596856000	-0.658187000	-0.748889000
H	5.597750000	0.003543000	0.666341000
H	3.749853000	-2.011784000	3.273494000
H	5.142038000	-0.945494000	3.001416000

85

B_TS6 E = -3379.0972146 Imaginary Frequency= 439.7317

C	-0.983069000	1.290564000	2.729118000
C	-0.689549000	-0.153381000	2.324815000
N	-0.119663000	-0.152992000	0.978764000
C	0.251452000	-1.285022000	0.470820000
C	0.743026000	-1.456446000	-0.866516000
C	0.945529000	-0.358431000	-1.766556000
C	1.404764000	-0.665295000	-3.070859000
C	1.650651000	-1.968715000	-3.461543000
C	1.461885000	-3.042442000	-2.574438000
C	1.012565000	-2.772923000	-1.295602000
N	-1.359686000	2.046767000	1.551904000
C	-2.307092000	2.920524000	1.584440000
C	-2.632796000	3.795396000	0.496848000
C	-1.799168000	3.900646000	-0.669626000
C	-2.156970000	4.882869000	-1.627533000
C	-3.273600000	5.680102000	-1.465519000
C	-4.094464000	5.564195000	-0.330583000
C	-3.756004000	4.635518000	0.634146000
O	-0.736823000	3.180321000	-0.864588000
Al	-0.336307000	1.555997000	-0.097343000
O	0.753642000	0.885346000	-1.433787000
H	-2.898022000	3.035939000	2.505702000
H	0.164257000	-2.196502000	1.072304000
H	-4.361783000	4.533867000	1.534426000
H	-4.968773000	6.196635000	-0.211572000
H	-3.518871000	6.411754000	-2.232821000
H	2.005059000	-2.162054000	-4.471938000
H	1.662669000	-4.061849000	-2.888842000
H	-1.521369000	4.973097000	-2.503282000
H	1.559174000	0.166384000	-3.752524000
H	0.838154000	-3.582478000	-0.587110000
H	-1.758025000	1.335152000	3.506164000
H	-0.071021000	1.756436000	3.127715000
H	0.001382000	-0.622798000	3.037680000
H	-1.618684000	-0.733992000	2.307100000
C	-5.463869000	-2.253092000	0.472837000
C	-6.046846000	-1.517140000	1.652283000
O	-4.320613000	-2.994832000	0.959637000
H	-5.137094000	-1.540678000	-0.289781000
H	-6.935226000	-0.957340000	1.349562000
C	-6.378330000	-3.298826000	-0.141148000
H	-5.873331000	-3.827220000	-0.952962000
H	-6.706341000	-4.023149000	0.608575000
Cl	-7.857173000	-2.557081000	-0.846730000

H	-5.302314000	-0.817495000	2.035215000
H	-6.327064000	-2.216361000	2.446681000
C	-3.109550000	-2.528295000	-1.140196000
C	-3.145200000	-2.973017000	0.286439000
C	-2.838655000	-1.282008000	-1.543508000
C	-2.627881000	-0.186403000	-0.547906000
O	-1.862113000	0.757962000	-0.959445000
O	-3.149160000	-0.297791000	0.575358000
O	-2.164029000	-3.447785000	0.818851000
H	-3.157890000	-3.344695000	-1.860230000
H	-2.659803000	-1.064405000	-2.594195000
C	2.451477000	1.642242000	0.894618000
C	3.140926000	1.674618000	2.227350000
O	1.184955000	2.302135000	0.850511000
H	2.427223000	0.639079000	0.445285000
H	4.202678000	1.435507000	2.099582000
C	2.649045000	2.731076000	-0.038618000
H	2.366668000	2.609550000	-1.074242000
H	2.878497000	3.713629000	0.345418000
H	2.700399000	0.945365000	2.915839000
H	3.069213000	2.673026000	2.669167000
C	5.002550000	-1.450756000	1.138516000
C	5.815650000	-1.165739000	2.376136000
O	5.914726000	-1.936763000	0.134494000
H	4.519122000	-0.533766000	0.792265000
H	5.170443000	-0.820956000	3.188917000
C	3.962798000	-2.548268000	1.280298000
H	3.407779000	-2.679602000	0.346182000
H	4.422233000	-3.495967000	1.571365000
Cl	2.742129000	-2.150129000	2.550794000
H	6.540481000	-0.383820000	2.140715000
H	6.346815000	-2.064055000	2.707665000
C	4.665656000	-0.752080000	-1.641616000
C	5.868253000	-1.479487000	-1.148557000
C	4.381055000	0.545916000	-1.481536000
C	5.203891000	1.472900000	-0.621123000
O	4.763994000	2.663598000	-0.482574000
O	6.231079000	0.988171000	-0.098641000
O	6.737444000	-1.842547000	-1.910814000
H	4.037639000	-1.365428000	-2.289379000
H	3.505313000	0.952054000	-1.989046000

85

B_INT14 E = -3379.1404365

C	-0.869668000	1.406931000	2.751958000

C	-0.490542000	-0.027001000	2.375970000
N	0.009636000	-0.046204000	1.004675000
C	0.359751000	-1.178181000	0.492699000
C	0.733664000	-1.363059000	-0.885668000
C	0.878419000	-0.270484000	-1.805754000
C	1.182977000	-0.598817000	-3.151950000
C	1.341885000	-1.910916000	-3.563249000
C	1.219098000	-2.974854000	-2.654299000
C	0.917007000	-2.685789000	-1.334511000
N	-1.322032000	2.107172000	1.569717000
C	-2.347337000	2.885029000	1.588721000
C	-2.755995000	3.701334000	0.481604000
C	-1.936122000	3.860840000	-0.690741000
C	-2.394583000	4.782926000	-1.668717000
C	-3.583600000	5.470334000	-1.521310000
C	-4.386853000	5.300243000	-0.380501000
C	-3.955108000	4.430201000	0.602832000
O	-0.810107000	3.249352000	-0.875589000
Al	-0.220131000	1.696437000	-0.054074000
O	0.793449000	0.978113000	-1.457406000
H	-2.947424000	2.963621000	2.508936000
H	0.324927000	-2.087287000	1.107007000
H	-4.546656000	4.289365000	1.507723000
H	-5.319774000	5.845051000	-0.271406000
H	-3.901739000	6.155874000	-2.304840000
H	1.572224000	-2.117501000	-4.606631000
H	1.347525000	-4.001988000	-2.981760000
H	-1.772982000	4.914391000	-2.549653000
H	1.282524000	0.226636000	-3.851959000
H	0.777162000	-3.489205000	-0.611469000
H	-1.629308000	1.415949000	3.546540000
H	0.019851000	1.941814000	3.110013000
H	0.265116000	-0.420768000	3.069262000
H	-1.374737000	-0.672820000	2.422415000
C	-5.213285000	-2.359622000	0.536684000
C	-5.754308000	-1.666718000	1.761325000
O	-4.049063000	-3.110079000	0.952741000
H	-4.921640000	-1.620153000	-0.213999000
H	-6.657530000	-1.104010000	1.512308000
C	-6.142287000	-3.394215000	-0.074448000
H	-5.666814000	-3.886528000	-0.925816000
H	-6.429057000	-4.148751000	0.662379000
Cl	-7.661162000	-2.650148000	-0.689256000
H	-4.998283000	-0.974240000	2.134707000

H	-5.998133000	-2.393422000	2.543232000
C	-2.901310000	-2.538093000	-1.158172000
C	-2.892329000	-3.042308000	0.248068000
C	-2.679019000	-1.269394000	-1.519651000
C	-2.475135000	-0.197983000	-0.492574000
O	-1.771279000	0.789089000	-0.897918000
O	-2.947929000	-0.381668000	0.646108000
O	-1.895060000	-3.535907000	0.732628000
H	-2.934988000	-3.328710000	-1.907684000
H	-2.532973000	-1.010738000	-2.566607000
C	2.497750000	1.973233000	0.765031000
C	3.175532000	2.048002000	2.128217000
O	1.204635000	2.434843000	0.826348000
H	2.544269000	0.908652000	0.427836000
H	4.226834000	1.736196000	2.089419000
C	3.236291000	2.806629000	-0.283435000
H	2.746283000	2.702885000	-1.257847000
H	3.214460000	3.860755000	0.004034000
H	2.649462000	1.398057000	2.835818000
H	3.134594000	3.074359000	2.510948000
C	4.890141000	-1.394253000	1.175017000
C	5.820318000	-1.135804000	2.333443000
O	5.701706000	-1.961855000	0.118810000
H	4.428400000	-0.457645000	0.848584000
H	5.270023000	-0.707846000	3.174392000
C	3.796511000	-2.415969000	1.437510000
H	3.134628000	-2.508495000	0.569289000
H	4.225322000	-3.392328000	1.675742000
Cl	2.765918000	-1.934009000	2.829232000
H	6.594270000	-0.431262000	2.022480000
H	6.297548000	-2.064582000	2.661462000
C	4.311916000	-0.903026000	-1.630949000
C	5.494544000	-1.692179000	-1.191965000
C	4.110116000	0.415239000	-1.499203000
C	5.064739000	1.278638000	-0.767672000
O	4.654014000	2.494190000	-0.369932000
O	6.207889000	0.923370000	-0.525117000
O	6.226801000	-2.201294000	-2.011775000
H	3.591860000	-1.489432000	-2.202483000
H	3.212879000	0.853616000	-1.926777000

94

B_INT4 (Full mode) E = -2402.9622361

C	0.705399000	4.008742000	0.177560000
C	-0.580993000	4.030595000	-0.639389000

N	-1.203010000	2.724027000	-0.494022000
C	-2.496984000	2.612531000	-0.553226000
C	-3.218349000	1.384173000	-0.530844000
C	-2.543008000	0.125784000	-0.623816000
C	-3.331947000	-1.048989000	-0.799208000
C	-4.717088000	-0.912693000	-0.757304000
C	-5.403444000	0.309141000	-0.604401000
C	-4.626100000	1.447672000	-0.523236000
N	1.307270000	2.701931000	-0.026145000
C	2.589102000	2.585149000	-0.188261000
C	3.286722000	1.351229000	-0.342257000
C	2.599713000	0.097874000	-0.322128000
C	3.369104000	-1.093754000	-0.460094000
C	4.747089000	-0.963531000	-0.618953000
C	5.443358000	0.260475000	-0.651535000
C	4.684154000	1.405810000	-0.508380000
O	1.296422000	0.038034000	-0.183535000
Al	-0.038528000	1.226366000	0.150616000
O	-1.234644000	0.054618000	-0.589829000
H	3.202259000	3.497774000	-0.201625000
H	-3.098575000	3.526591000	-0.657684000
H	5.156129000	2.388955000	-0.517210000
H	5.326492000	-1.873424000	-0.724109000
H	-5.312349000	-1.812859000	-0.858067000
H	-5.090090000	2.431745000	-0.448030000
H	1.383568000	4.825245000	-0.100981000
H	0.462801000	4.102769000	1.244209000
H	-1.251014000	4.840715000	-0.325285000
H	-0.338377000	4.190153000	-1.699343000
C	-0.087054000	0.707905000	2.987061000
C	1.418361000	0.603632000	3.172406000
O	-0.439235000	1.434356000	1.846619000
H	-0.514132000	1.216552000	3.868215000
H	1.675220000	0.108658000	4.113383000
C	-0.767651000	-0.646642000	2.847968000
H	-1.847933000	-0.522359000	2.744153000
H	-0.388568000	-1.166574000	1.963322000
Cl	-0.494874000	-1.749701000	4.251607000
H	1.859813000	1.605252000	3.174629000
H	1.872651000	0.031618000	2.355143000
C	6.959309000	0.353165000	-0.829544000
C	2.699404000	-2.470915000	-0.425253000
C	1.658622000	-2.584301000	-1.549610000
H	0.869432000	-1.838188000	-1.448465000

H	2.129391000	-2.460886000	-2.531685000
H	1.192233000	-3.576497000	-1.525153000
C	2.021318000	-2.685441000	0.936275000
H	2.750515000	-2.646181000	1.753439000
H	1.260416000	-1.927613000	1.125801000
H	1.532693000	-3.666253000	0.967830000
C	3.708575000	-3.605458000	-0.618071000
H	4.222407000	-3.544027000	-1.584001000
H	4.466316000	-3.627598000	0.172935000
H	3.179614000	-4.563218000	-0.589214000
C	7.275274000	1.166245000	-2.092724000
H	6.865114000	2.179739000	-2.033529000
H	8.358638000	1.254948000	-2.232930000
H	6.855909000	0.689486000	-2.984751000
C	7.612387000	-1.021958000	-0.966857000
H	7.449879000	-1.643239000	-0.079711000
H	7.236025000	-1.569188000	-1.837762000
H	8.693557000	-0.906555000	-1.093334000
C	7.573463000	1.060688000	0.386433000
H	7.172301000	2.071858000	0.510488000
H	7.371197000	0.507649000	1.309410000
H	8.660197000	1.147045000	0.273614000
C	-6.928780000	0.406843000	-0.553102000
C	-7.352136000	1.031703000	0.783605000
H	-6.929369000	2.033540000	0.911615000
H	-8.443113000	1.121533000	0.839022000
H	-7.018797000	0.420756000	1.628718000
C	-7.602453000	-0.959371000	-0.680517000
H	-7.366480000	-1.445895000	-1.633067000
H	-7.309712000	-1.637260000	0.128281000
H	-8.689691000	-0.841183000	-0.632809000
C	-7.425100000	1.298049000	-1.700148000
H	-7.143470000	0.882988000	-2.673481000
H	-8.517042000	1.388223000	-1.672706000
H	-7.008844000	2.308668000	-1.636344000
C	-2.672572000	-2.403345000	-1.079590000
C	-1.912513000	-2.310144000	-2.413020000
H	-1.158246000	-1.521006000	-2.389525000
H	-1.404709000	-3.258255000	-2.625722000
H	-2.603097000	-2.105476000	-3.238750000
C	-1.700774000	-2.808639000	0.038791000
H	-0.852977000	-2.125333000	0.101805000
H	-2.201540000	-2.835493000	1.013495000
H	-1.308236000	-3.812976000	-0.160606000

C -3.705461000 -3.525188000 -1.213658000
H -4.280323000 -3.670591000 -0.292181000
H -4.410455000 -3.346874000 -2.033044000
H -3.187756000 -4.465305000 -1.428438000
105
B_TS2 (Full mode) E = -3056.354351 Imaginary Frequency= 408.6085
C 0.672516000 -0.380097000 -3.965703000
C -0.543512000 -1.279789000 -3.718736000
N -1.209815000 -0.806738000 -2.524425000
C -2.493888000 -0.818464000 -2.408881000
C -3.227450000 -0.447633000 -1.231733000
C -2.592877000 -0.131748000 0.018440000
C -3.432319000 0.056709000 1.162287000
C -4.811816000 0.013748000 0.983062000
C -5.452458000 -0.239668000 -0.244631000
C -4.630705000 -0.488141000 -1.327916000
N 1.323765000 -0.181937000 -2.689930000
C 2.596721000 -0.289968000 -2.537582000
C 3.291042000 -0.111157000 -1.290590000
C 2.646659000 0.372828000 -0.101736000
C 3.460136000 0.583220000 1.056609000
C 4.807180000 0.235352000 0.993191000
C 5.444526000 -0.281511000 -0.149248000
C 4.662099000 -0.419214000 -1.282347000
O 1.370837000 0.647336000 -0.065789000
Al 0.003537000 0.061061000 -1.176916000
O -1.296388000 -0.056820000 0.137884000
H 3.224972000 -0.538593000 -3.408253000
H -3.102210000 -1.160614000 -3.261301000
H 5.098217000 -0.793915000 -2.210183000
H 5.405128000 0.371462000 1.887172000
H -5.436810000 0.173587000 1.854074000
H -5.060338000 -0.733145000 -2.300985000
H 1.349239000 -0.812839000 -4.716953000
H 0.325819000 0.600515000 -4.318035000
H -1.215382000 -1.300797000 -4.588856000
H -0.193864000 -2.304713000 -3.528582000
C -0.269702000 2.964793000 -1.678616000
C 1.195185000 3.332182000 -1.893199000
O -0.553005000 1.634075000 -1.924158000
H -0.874574000 3.595123000 -2.361377000
H 1.360804000 4.408531000 -1.775505000
C -0.749085000 3.242095000 -0.261205000
H -1.804189000 2.981208000 -0.159272000

H	-0.166736000	2.657550000	0.454928000
Cl	-0.597800000	4.985323000	0.252302000
H	1.499889000	3.042646000	-2.904788000
H	1.839550000	2.805090000	-1.182195000
C	0.791597000	-2.357195000	0.503854000
C	2.071190000	-3.112969000	0.702574000
O	0.585844000	-1.823211000	-0.807968000
H	0.595768000	-1.622132000	1.290366000
H	1.994900000	-3.706448000	1.618654000
C	-0.382896000	-3.027734000	-0.004363000
H	-1.360307000	-2.589317000	0.136274000
H	-0.270451000	-3.900804000	-0.629576000
Cl	-1.011402000	-4.716282000	1.795951000
H	2.925050000	-2.430067000	0.781205000
H	2.244638000	-3.795711000	-0.135379000
C	6.916713000	-0.691911000	-0.172209000
C	7.611383000	-0.438534000	1.165785000
H	8.661711000	-0.743784000	1.102448000
H	7.589830000	0.621272000	1.442318000
H	7.149767000	-1.008524000	1.979045000
C	7.661788000	0.099176000	-1.256199000
H	8.717136000	-0.198051000	-1.297194000
H	7.230246000	-0.071746000	-2.247772000
H	7.618165000	1.175296000	-1.057404000
C	7.018115000	-2.190729000	-0.490424000
H	6.488782000	-2.787728000	0.259820000
H	6.577106000	-2.423161000	-1.465325000
H	8.066037000	-2.515567000	-0.510284000
C	2.858036000	1.185383000	2.332091000
C	2.355817000	2.607085000	2.036252000
H	1.637925000	2.613736000	1.216000000
H	1.861407000	3.030332000	2.919522000
H	3.186722000	3.268084000	1.763116000
C	1.697723000	0.328082000	2.860938000
H	0.861253000	0.300271000	2.161373000
H	2.020640000	-0.702236000	3.052837000
H	1.329873000	0.743761000	3.807138000
C	3.889382000	1.291123000	3.459357000
H	4.276727000	0.310497000	3.759280000
H	4.739594000	1.927390000	3.187460000
H	3.413126000	1.738947000	4.337964000
C	-6.972914000	-0.278458000	-0.401598000
C	-7.409751000	0.777844000	-1.426413000
H	-6.945249000	0.604421000	-2.402716000

H	-8.498168000	0.758564000	-1.565344000
H	-7.125089000	1.783654000	-1.099852000
C	-7.700131000	0.002246000	0.913489000
H	-8.783507000	-0.028787000	0.752255000
H	-7.457691000	-0.741301000	1.680157000
H	-7.452091000	0.991500000	1.313160000
C	-7.405284000	-1.664181000	-0.901517000
H	-7.109487000	-2.446696000	-0.195355000
H	-8.494521000	-1.709123000	-1.026770000
H	-6.949390000	-1.902986000	-1.867909000
C	-2.820991000	0.252611000	2.555439000
C	-3.898089000	0.409492000	3.633212000
H	-4.531169000	1.289175000	3.464993000
H	-4.544840000	-0.472284000	3.703030000
H	-3.411866000	0.535691000	4.606369000
C	-1.943536000	1.512780000	2.604395000
H	-1.096128000	1.433445000	1.922761000
H	-2.516005000	2.410370000	2.340593000
H	-1.550377000	1.651856000	3.619334000
C	-1.978398000	-0.978254000	2.926771000
H	-2.570460000	-1.898785000	2.913598000
H	-1.144763000	-1.120515000	2.238274000
H	-1.561895000	-0.857766000	3.934578000

114

B_INT10 (Full mode) E = -3435.6904414

C	-0.943180000	0.472828000	-3.685869000
C	0.461127000	0.951433000	-3.308684000
N	0.934002000	0.118763000	-2.217883000
C	2.168874000	-0.244355000	-2.138589000
C	2.745735000	-0.994661000	-1.056224000
C	2.012267000	-1.361119000	0.119759000
C	2.737754000	-1.976004000	1.195859000
C	4.092137000	-2.224829000	1.016694000
C	4.822969000	-1.894576000	-0.143407000
C	4.124001000	-1.267632000	-1.156329000
N	-1.669543000	0.247355000	-2.454163000
C	-2.890485000	0.618916000	-2.297663000
C	-3.656402000	0.422624000	-1.095273000
C	-3.168496000	-0.341447000	0.016879000
C	-4.003432000	-0.440485000	1.172745000
C	-5.247385000	0.185189000	1.153968000
C	-5.751170000	0.916219000	0.063202000
C	-4.928793000	1.014483000	-1.046921000
O	-2.024203000	-0.965897000	-0.017107000

Al	-0.517302000	-0.584543000	-1.014045000
O	0.743371000	-1.109282000	0.257799000
H	-3.407347000	1.136051000	-3.122171000
H	2.865984000	0.065112000	-2.928334000
H	-5.254135000	1.576388000	-1.924537000
H	-5.867232000	0.104802000	2.040292000
H	4.639106000	-2.698470000	1.828113000
H	4.626480000	-0.942094000	-2.065592000
H	-1.452405000	1.195012000	-4.340222000
H	-0.869630000	-0.491854000	-4.206309000
H	1.144967000	0.927352000	-4.168083000
H	0.403816000	1.986334000	-2.941555000
C	-1.069843000	-3.311948000	-2.048189000
C	-2.568969000	-3.183634000	-2.302414000
O	-0.393622000	-2.108404000	-2.034812000
H	-0.651110000	-3.954985000	-2.849487000
H	-3.041833000	-4.165725000	-2.411983000
C	-0.751451000	-3.979195000	-0.718412000
H	0.328632000	-4.070989000	-0.589883000
H	-1.162153000	-3.389427000	0.103977000
Cl	-1.434623000	-5.663747000	-0.532409000
H	-2.734954000	-2.613720000	-3.223061000
H	-3.062488000	-2.652546000	-1.482087000
C	2.389923000	5.117731000	0.134578000
C	1.589930000	6.241534000	-0.472721000
O	3.038006000	4.426143000	-0.958957000
H	1.724197000	4.426881000	0.658137000
H	1.098763000	6.824273000	0.310554000
C	3.524149000	5.559143000	1.042990000
H	4.081341000	4.695688000	1.413943000
H	4.208541000	6.231370000	0.519114000
Cl	2.919517000	6.443169000	2.489910000
H	0.827049000	5.816260000	-1.126614000
H	2.234608000	6.908965000	-1.054225000
C	2.770087000	2.278269000	0.225724000
C	3.082325000	3.071246000	-0.999835000
C	1.559923000	1.824077000	0.569920000
C	0.330406000	2.132870000	-0.232461000
O	-0.587089000	1.238882000	-0.214404000
O	0.302428000	3.205114000	-0.864719000
O	3.505905000	2.544068000	-2.007913000
H	3.655049000	1.935915000	0.765388000
H	1.461548000	1.127464000	1.398010000
C	6.315046000	-2.210278000	-0.207857000

C	6.933687000	-1.778487000	-1.536744000
H	6.464176000	-2.287798000	-2.385247000
H	6.837465000	-0.699422000	-1.697496000
H	8.001704000	-2.023651000	-1.549939000
C	7.047978000	-1.476693000	0.924894000
H	6.665206000	-1.770010000	1.908157000
H	8.121897000	-1.701433000	0.904057000
H	6.924550000	-0.392263000	0.831568000
C	6.532270000	-3.721167000	-0.042602000
H	7.601666000	-3.965760000	-0.070952000
H	6.132893000	-4.083308000	0.910488000
H	6.033110000	-4.278277000	-0.842398000
C	2.044215000	-2.286162000	2.529506000
C	1.572034000	-0.974291000	3.176575000
H	2.414292000	-0.288357000	3.331210000
H	0.822841000	-0.478207000	2.556042000
H	1.116560000	-1.176354000	4.154212000
C	0.832417000	-3.212316000	2.345947000
H	0.056185000	-2.736575000	1.745835000
H	1.117393000	-4.153021000	1.860857000
H	0.402425000	-3.457748000	3.325447000
C	2.992582000	-2.968744000	3.519928000
H	3.367515000	-3.925805000	3.138949000
H	3.854715000	-2.340250000	3.771918000
H	2.452815000	-3.173004000	4.450941000
C	-7.124671000	1.587806000	0.068320000
C	-6.960592000	3.100611000	-0.135583000
H	-6.360643000	3.541944000	0.667026000
H	-6.458545000	3.325261000	-1.082113000
H	-7.937643000	3.600385000	-0.147734000
C	-7.978785000	1.023525000	-1.075973000
H	-8.125805000	-0.055578000	-0.960513000
H	-8.965764000	1.503018000	-1.098550000
H	-7.505100000	1.189657000	-2.048964000
C	-7.877291000	1.362221000	1.379815000
H	-8.052057000	0.297898000	1.571366000
H	-7.333723000	1.773472000	2.237242000
H	-8.854125000	1.857147000	1.337771000
C	-3.520191000	-1.217150000	2.401919000
C	-2.177862000	-0.655645000	2.899523000
H	-1.397442000	-0.738270000	2.141662000
H	-2.274638000	0.402159000	3.170780000
H	-1.852085000	-1.205966000	3.791780000
C	-4.510272000	-1.123894000	3.566584000

H	-4.670719000	-0.088619000	3.889686000
H	-5.485577000	-1.559592000	3.319935000
H	-4.111024000	-1.679228000	4.422386000
C	-3.363980000	-2.705263000	2.053244000
H	-4.323908000	-3.139537000	1.750336000
H	-2.656361000	-2.849468000	1.236714000
H	-2.998411000	-3.264926000	2.923521000

124

B_TS4 (Full mode) E = -3628.76250105 Imaginary Frequency= 412.6185

C	-0.802674000	0.666330000	-3.538315000
C	0.576471000	1.050848000	-2.994888000
N	0.948546000	0.062554000	-2.003614000
C	2.156553000	-0.389256000	-1.931883000
C	2.639770000	-1.325986000	-0.960980000
C	1.834406000	-1.799465000	0.129996000
C	2.483392000	-2.565305000	1.152342000
C	3.828748000	-2.881252000	0.988878000
C	4.618628000	-2.473801000	-0.104381000
C	3.997485000	-1.682028000	-1.051692000
N	-1.615899000	0.292953000	-2.401308000
C	-2.847646000	0.660494000	-2.288169000
C	-3.691214000	0.337234000	-1.174174000
C	-3.293826000	-0.590346000	-0.155901000
C	-4.160061000	-0.750103000	0.974175000
C	-5.351871000	-0.039558000	0.994058000
C	-5.784809000	0.835407000	-0.026183000
C	-4.930860000	1.005984000	-1.097064000
O	-2.200138000	-1.292480000	-0.246157000
Al	-0.595452000	-0.807612000	-1.040640000
O	0.569136000	-1.521961000	0.226382000
H	-3.294532000	1.293090000	-3.072422000
H	2.906846000	-0.013571000	-2.644828000
H	-5.182850000	1.684304000	-1.911078000
H	-6.001843000	-0.154387000	1.858264000
H	4.305453000	-3.473842000	1.761736000
H	4.564501000	-1.283904000	-1.895160000
H	-1.251379000	1.481910000	-4.124623000
H	-0.696400000	-0.219228000	-4.179706000
H	1.322312000	1.132230000	-3.798457000
H	0.510113000	2.034805000	-2.500448000
C	-1.068511000	-3.357220000	-2.444235000
C	-2.542023000	-3.212247000	-2.810965000
O	-0.415932000	-2.149316000	-2.267946000
H	-0.574029000	-3.916905000	-3.263530000

H	-2.993428000	-4.182690000	-3.043995000
C	-0.856159000	-4.151099000	-1.162381000
H	0.209595000	-4.241095000	-0.942950000
H	-1.354606000	-3.658967000	-0.323691000
Cl	-1.513471000	-5.850666000	-1.222750000
H	-2.637697000	-2.565678000	-3.690176000
H	-3.105716000	-2.756583000	-1.990427000
C	0.137257000	1.810206000	0.505677000
C	0.739382000	1.782312000	1.884338000
O	-0.712838000	0.740445000	0.186876000
H	0.902975000	2.016081000	-0.263944000
H	1.103304000	2.778275000	2.161063000
C	-1.132934000	2.499096000	0.295448000
H	-1.449378000	2.734317000	-0.713349000
H	-1.883296000	2.446237000	1.070958000
H	1.572849000	1.073588000	1.923325000
H	-0.013673000	1.476954000	2.617237000
C	3.537435000	4.111203000	0.773725000
C	3.906693000	3.933286000	2.224145000
O	3.711493000	5.507478000	0.451690000
H	2.494568000	3.823105000	0.619712000
H	3.831329000	2.878759000	2.504828000
C	4.420098000	3.355274000	-0.203435000
H	4.165732000	3.623139000	-1.232420000
H	5.479205000	3.563128000	-0.029251000
Cl	4.185861000	1.577561000	-0.063192000
H	3.219933000	4.513489000	2.843612000
H	4.930226000	4.274299000	2.410286000
C	1.878310000	5.346161000	-1.204583000
C	2.848107000	6.137443000	-0.387009000
C	0.694635000	4.866847000	-0.802696000
C	0.212674000	4.981598000	0.620755000
O	-0.923874000	4.455460000	0.887217000
O	0.959520000	5.580008000	1.418463000
O	2.999244000	7.323007000	-0.587820000
H	2.160190000	5.291618000	-2.256336000
H	0.033119000	4.400664000	-1.534791000
C	6.099412000	-2.827085000	-0.246760000
C	6.322942000	-3.585747000	-1.562465000
H	6.011517000	-2.989561000	-2.426477000
H	7.383768000	-3.834132000	-1.692569000
H	5.749248000	-4.518525000	-1.581134000
C	6.934132000	-1.538202000	-0.267306000
H	6.803301000	-0.969665000	0.659539000

H	8.000890000	-1.768618000	-0.381460000
H	6.642632000	-0.882482000	-1.094392000
C	6.600879000	-3.703230000	0.901354000
H	6.507575000	-3.199390000	1.869586000
H	6.054422000	-4.650946000	0.960042000
H	7.660410000	-3.939277000	0.752538000
C	1.720722000	-2.965341000	2.421667000
C	1.280181000	-1.693019000	3.163591000
H	2.148740000	-1.086524000	3.447247000
H	0.620093000	-1.082417000	2.544648000
H	0.739304000	-1.958642000	4.080547000
C	0.481118000	-3.816748000	2.104875000
H	-0.255583000	-3.254084000	1.530544000
H	0.747412000	-4.717256000	1.539191000
H	0.006468000	-4.138305000	3.040693000
C	2.596026000	-3.777428000	3.380640000
H	2.935830000	-4.719087000	2.933877000
H	3.478531000	-3.218401000	3.712163000
H	2.012757000	-4.028087000	4.273100000
C	-3.741468000	-1.653583000	2.137376000
C	-2.391579000	-1.184125000	2.705873000
H	-1.595445000	-1.218940000	1.960261000
H	-2.463182000	-0.151871000	3.070636000
H	-2.102481000	-1.821080000	3.551648000
C	-3.635084000	-3.113330000	1.668605000
H	-4.605974000	-3.483593000	1.319213000
H	-2.918158000	-3.223151000	0.854089000
H	-3.307988000	-3.753159000	2.497757000
C	-4.755646000	-1.614853000	3.284088000
H	-4.874799000	-0.605196000	3.694559000
H	-5.743579000	-1.979350000	2.978482000
H	-4.404863000	-2.260880000	4.096142000
C	-7.121000000	1.559013000	0.118391000
C	-7.442262000	2.415647000	-1.106052000
H	-6.694301000	3.200762000	-1.260811000
H	-7.494597000	1.812425000	-2.019540000
H	-8.412894000	2.906298000	-0.974738000
C	-7.075918000	2.474291000	1.350936000
H	-8.032042000	2.995870000	1.482314000
H	-6.877586000	1.906445000	2.266150000
H	-6.287148000	3.227363000	1.252198000
C	-8.250936000	0.535061000	0.298273000
H	-8.094905000	-0.088628000	1.184589000
H	-9.216188000	1.042706000	0.415890000

H	-8.319616000	-0.131980000	-0.567957000

84

B1 E =-2209.8431603

C	0.577963285	-3.826449921	-0.717106511
C	-0.665251199	-3.942309168	0.156159739
N	-1.307351084	-2.642610437	0.107330629
C	-2.596365171	-2.531328901	0.005335499
C	-3.300035157	-1.294346580	-0.071531999
C	-2.602349134	-0.053653107	-0.152377355
C	-3.359173578	1.148569731	-0.280230329
C	-4.742533137	1.035750978	-0.295131502
C	-5.458126079	-0.180102083	-0.211857922
C	-4.711826108	-1.334414198	-0.109487502
N	1.186665434	-2.529868568	-0.431785770
C	2.474320324	-2.389237512	-0.588399253
C	3.214848437	-1.194129484	-0.383340821
C	2.566644063	0.038782895	-0.057905485
C	3.372342693	1.198219696	0.135027711
C	4.748053657	1.063890163	-0.023823922
C	5.409229572	-0.136030510	-0.362911878
C	4.616228518	-1.252257605	-0.535420440
O	1.265665199	0.108086795	0.036840160
Al	-0.002630305	-1.147872026	0.374100558
O	-1.291757300	0.000737111	-0.139449742
H	3.054356745	-3.266884235	-0.904973219
H	-3.202893513	-3.446999279	-0.033565747
H	5.060602330	-2.214733689	-0.791223452
H	5.358046966	1.947124955	0.127757628
H	-5.326419312	1.948431144	-0.377651426
H	-5.190586466	-2.309686043	-0.048020066
Cl	0.172690956	-1.401057183	2.520690248
H	1.280971879	-4.649140073	-0.539832286
H	0.285303414	-3.855992310	-1.775746950
H	-1.328735163	-4.749153721	-0.178664462
H	-0.370018444	-4.140103786	1.195596258
C	6.926589558	-0.230788392	-0.530952252
C	7.253606228	-0.709609837	-1.952319189
H	8.337613546	-0.793989959	-2.090413469
H	6.816647852	-1.692175045	-2.158401892
H	6.869627490	-0.010940771	-2.702866136
C	7.489416606	-1.241112026	0.478403970
H	8.576099127	-1.331635901	0.367897645
H	7.277455241	-0.932048879	1.507058805
H	7.057714759	-2.237114288	0.335770769

C	7.620656057	1.112333054	-0.305441793
H	7.280001801	1.873790434	-1.015186012
H	7.455147458	1.494558125	0.707431685
H	8.700865734	0.997080461	-0.440169331
C	2.733741876	2.530917328	0.533926313
C	3.778619574	3.637978507	0.693481800
H	4.511591814	3.407996603	1.474744226
H	4.320965409	3.834967087	-0.238328869
H	3.275879428	4.566447296	0.981944849
C	2.012412716	2.378238780	1.883375543
H	1.232633056	1.615742647	1.851502367
H	2.719855990	2.104872275	2.673814220
H	1.546004192	3.329969174	2.164553784
C	1.736382184	2.989282480	-0.541030438
H	2.231620423	3.106870951	-1.511767239
H	0.913266638	2.283354029	-0.655907125
H	1.312820634	3.961815393	-0.262567861
C	-2.654890942	2.502580220	-0.399626351
C	-1.805594584	2.530660418	-1.680736032
H	-1.291542750	3.495224175	-1.766812559
H	-1.049022134	1.743894898	-1.682195614
H	-2.437599201	2.407215071	-2.567477798
C	-1.763304347	2.760710845	0.826340120
H	-0.957907692	2.029810022	0.912069803
H	-1.308030415	3.755134910	0.747587057
H	-2.351409012	2.734905294	1.750293058
C	-3.657096347	3.656177886	-0.486990869
H	-4.309176018	3.574746054	-1.363937643
H	-4.289337009	3.723531228	0.405542226
H	-3.111114318	4.600801774	-0.573392765
C	-6.983859766	-0.154465289	-0.241617720
C	-7.579337632	-1.558074349	-0.143315983
H	-7.264500241	-2.193551041	-0.978276650
H	-7.295391334	-2.056394701	0.789852454
H	-8.672256189	-1.501913429	-0.166991735
C	-7.504291779	0.677600820	0.938961220
H	-7.136691817	1.708103926	0.902177739
H	-8.599412136	0.716663785	0.927556890
H	-7.189721516	0.246577845	1.894938087
C	-7.460433039	0.484428288	-1.553736493
H	-8.555147412	0.522602916	-1.586696737
H	-7.091081713	1.509119823	-1.663990229
H	-7.114637668	-0.088703698	-2.420330406

88

C1 E= -2362.2714679

C	-0.757192000	5.900895000	-0.175387000
C	-1.433701000	4.688542000	-0.158794000
C	-0.720689000	3.485669000	-0.141089000
C	0.691838000	3.512039000	-0.118898000
C	1.359694000	4.740841000	-0.130136000
C	0.639229000	5.926827000	-0.163305000
N	1.285877000	2.244042000	-0.078432000
C	2.586657000	2.088432000	-0.153801000
C	3.269343000	0.849353000	-0.154836000
C	2.565081000	-0.392494000	-0.205913000
C	3.319138000	-1.602039000	-0.293316000
C	4.701447000	-1.495449000	-0.285241000
C	5.424621000	-0.279810000	-0.223026000
C	4.685994000	0.879254000	-0.171235000
N	-1.277385000	2.195668000	-0.113811000
C	-2.563550000	2.011560000	-0.310304000
C	-3.252506000	0.778671000	-0.243219000
C	-2.568861000	-0.455111000	-0.003085000
C	-3.340517000	-1.647454000	0.118779000
C	-4.716973000	-1.545678000	-0.050447000
C	-5.412359000	-0.347789000	-0.328986000
C	-4.655299000	0.800995000	-0.415161000
O	-1.267539000	-0.489351000	0.080516000
Al	0.018310000	0.751853000	0.394590000
O	1.257966000	-0.428270000	-0.205223000
H	-3.184489000	2.882243000	-0.542418000
H	3.221258000	2.975835000	-0.241244000
H	-5.127425000	1.764722000	-0.608017000
H	-5.300627000	-2.454452000	0.041492000
H	5.281804000	-2.412909000	-0.330845000
H	5.169333000	1.853271000	-0.134342000
H	-1.319630000	6.829344000	-0.181571000
H	-2.518550000	4.686475000	-0.137394000
H	1.166616000	6.875689000	-0.164531000
H	2.443662000	4.778612000	-0.099808000
Cl	0.138731000	0.887092000	2.543704000
C	-6.929603000	-0.293064000	-0.515258000
C	-2.667382000	-2.979687000	0.454451000
C	-1.655299000	-3.355078000	-0.637709000
H	-2.147053000	-3.449952000	-1.612700000
H	-0.859649000	-2.615035000	-0.722593000
H	-1.194951000	-4.321783000	-0.400510000
C	-1.957558000	-2.869400000	1.814211000

H	-1.212072000	-2.072490000	1.829848000
H	-2.680494000	-2.672776000	2.613431000
H	-1.449579000	-3.812900000	2.046101000
C	-3.680423000	-4.122324000	0.555320000
H	-4.425183000	-3.949521000	1.340166000
H	-4.210020000	-4.292798000	-0.388914000
H	-3.152339000	-5.047823000	0.805339000
C	-7.541645000	0.631194000	0.546603000
H	-7.147124000	1.649665000	0.471931000
H	-8.629465000	0.687846000	0.426196000
H	-7.330796000	0.266730000	1.557176000
C	-7.251791000	0.263805000	-1.908874000
H	-8.335867000	0.320368000	-2.059314000
H	-6.845847000	1.271440000	-2.045049000
H	-6.833161000	-0.372335000	-2.695559000
C	-7.580148000	-1.670400000	-0.385941000
H	-7.202350000	-2.374388000	-1.135098000
H	-7.418533000	-2.109351000	0.604318000
H	-8.661285000	-1.582927000	-0.533035000
C	2.613195000	-2.955110000	-0.395652000
C	1.800288000	-3.004562000	-1.699242000
H	1.263549000	-3.957690000	-1.771218000
H	1.065812000	-2.198914000	-1.749461000
H	2.461603000	-2.924765000	-2.569391000
C	1.692463000	-3.177643000	0.814934000
H	2.261438000	-3.140677000	1.750460000
H	0.896456000	-2.434236000	0.870647000
H	1.225772000	-4.167532000	0.745788000
C	3.610335000	-4.115699000	-0.431134000
H	4.283729000	-4.059516000	-1.293826000
H	4.220039000	-4.165156000	0.478093000
H	3.060553000	-5.058943000	-0.508076000
C	6.949901000	-0.317924000	-0.221852000
C	7.554230000	1.083596000	-0.154707000
H	7.261889000	1.694808000	-1.015523000
H	7.254778000	1.612667000	0.756383000
H	8.646848000	1.018721000	-0.153629000
C	7.438795000	-1.117370000	0.994285000
H	7.063735000	-2.145795000	0.981708000
H	8.533469000	-1.165179000	1.006238000
H	7.108723000	-0.654580000	1.929919000
C	7.446595000	-1.000837000	-1.504035000
H	8.541313000	-1.047818000	-1.513673000
H	7.072055000	-2.025956000	-1.590102000

H	7.122665000	-0.452398000	-2.394662000
90			
Al	E= -2438.4969869		
Cl	-0.080122000	0.629854000	2.217994000
Al	-0.005937000	0.676338000	0.065077000
O	1.265721000	-0.495801000	-0.421168000
O	-1.282702000	-0.353052000	-0.680140000
N	1.305364000	2.180486000	-0.353968000
N	-1.281631000	2.238258000	-0.205081000
C	2.566033000	-0.480394000	-0.215414000
C	-2.575270000	-0.362967000	-0.415021000
C	-3.319487000	-1.568192000	-0.575123000
C	3.275082000	-1.708355000	-0.061971000
C	0.751384000	3.405248000	-0.541565000
C	2.658732000	2.023859000	-0.450199000
C	-0.708167000	3.448033000	-0.390630000
C	4.646345000	-1.653380000	0.162171000
C	-2.634237000	2.123836000	-0.098629000
C	4.678206000	0.718911000	0.085042000
C	3.293458000	0.744443000	-0.179712000
C	5.378649000	-0.458962000	0.262164000
C	1.499221000	4.525899000	-0.866975000
C	-3.260990000	0.816979000	0.000137000
C	-1.442110000	4.623678000	-0.432802000
C	3.443365000	3.135549000	-0.836205000
C	2.873243000	4.373121000	-1.035544000
C	-4.657474000	-1.569036000	-0.194593000
C	-2.827464000	4.534662000	-0.309134000
C	-4.609635000	0.729523000	0.397736000
C	-3.418409000	3.298263000	-0.162627000
C	-5.326462000	-0.450324000	0.329684000
H	5.177111000	-2.588970000	0.291720000
H	5.210060000	1.659603000	0.197253000
H	-5.210660000	-2.495909000	-0.288161000
H	-5.095873000	1.613532000	0.801981000
H	-4.497233000	3.218567000	-0.131211000
H	-3.439640000	5.430459000	-0.351932000
H	-0.955861000	5.582889000	-0.560126000
H	1.022761000	5.486568000	-1.017414000
H	3.487723000	5.216893000	-1.334714000
H	4.503415000	2.999975000	-1.004161000
C	6.875344000	-0.421073000	0.575279000
C	-6.777017000	-0.496451000	0.812168000
C	-7.613685000	0.527857000	0.032668000

H	-8.655799000	0.512741000	0.372024000
H	-7.237475000	1.547105000	0.170810000
H	-7.603161000	0.311918000	-1.040817000
C	-7.407544000	-1.875399000	0.617891000
H	-8.443664000	-1.861683000	0.971172000
H	-7.422635000	-2.174114000	-0.435807000
H	-6.878001000	-2.650299000	1.182045000
C	-6.826556000	-0.148435000	2.306493000
H	-6.241959000	-0.860859000	2.897294000
H	-6.424045000	0.850850000	2.501670000
H	-7.859420000	-0.168812000	2.673166000
C	-2.648911000	-2.841471000	-1.097225000
C	-3.645567000	-3.993410000	-1.251092000
H	-4.086268000	-4.293905000	-0.294171000
H	-4.460323000	-3.748412000	-1.941908000
H	-3.122644000	-4.865878000	-1.655593000
C	-1.569202000	-3.290150000	-0.104991000
H	-0.786358000	-2.539052000	-0.005997000
H	-1.997548000	-3.473628000	0.887021000
H	-1.102993000	-4.221628000	-0.448625000
C	-2.021138000	-2.596140000	-2.478522000
H	-2.787378000	-2.310734000	-3.208284000
H	-1.263049000	-1.812781000	-2.447531000
H	-1.544932000	-3.516401000	-2.837165000
C	2.533703000	-3.047249000	-0.065507000
C	1.730198000	-3.230787000	-1.362852000
H	1.231257000	-4.207163000	-1.353018000
H	0.969191000	-2.462318000	-1.494911000
H	2.397973000	-3.208638000	-2.231928000
C	1.619460000	-3.102852000	1.169172000
H	0.932631000	-2.256308000	1.216841000
H	1.025087000	-4.024224000	1.162022000
H	2.220610000	-3.096301000	2.085155000
C	3.494072000	-4.237053000	0.029315000
H	4.199738000	-4.267570000	-0.808777000
H	4.068417000	-4.238721000	0.962056000
H	2.914334000	-5.165329000	0.005396000
C	7.099842000	0.318399000	1.901667000
H	8.168308000	0.363157000	2.142512000
H	6.725752000	1.346571000	1.858885000
H	6.586408000	-0.186106000	2.726476000
C	7.617375000	0.323468000	-0.543555000
H	7.261089000	1.353676000	-0.649491000
H	8.691865000	0.366540000	-0.331422000

H	7.481612000	-0.176117000	-1.508418000
C	7.476026000	-1.821490000	0.697317000
H	7.019308000	-2.393223000	1.511988000
H	7.361134000	-2.397892000	-0.226907000
H	8.547620000	-1.747307000	0.908813000

74

D1 E = -2353.0322195

Cl	-0.042538000	0.643653000	2.318618000
Al	-0.001310000	0.698238000	0.163988000
O	1.272042000	-0.458725000	-0.346161000
O	-1.282396000	-0.333853000	-0.564365000
N	1.292661000	2.216153000	-0.269244000
N	-1.293607000	2.254111000	-0.078777000
C	2.577624000	-0.432700000	-0.158419000
C	-2.573248000	-0.353916000	-0.279060000
C	-3.304937000	-1.568939000	-0.425442000
C	3.293942000	-1.657376000	-0.009274000
C	0.726280000	3.437824000	-0.437802000
C	2.645383000	2.072709000	-0.390082000
C	-0.730761000	3.469081000	-0.261815000
C	4.670718000	-1.606383000	0.186519000
C	-2.643736000	2.131970000	0.047242000
C	4.687985000	0.791396000	0.090469000
C	3.297259000	0.796944000	-0.138457000
C	5.370789000	-0.395044000	0.252781000
C	1.459487000	4.566903000	-0.768273000
C	-3.263558000	0.820472000	0.144170000
C	-1.472144000	4.640905000	-0.280592000
C	3.415047000	3.193229000	-0.778309000
C	2.831731000	4.427426000	-0.959746000
C	-4.641583000	-1.592697000	-0.037030000
C	-2.854420000	4.543064000	-0.134063000
C	-4.609055000	0.734388000	0.550270000
C	-3.435575000	3.301718000	0.009812000
C	-5.289321000	-0.462866000	0.479613000
H	5.214843000	-2.533439000	0.316465000
H	5.250725000	1.712068000	0.190863000
H	-5.192770000	-2.521253000	-0.117590000
H	-5.133916000	1.591123000	0.957225000
H	-4.512732000	3.215473000	0.062302000
H	-3.472227000	5.435710000	-0.155640000
H	-0.993253000	5.604111000	-0.405539000
H	0.972701000	5.524598000	-0.903729000
H	3.434607000	5.278741000	-1.261044000

H	4.473846000	3.067851000	-0.959872000
C	-2.625023000	-2.838199000	-0.944712000
C	-3.608779000	-4.003324000	-1.080471000
H	-4.037378000	-4.301124000	-0.117063000
H	-4.431148000	-3.774622000	-1.767982000
H	-3.078921000	-4.873214000	-1.481166000
C	-1.532311000	-3.264833000	0.043002000
H	-0.759134000	-2.502366000	0.129820000
H	-1.950882000	-3.445785000	1.039633000
H	-1.056835000	-4.192808000	-0.297249000
C	-2.012554000	-2.595958000	-2.333270000
H	-2.788839000	-2.327775000	-3.058880000
H	-1.265870000	-1.801504000	-2.314698000
H	-1.526545000	-3.512769000	-2.687513000
C	2.558363000	-2.998960000	0.013689000
C	1.734411000	-3.197706000	-1.268356000
H	1.238716000	-4.175356000	-1.240125000
H	0.969998000	-2.431868000	-1.395142000
H	2.387878000	-3.182665000	-2.148350000
C	1.666706000	-3.042845000	1.265218000
H	0.979977000	-2.196455000	1.316283000
H	1.074379000	-3.965402000	1.278431000
H	2.284424000	-3.025349000	2.169966000
C	3.524127000	-4.184601000	0.104646000
H	4.213433000	-4.223805000	-0.746711000
H	4.114675000	-4.173849000	1.027328000
H	2.947365000	-5.114907000	0.102777000
O	6.717897000	-0.297219000	0.485710000
O	-6.586022000	-0.456174000	0.922978000
C	-7.287757000	-1.680054000	0.882223000
H	-6.798087000	-2.449701000	1.493411000
H	-8.277329000	-1.474142000	1.290237000
H	-7.393147000	-2.055741000	-0.144424000
C	7.424842000	-1.500888000	0.694526000
H	8.461285000	-1.214825000	0.873692000
H	7.045534000	-2.048963000	1.567179000
H	7.379668000	-2.157707000	-0.184455000

118

E1 E = -3205.3794186

Cl	-0.024236000	1.633076000	1.969713000
Al	-0.010499000	1.876599000	-0.171946000
O	1.237753000	0.738978000	-0.793805000
O	-1.324711000	0.911344000	-0.942372000
N	1.305198000	3.395639000	-0.495000000

N	-1.276707000	3.462757000	-0.282143000
C	2.541931000	0.724540000	-0.617516000
C	-2.605716000	0.892654000	-0.628225000
C	-3.362485000	-0.307027000	-0.811516000
C	3.243366000	-0.521693000	-0.580796000
C	0.760869000	4.637180000	-0.564915000
C	2.656038000	3.236023000	-0.625262000
C	-0.695027000	4.679098000	-0.383622000
C	4.606964000	-0.491234000	-0.343352000
C	-2.628411000	3.352393000	-0.149838000
C	4.674966000	1.881340000	-0.225569000
C	3.282619000	1.933980000	-0.480641000
C	5.350416000	0.684776000	-0.146961000
C	1.514693000	5.775845000	-0.800641000
C	-3.267320000	2.049499000	-0.121903000
C	-1.416953000	5.860798000	-0.316739000
C	3.445804000	4.372277000	-0.920654000
C	2.885123000	5.627074000	-1.000886000
C	-4.675057000	-0.317880000	-0.371286000
C	-2.800312000	5.774746000	-0.169931000
C	-4.605580000	1.953047000	0.329173000
C	-3.400607000	4.536282000	-0.104642000
C	-5.316852000	0.778854000	0.229840000
H	5.140214000	-1.433983000	-0.257721000
H	5.210854000	2.805338000	-0.036555000
H	-5.240867000	-1.242176000	-0.448593000
H	-5.062775000	2.815194000	0.803583000
H	-4.478949000	4.464699000	-0.051753000
H	-3.403854000	6.676485000	-0.129432000
H	-0.923469000	6.822624000	-0.378986000
H	1.046120000	6.750305000	-0.857164000
H	3.503823000	6.489009000	-1.231680000
H	4.502309000	4.244307000	-1.113954000
C	-2.716079000	-1.591116000	-1.361086000
C	-1.650036000	-2.044142000	-0.353937000
H	-0.862015000	-1.295896000	-0.294019000
H	-2.061808000	-2.167995000	0.651822000
H	-1.197213000	-2.992373000	-0.663518000
C	-2.032396000	-1.340652000	-2.718898000
H	-2.725484000	-0.927073000	-3.455485000
H	-1.205864000	-0.640227000	-2.613640000
H	-1.642716000	-2.283317000	-3.116908000
C	2.504725000	-1.870421000	-0.637995000
C	1.544472000	-1.961232000	-1.839005000

H	1.108436000	-2.965689000	-1.884293000
H	0.738149000	-1.234767000	-1.756595000
H	2.061376000	-1.779346000	-2.786730000
C	1.700117000	-1.988781000	0.665128000
H	0.984097000	-1.168199000	0.745077000
H	1.143651000	-2.933764000	0.701657000
H	2.353940000	-1.934392000	1.543471000
C	6.851059000	0.552436000	0.111420000
C	-6.754403000	0.590511000	0.712766000
C	7.071466000	-0.499372000	1.203532000
C	7.907746000	-1.605851000	1.040260000
C	6.400619000	-0.356651000	2.426014000
C	8.075581000	-2.536856000	2.067135000
H	8.430408000	-1.762009000	0.101373000
C	6.565137000	-1.280192000	3.451754000
H	5.720056000	0.482781000	2.560503000
C	7.408435000	-2.377926000	3.276354000
H	8.724556000	-3.394485000	1.911223000
H	6.027760000	-1.148739000	4.386825000
H	7.534979000	-3.105531000	4.073257000
C	-6.809381000	-0.662143000	1.594230000
C	-7.709761000	-1.708407000	1.381734000
C	-5.913449000	-0.772568000	2.666939000
C	-7.719559000	-2.827530000	2.216729000
H	-8.407949000	-1.668276000	0.550680000
C	-5.919934000	-1.883965000	3.501458000
H	-5.184321000	0.019397000	2.831174000
C	-6.827657000	-2.920181000	3.279523000
H	-8.423984000	-3.632407000	2.024087000
H	-5.209695000	-1.946516000	4.321500000
H	-6.832119000	-3.793495000	3.925862000
C	7.475052000	1.871969000	0.580690000
H	7.399307000	2.645153000	-0.192344000
H	6.996235000	2.248137000	1.490517000
H	8.537123000	1.726460000	0.799081000
C	7.516238000	0.153216000	-1.213319000
H	7.305869000	0.911024000	-1.974084000
H	8.604095000	0.075017000	-1.109784000
H	7.136944000	-0.804618000	-1.585247000
C	-7.244631000	1.783382000	1.541994000
H	-6.607212000	1.962730000	2.413763000
H	-7.272623000	2.700218000	0.941996000
H	-8.259082000	1.594220000	1.905123000
C	-7.655323000	0.464649000	-0.523313000

H	-8.709602000	0.364345000	-0.243309000
H	-7.558001000	1.361041000	-1.143232000
H	-7.381711000	-0.397722000	-1.140787000
C	3.515535000	-3.017024000	-0.769656000
C	3.795088000	-3.904212000	0.273706000
C	4.233024000	-3.178342000	-1.965398000
C	4.753127000	-4.910134000	0.133213000
H	3.272115000	-3.812429000	1.220533000
C	5.190913000	-4.175723000	-2.110922000
H	4.057967000	-2.490116000	-2.789545000
C	5.457965000	-5.050513000	-1.057297000
H	4.950586000	-5.579686000	0.966487000
H	5.732629000	-4.269629000	-3.049087000
H	6.206832000	-5.830388000	-1.165817000
C	-3.786632000	-2.673131000	-1.546731000
C	-3.963321000	-3.710663000	-0.625322000
C	-4.679251000	-2.610892000	-2.626891000
C	-4.987107000	-4.646791000	-0.771998000
H	-3.304321000	-3.791092000	0.233231000
C	-5.703690000	-3.540754000	-2.778617000
H	-4.589595000	-1.804984000	-3.351122000
C	-5.864689000	-4.566920000	-1.848337000
H	-5.099916000	-5.435985000	-0.033178000
H	-6.380546000	-3.461192000	-3.625720000
H	-6.664807000	-5.293307000	-1.962780000

66

F1 E = -2322.4689162

Cl	-0.033998000	0.597644000	2.365830000
Al	-0.001455000	0.612891000	0.214111000
O	1.281492000	-0.545462000	-0.277590000
O	-1.275233000	-0.452990000	-0.484976000
N	1.275841000	2.130275000	-0.256709000
N	-1.309796000	2.146460000	-0.058172000
C	2.585952000	-0.506087000	-0.108990000
C	-2.564832000	-0.478905000	-0.210607000
C	-3.280466000	-1.711636000	-0.347528000
C	3.308400000	-1.732905000	0.050903000
C	0.697460000	3.344616000	-0.438953000
C	2.629017000	1.997538000	-0.382594000
C	-0.758996000	3.364565000	-0.258099000
C	4.683721000	-1.670173000	0.226027000
C	-2.658111000	2.013468000	0.074119000
C	4.691700000	0.739543000	0.089967000
C	3.293003000	0.731043000	-0.119414000

C	5.348658000	-0.447778000	0.255500000
C	1.418844000	4.475922000	-0.786702000
C	-3.265112000	0.698090000	0.188397000
C	-1.511330000	4.528995000	-0.287904000
C	3.386696000	3.120157000	-0.787508000
C	2.791435000	4.346771000	-0.981563000
C	-4.616360000	-1.741757000	0.029565000
C	-2.891905000	4.420146000	-0.134870000
C	-4.617600000	0.606769000	0.587417000
C	-3.460876000	3.175187000	0.025699000
C	-5.252295000	-0.602064000	0.514221000
H	5.272034000	-2.568881000	0.369322000
H	5.256191000	1.660256000	0.167925000
H	-5.192982000	-2.657802000	-0.022762000
H	-5.158886000	1.459101000	0.979459000
H	-4.537200000	3.082163000	0.082418000
H	-3.518207000	5.306470000	-0.164369000
H	-1.042167000	5.495151000	-0.425736000
H	0.923005000	5.427334000	-0.932618000
H	3.385457000	5.199578000	-1.295492000
H	4.445813000	3.004615000	-0.973444000
C	-2.582846000	-2.980577000	-0.839490000
C	-3.559839000	-4.151654000	-0.968784000
H	-3.998786000	-4.435712000	-0.006309000
H	-4.374857000	-3.936678000	-1.668928000
H	-3.021880000	-5.025234000	-1.349647000
C	-1.502229000	-3.382707000	0.171503000
H	-0.738887000	-2.611058000	0.263755000
H	-1.935884000	-3.558820000	1.162322000
H	-1.011175000	-4.308532000	-0.151080000
C	-1.954198000	-2.754531000	-2.223417000
H	-2.722566000	-2.502627000	-2.962946000
H	-1.211294000	-1.956496000	-2.209458000
H	-1.459328000	-3.674130000	-2.556656000
C	2.580115000	-3.075981000	0.105775000
C	1.742913000	-3.300940000	-1.163031000
H	1.257860000	-4.282777000	-1.113294000
H	0.968725000	-2.545461000	-1.292272000
H	2.384944000	-3.293816000	-2.051336000
C	1.704462000	-3.102182000	1.369105000
H	1.011952000	-2.260123000	1.414032000
H	1.119345000	-4.028373000	1.405312000
H	2.332859000	-3.065411000	2.265631000
C	3.558456000	-4.249916000	0.206029000

H	4.237100000	-4.297826000	-0.653052000
H	4.160783000	-4.215285000	1.120027000
H	2.991241000	-5.185552000	0.228975000
F	-6.543601000	-0.704595000	0.918490000
F	6.687854000	-0.444210000	0.475257000
